# Genetic Predictors of Response to Serotonergic and Noradrenergic Antidepressants in Major Depressive Disorder: A Genome-Wide Analysis of Individual-Level Data and a Meta-Analysis

**DOI:** 10.1371/journal.pmed.1001326

**Published:** 2012-10-16

**Authors:** Katherine E. Tansey, Michel Guipponi, Nader Perroud, Guido Bondolfi, Enrico Domenici, David Evans, Stephanie K. Hall, Joanna Hauser, Neven Henigsberg, Xiaolan Hu, Borut Jerman, Wolfgang Maier, Ole Mors, Michael O'Donovan, Tim J. Peters, Anna Placentino, Marcella Rietschel, Daniel Souery, Katherine J. Aitchison, Ian Craig, Anne Farmer, Jens R. Wendland, Alain Malafosse, Peter Holmans, Glyn Lewis, Cathryn M. Lewis, Tine Bryan Stensbøl, Shitij Kapur, Peter McGuffin, Rudolf Uher

**Affiliations:** 1 Institute of Psychiatry, King's College London, London, United Kingdom; 2 Department of Genetic Medicine and Laboratories, University Hospitals of Geneva, Geneva, Switzerland; 3 Department of Psychiatry, University of Geneva, Geneva, Switzerland; 4 Center of Excellence for Drug Discovery in Psychiatry, GlaxoSmithKline Medicines Research Centre, Verona, Italy; 5 Pharma Research and Early Development, F. Hoffmann–La Roche, Basel, Switzerland; 6 Medical Research Council CAiTE Centre, School of Social and Community Medicine, University of Bristol, Bristol, United Kingdom; 7 Molecular Medicine, Pfizer, Groton, Connecticut, United States of America; 8 Laboratory of Psychiatric Genetics, Poznan University of Medical Sciences, Poznan, Poland; 9 Croatian Institute for Brain Research, Medical School, University of Zagreb, Zagreb, Croatia; 10 Department of Molecular and Biomedical Sciences, Jozef Stefan Institute, Ljubljana, Slovenia; 11 Institute of Public Health of the Republic of Slovenia, Ljubljana, Slovenia; 12 Department of Psychiatry, University of Bonn, Bonn, Germany; 13 Centre for Psychiatric Research, Aarhus University Hospital, Risskov, Denmark; 14 Medical Research Council Centre for Neuropsychiatric Genetics and Genomics, Department of Psychological Medicine and Neurology, School of Medicine, Cardiff University, Cardiff, United Kingdom; 15 School of Clinical Sciences, University of Bristol, Bristol, United Kingdom; 16 Psychiatric Unit 23, Department of Mental Health, Spedali Civili Hospital and Biological Psychiatry Unit, Centro San Giovanni di Dio Fatebenefratelli, Brescia, Italy; 17 Central Institute of Mental Health, Division of Genetic Epidemiology in Psychiatry, Mannheim, Germany; 18 Department of Psychiatry, Erasme Academic Hospital, Université Libre de Bruxelles, Brussels, Belgium; 19 Department of Psychiatry, University of Alberta, Edmonton, Alberta, Canada; 20 School of Social and Community Medicine, University of Bristol, Bristol, United Kingdom; 21 Discovery Pharmacology Research, H. Lundbeck A/S, Copenhagen, Denmark; 22 Department of Psychiatry, Dalhousie University, Halifax, Nova Scotia, Canada; University of Western Sydney, Australia

## Abstract

**Background:**

It has been suggested that outcomes of antidepressant treatment for major depressive disorder could be significantly improved if treatment choice is informed by genetic data. This study aims to test the hypothesis that common genetic variants can predict response to antidepressants in a clinically meaningful way.

**Methods and Findings:**

The NEWMEDS consortium, an academia–industry partnership, assembled a database of over 2,000 European-ancestry individuals with major depressive disorder, prospectively measured treatment outcomes with serotonin reuptake inhibiting or noradrenaline reuptake inhibiting antidepressants and available genetic samples from five studies (three randomized controlled trials, one part-randomized controlled trial, and one treatment cohort study). After quality control, a dataset of 1,790 individuals with high-quality genome-wide genotyping provided adequate power to test the hypotheses that antidepressant response or a clinically significant differential response to the two classes of antidepressants could be predicted from a single common genetic polymorphism. None of the more than half million genetic markers significantly predicted response to antidepressants overall, serotonin reuptake inhibitors, or noradrenaline reuptake inhibitors, or differential response to the two types of antidepressants (genome-wide significance *p*<5×10^−8^). No biological pathways were significantly overrepresented in the results. No significant associations (genome-wide significance *p*<5×10^−8^) were detected in a meta-analysis of NEWMEDS and another large sample (STAR*D), with 2,897 individuals in total. Polygenic scoring found no convergence among multiple associations in NEWMEDS and STAR*D.

**Conclusions:**

No single common genetic variant was associated with antidepressant response at a clinically relevant level in a European-ancestry cohort. Effects specific to particular antidepressant drugs could not be investigated in the current study.

*Please see later in the article for the Editors' Summary*

## Abstract 2

Testing whether genetic information could inform the selection of the best drug for patients with depression, Rudolf Uher and colleagues searched for genetic variants that could predict clinically meaningful responses to two major groups of antidepressants.

## Editors' Summary

**Background:**

Genetic and environmental factors can influence a person's response to medications. Taking advantage of the recent advancements in genetics, scientists are working to match specific gene variations with responses to particular medications. Knowing whether a patient is likely to respond to a drug or have serious side effects would allow doctors to select the best treatment up front. Right now, there are only a handful of examples where a patient's version of a particular gene predicts their response to a particular drug. Some scientists believe that there will be many more such matches between genetic variants and treatment responses. Others think that because the action of most drugs is influenced by many different genes, a variant in one of those genes is unlikely to have measurable effect in most cases.

**Why Was This Study Done?:**

One of the areas where patients' responses to available drugs vary widely is severe depression (or major depressive disorder). Prescription of an antidepressant is often the first step in treating the disease. However, less than half of patients get well taking the first antidepressant prescribed. Those who don't respond to the first drug need to, together with their doctors, try multiple courses of treatment to find the right drug and the right dose for them. For some patients none of the existing drugs work well.

To see whether genetic information could help improve the choice of antidepressant, researchers from universities and the pharmaceutical industry joined forces in this large study. They examined two ways to use genetic information to improve the treatment of depression. First, they searched all genes for common genetic variants that could predict which patients would not respond to the two major groups of antidepressants (serotonin reuptake inhibitors, or SRIs, and noradrenaline reuptake inhibitors, or NRIs). They hoped that this would help with the development of new drugs that could help these patients. Second, they looked for common genetic variants in all genes that could identify patients who responded to one of the two major groups of antidepressants. Such predictors would make it possible to know which drug to prescribe for which patient.

**What Did the Researchers Do and Find?:**

The researchers selected 1,790 patients with severe depression who had participated in one of several research studies; 1,222 of the patients had been treated with an SRI, the remaining 568 with an NRI, and it was recorded how well the drugs worked for each patient. The researchers also had a detailed picture of the genetic make-up of each patient, with information for over half a million genetic variants. They then looked for an association between genetic variants and responses to drugs.

They found not a single genetic variant that could predict clearly whether a person would respond to antidepressants in general, to one of the two main groups (SRIs and NRIs), or much better to one than the other. They also didn't find any combination of variants in groups of genes that work together that could predict responses. Combining their data with those from another large study did not yield any robust predictors either.

**What Do These Findings Mean?:**

This study was large enough that it should have been possible to find common genetic variants that by themselves could predict a clinically meaningful response to SRIs and/or NRIs, had such variants existed. The fact that the study failed to find such variants suggests that such variants do not exist. It is still possible, however, that variants that are less common could predict response, or that combinations of variants could. To find those, if they do exist, even larger studies will need to be done.

**Additional Information:**

Please access these websites via the online version of this summary at http://dx.doi.org/10.1371/journal.pmed.1001326

- The National Institute of General Medical Sciences at the US National Institutes of Health has a fact sheet on personalized medicine
- PubMed Health at the US National Library of Medicine has a page on major depressive disorder
- Wikipedia has pages on major depressive disorder and pharmacogenetics, the study of how genetic variation affects response to certain drugs (note that Wikipedia is a free online encyclopedia that anyone can edit)
- The UK National Health Service has comprehensive information pages on depression

## Introduction

Major depressive disorder (MDD) is a disabling illness, affecting a high proportion of individuals at some point in their life [1]. Prescription of antidepressants is the most common initial step in treating MDD, but less than half of individuals achieve remission of symptoms with their first antidepressant [2],[3]. It has been proposed that common genetic variants could be used to personalize psychiatric treatment and significantly improve outcomes [4]–[7]. However, to date there has not been a robust, well-replicated finding of sufficient effect size to be worth translating into a clinical setting.

Identification of genetic determinants of antidepressant response has the potential to improve the treatment of MDD in two important ways. First, genetic and molecular predictors of poor treatment outcome with available antidepressants can provide targets for the development of novel therapeutic agents that may be effective for the type of depression that is resistant to current treatments. Second, for many individuals with MDD, delay in reaching recovery is avoidable, since they have the potential to respond to one of the currently available treatments. If a predictor of differential outcome with alternative treatments is identified, a clinician could use it to select the antidepressant that is most likely to alleviate depression in a given individual. For both applications, the clinical implications are predicated on the effect size of the prediction. A consensus criterion has been set for what size of difference in depressive symptoms is clinically meaningful: a panel of experts and service users has concluded that a difference in outcome equal or greater than three points on the Hamilton Rating Scale for Depression is noticeable to the patients and their relatives and can be considered as clinically significant [8],[9]. This criterion is equal to 6.33% of variance in outcome explained, which can be applied to assess whether a genetic biomarker provides clinically significant prediction [9]. Currently, no clinically significant predictor is available [10]. The aims of this NEWMEDS study address the two potential avenues for using genomic information to improve treatment of depression.

The first aim is to identify common genetic polymorphisms that predict unfavourable outcome of treatment with currently available antidepressants. Addressing this issue in the large combined NEWMEDS sample will substantially expand on the evidence from the first genome-wide studies on outcomes for single-drug treatment [11] or naturalistic inpatient treatment [12] of depression and could provide novel targets for the development of new treatments.

The second aim is to obtain predictors of differential outcomes of treatment with antidepressants with different modes of action in the largest comparative pharmacogenetic study to date. Specifically, we aim to identify common genetic variants that differentially predict outcome of treatment with antidepressants that act primarily through the inhibition of serotonin reuptake (serotonin reuptake inhibitors [SRIs]) or act primarily through the inhibition of norepinephrine reuptake (noradrenaline reuptake inhibitors [NRIs]). For the first time, to our knowledge, these two aims will be pursued in a sample that is large enough to provide sufficient power to ensure interpretable results.

## Methods

### Samples

As part of the NEWMEDS consortium (http://www.newmeds-europe.com) [13], three studies conducted by academic institutions (GENDEP, a part-randomized open study of two active antidepressants, *n* = 868; GENPOD, a randomized controlled trial of two active antidepressants, *n* = 601; and GODS, a treatment cohort of severe depression, *n* = 131) [14]–[16] and two studies by pharmaceutical industry members of the European Federation of Pharmaceutical Industries and Associations (active comparator arms from randomized controlled trials by Pfizer, *n* = 355, and GlaxoSmithKline, *n* = 191) were combined to obtain a sample of 2,146 adult individuals (1,205 women and 941 men; see Table 1 in Text S1 for description by contributing study) diagnosed with unipolar MDD according to the Diagnostic and Statistical Manual of Mental Disorders–IV and the International Classification of Diseases–10, with prospective data on outcome of treatment with SRI or NRI antidepressants. Diagnoses of schizophrenia, schizoaffective disorder, or bipolar disorder, or current alcohol or drug dependence constituted exclusion criteria. All individuals were treated for 6 to 12 wk with either an antidepressant that acts primarily through blocking the reuptake of serotonin (SRIs: escitalopram, citalopram, paroxetine, sertraline, fluoxetine) or an antidepressant that acts primarily through blocking the reuptake of norepinephrine (NRIs: nortriptyline, reboxetine). These groupings are based on previous evidence that at least some genetic predictors of treatment response are specific to antidepressants that block serotonin or noradrenaline reuptake [14]. The two largest studies (GENDEP and GENPOD) included an open-label randomized comparison between an SRI (escitalopram, citalopram) and an NRI (nortriptyline, reboxetine). Detailed information on individual studies, including inclusion and exclusion criteria for each study, can be found in Text S1 (section 1.1).

### Genotyping

From each study, individuals with self-reported white European ancestry, available high-quality blood DNA samples, and valid information on treatment outcome with either an SRI or an NRI were selected for genotyping.

Samples were genotyped on Illumina Human610-Quad BeadChips (*n* = 727) or Illumina Human660W-Quad BeadChips (*n* = 1,166), which have identical tag SNP coverage. More specifically, 727 samples from the GENDEP study were genotyped at the Centre National de Génotypage (Evry Cedex, France) using the Illumina Human610-Quad BeadChip and were subjects of previous reports [17],[18]. The GENPOD, GlaxoSmithKline, Pfizer, GODS, and 93 additional GENDEP samples were genotyped at the University of Geneva Medical School (Geneva, Switzerland) using the Illumina Human660W-Quad BeadChip for the NEWMEDS consortium.

### Quality Control

Quality control was implemented in PLINK [19], first on the level of the genetic marker and then on the level of the individual. Markers with minor allele frequency over 0.01 and at least 97% complete genotyping were retained. To avoid batch artefacts, markers that differed significantly (*p*<1×10^−3^) by genotyping centre were excluded. Overall, 520,978 (99.0%) of the 526,424 genotyped SNPs passed all stages of quality control and were included in pharmacogenetic analyses. Hardy–Weinberg equilibrium was tested, but was not used as an exclusion criterion for markers, since departures from Hardy–Weinberg equilibrium are expected in a case-only study [20].

Individuals were excluded for ambiguous sex (genotypic sex different from phenotypic sex) (*n* = 22), abnormal heterozygosity (*n* = 16), cryptic relatedness up to third-degree relatives by identity by descent (*n* = 20), genotyping completeness less than 97% (*n* = 9), and non-European ethnicity admixture detected as outliers in iterative EIGENSTRAT analyses of a linkage-disequilibrium-pruned dataset (*n* = 35). One additional individual was excluded because of invalid phenotypic information, leaving 1,790 (94.6%) of the 1,893 genotyped individuals for the pharmacogenetic analyses.

**Figure 1:**
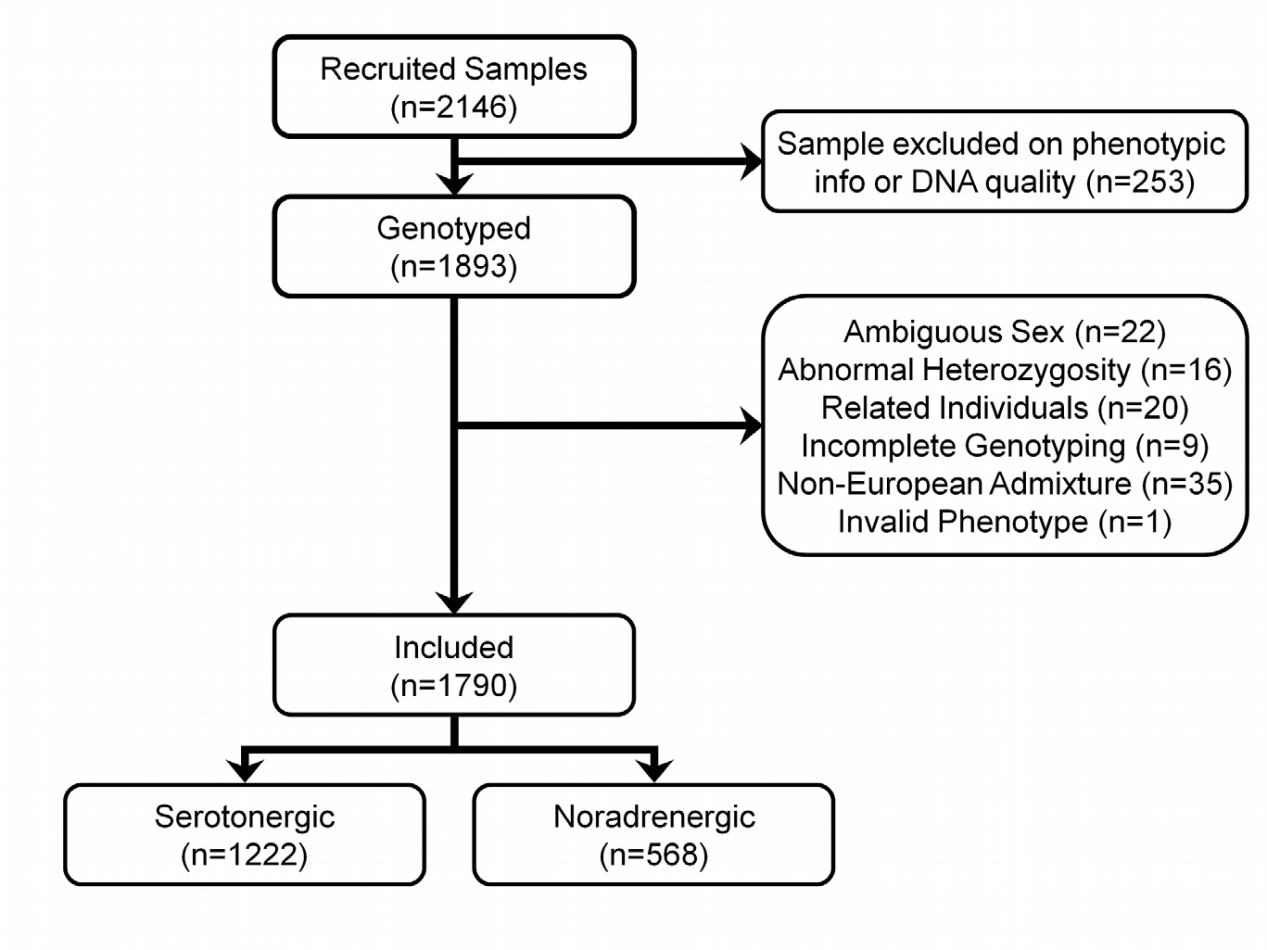
Flow of samples through quality control.

Figure 1 shows the flow of individuals through genotyping and quality control.

### Definition of Antidepressant Response Phenotype

Response to antidepressants involves changes in depressive symptoms over a number of weeks and is more accurately captured by continuous than by dichotomous variables [17],[21]–[23]. We defined response as a continuous variable, reflecting proportional reduction in depression severity from baseline to end of treatment. This measure is uncorrelated with initial severity (−0.10<*r*<0.10 in all component studies), is independent of depression rating scale used, and is clinically relevant since it closely reflects clinician's impression of improvement [17],[24].

Studies included in NEWMEDS used several outcome measures. The Montgomery–Åsberg Depression Rating Scale [25] was the primary outcome measure in GENDEP and GODS, the 17-item Hamilton Rating Scale for Depression (HRSD-17) [26] was the primary outcome measure in the studies conducted by Pfizer and GlaxoSmithKline, and the Beck Depression Inventory [27] was the primary outcome measure in GENPOD. While the outcome measures used differ in details, here we are interested in generalizable effects related to depression as a whole rather than effects specific to a particular measure. Previous research has shown no difference between classes of antidepressants in response as measured by these three scales [28]. In addition, we took the following steps to minimize the effects of scale differences. To allow for an unbiased analysis of the combined dataset, we converted the outcome measures within each study to a single continuous metric: a standardized change score, adjusted for sex, age, and recruitment centre within each contributing study. The adjusted change score was z-transformed within each study to remove any correlation between data origin and outcome prior to the genetic analysis, and to remove effects that are specific to individual contributing studies. Detailed information about the definition of the phenotype is provided in Text S1 (section 1.3).

### Statistical Analysis

Data analysis was carried out according to a protocol specified prior to data acquisition (Text S2). Joint analysis of individual-level data was conducted to allow for rigorous quality control and to retain maximum statistical power when combining studies that varied in size. The whole sample was analyzed jointly since this is a more efficient and powerful approach than discovery–replication design [29],[30]. Quality-controlled genotypes were tested for association with the adjusted percentage change in depression severity using linear regression under an additive genetic model in PLINK [19]. Four genome-wide analyses were performed. A first linear regression searched for common genetic markers that predict response to both types of antidepressants in the whole sample of 1,790 individuals. The second and third analyses tested predictors of response to SRIs (*n* = 1,222) and NRIs (*n* = 568). A fourth analysis, of primary interest to our second aim, searched the genome for common variants that differentially predict response to SRIs and NRIs. To avoid confounding by covariation between antidepressant drug and genetic background due to different studies contributing unequal numbers of individuals to each antidepressant group, we tested this hypothesis as a drug-by-genotype interaction in a sample restricted to individuals that were randomly allocated to treatment with either an SRI or an NRI (*n* = 949), thus ensuring full comparability of the two drug groups on measured and unmeasured confounders. In all analyses, the influence of genetic population structure was controlled by the inclusion of the four significant principal components from the final iteration of the EIGENSTRAT analysis of linkage-disequilibrium-pruned genetic data. We defined genome-wide statistical significance at the generally accepted threshold *p*<5×10^−8^ [31].

Associations reaching a less stringent threshold of *p*<5×10^−6^ are reported in Text S1 (section 2). Analyses performed by component study, summary data meta-analysis (Text S1, section 6), and pharmacogenetic associations within genes reported in previous candidate gene and genome-wide studies (Text S1, section 7) are also provided.

### Power Analysis

Our aim was to determine whether any common genetic variant predicts a clinically significant difference in the outcome of treatment with antidepressants, taking a difference of at least three points in the reduction of depression symptom severity on HRSD-17 as the benchmark for clinical significance [8]. Specifically, we aimed to achieve 80% power to detect an additive genetic effect that explains 6.33% of variance in outcome, corresponding to an HRSD-17 three-point difference in a drug comparison study [9]. Since not all common genetic variants were directly genotyped, we factored in imperfect tagging (at *R* ^2^ = 0.8) to estimate power for detecting effects of genotyped and ungenotyped variants. The quality-controlled sample provided a power well above 80% to detect a clinically significant effect at the genome-wide significance level for three of the four analyses (overall, SRI, and genotype–drug interaction). The meta-analysis of NEWMEDS and STAR*D samples had an adequate power to detect even an effect that was half of what would be considered clinically significant. Details of the power analysis can be found in Text S1 (section 1.4).

### Pathway Analysis

Enrichment of genome-wide association signal in genes that belong to known biological pathways was tested using ALIGATOR [32]. This method takes a predefined list of significant SNPs, and tests whether these SNPs cluster in genes belonging to a particular pathway more than would be expected by chance, allowing for varying numbers of SNPs per gene and non-independence of SNPs within and between genes. ALIGATOR corrects the significance levels of pathway-specific enrichment for the testing of multiple non-independent pathways. Further details of the pathway analysis can be found in Text S1 (section 3).

### Meta-Analysis with STAR*D

A meta-analysis was undertaken between NEWMEDS and data subsequently obtained from the first level of the STAR*D (Sequenced Treatment Alternatives to Relieve Depression) trial, when all participants with MDD were treated with citalopram (an SRI), in the hope of finding genome-wide significant associations. For further information about the STAR*D sample, genotyping, and quality control procedures see Text S1 (section 4.1).

To maximize the overlap between the two samples and genome coverage, both NEWMEDS and STAR*D were imputed to include over 1.4 million markers using BEAGLE 3.3 [33] and the HapMap phase 3 CEU population as the reference dataset. Meta-analysis was undertaken using the meta command in PLINK [19] in the entire samples and in a sample limited to individuals treated with a serotonergic antidepressant. More information about these methods used can be found in Text S1 (section 4).

### Polygenic Scoring

While both our main analysis in NEWMEDS and the meta-analysis with STAR*D had sufficient power to detect clinically significant genetic associations, there could also exist an underlying weak signal from across the genome that could offer insight into the mechanism of antidepressant response. The methodology of polygene scoring allows for the detection of such weakly distributed signal [34]. Details on this method can be found in Text S1 (section 5). Polygenic scores were created based on the NEWMEDS results and used to predict outcomes in STAR*D using linear regression. Two polygenic tests, one based on the entire NEWMEDS sample and the other restricted to SRI-treated participants, were carried out.

## Results

### Response to Any Antidepressant

**Figure 2:**
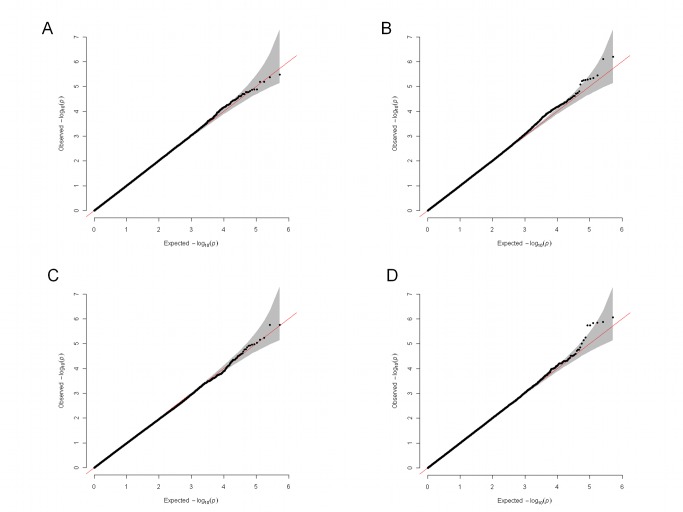
Quantile–quantile plots for the four genome-wide analyses. (A) Analysis of the whole sample (*n* = 1,790); (B) analysis of SRI-treated individuals (*n* = 1,222); (C) analysis of NRI-treated individuals (*n* = 568); (D) gene-by-drug interaction analysis in the randomly allocated individuals (*n* = 949). The shaded area is the 95% confidence interval of values expected under a uniform distribution.

**Figure 3:**
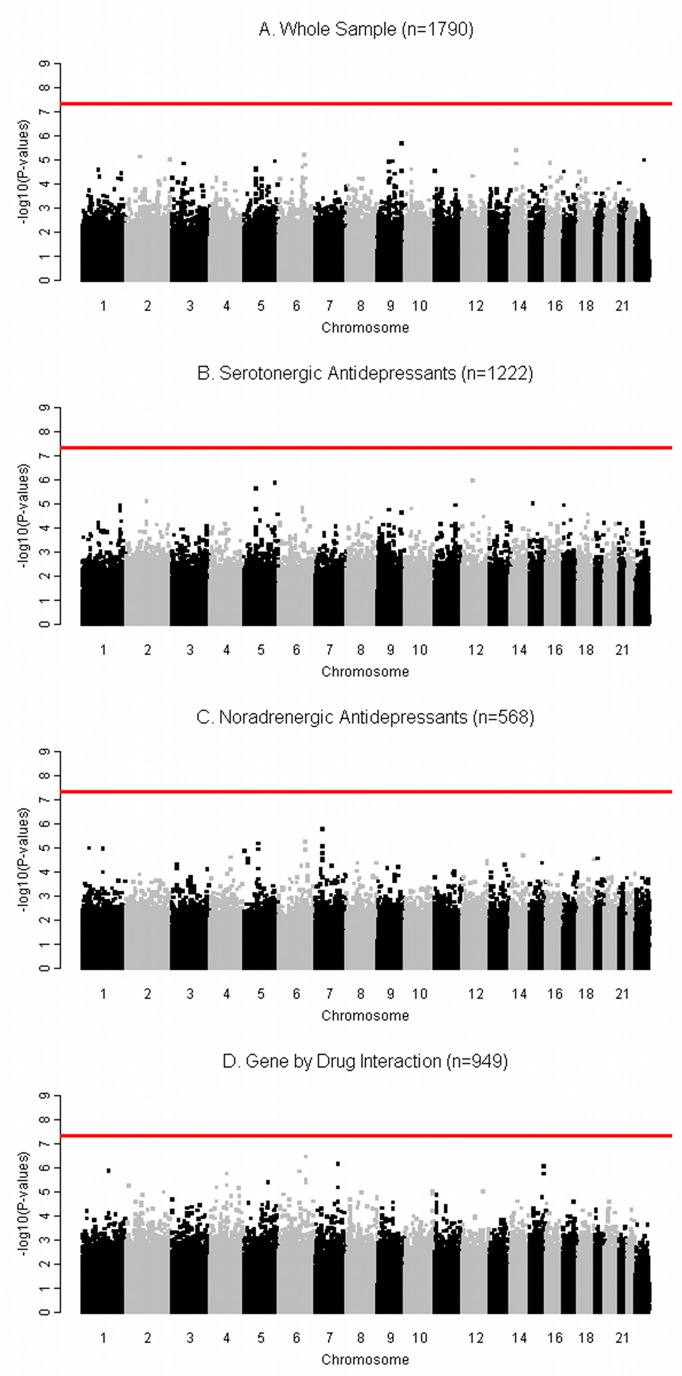
Manhattan plots for the genome-wide pharmacogenetic analyses showing results by −log10 *p*-value and chromosome location. The red line indicates the genome-wide significance level (*p*<5.0×10^−8^), and the absence of data points above this line indicates the lack of significant associations in the four analyses: (A) analysis of the whole sample (*n* = 1,790); (B) analysis of SRI-treated individuals (*n* = 1,222); (C) analysis of NRI-treated individuals (*n* = 568); (D) gene-by-drug interaction analysis in the randomly allocated individuals (*n* = 949).

Linear regression assessed the influence of 520,978 SNPs on the adjusted percentage change in depression severity in the whole sample of 1,790 antidepressant-treated individuals. A quantile–quantile plot showed a uniform distribution of *p*-values, with no inflation of the test statistic (median lambda = 1.0034; Figure 2). No association reached the genome-wide level of significance (Figure 3).

### Response to Serotonergic Antidepressants

A linear regression tested association between 520,978 SNPs and the adjusted percentage change in depression severity in 1,222 SRI-treated individuals. The quantile–quantile plot showed a uniform distribution of *p*-values, indicating no inflation of the test statistic (median lambda = 1.0094; Figure 2). No SNP was associated at the genome-wide level of significance (Figure 3).

### Response to Noradrenergic Antidepressants

A linear regression tested association between 520,978 SNPs and adjusted percentage change in depression severity in 568 NRI-treated individuals. The quantile–quantile plot showed a uniform distribution of *p*-values, with no inflation of the test statistic (median lambda = 0.9875; Figure 2). There were no significant associations (Figure 3).

### Differential Response to Serotonergic and Noradrenergic Antidepressants

A linear regression tested the interaction between 520,978 SNPs and antidepressant type (SRI versus NRI) in their effects on the adjusted percentage change in depression severity among the 949 individuals randomly allocated to SRI or NRI antidepressant. The quantile–quantile plot showed a uniform distribution of *p*-values, with no inflation of the test statistic (median lambda = 1.0015; Figure 2). No genotype–drug interactions were detected at the genome-wide level of significance (Figure 3).

For all four analyses, a meta-analysis of results from contributing studies also gave negative results (see Text S1, section 6).

### Pathway Analysis

Pathway analysis tested whether any biological pathways had more genes in the top 5% of genes (ranked by their most significant SNP) than expected by chance. None of the four analyses (response to any antidepressant, serotonergic antidepressants, noradrenergic antidepressants, and differential response to serotonergic and noradrenergic antidepressants) showed a significant excess in the number of enriched pathways, and no single pathway was significantly enriched after correcting for multiple testing. Full results are given in Text S1 (section 3.2).

### Meta-Analysis with STAR*D

A meta-analysis tested percentage improvement in 2,897 individuals from NEWMEDS and STAR*D using over 1.1 million genotyped and imputed SNPs and found no genome-wide significant results. A meta-analysis restricted to SRI-treated individuals (*n* = 2,329) found no genome-wide significant results. For more information see Text S1 (section 4.4).

### Polygenic Scoring

Polygenic scores were calculated to test the combined effect of multiple weak associations across the genome. Scores were created using NEWMEDS and used to predict outcomes in STAR*D. In the analysis that included all individuals (NEWMEDS, *n* = 1,790; STAR*D, *n* = 1,107), there was no significant prediction across the 13 progressive *p*-value thresholds. In a sample restricted to SRI-treated individuals (NEWMEDS, *n* = 1,222; STAR*D, *n* = 1,107), there was no significant prediction from any of the 13 progressive *p*-value thresholds either. Further information about the results can be found in Text S1 (section 5.2).

## Discussion

In a large pharmacogenetic analysis, including 1,790 antidepressant-treated individuals with MDD, none of the more than 500,000 genetic markers predicted treatment outcome after genome-wide correction. Since our study had adequate statistical power to detect common genetic variants with a clinically significant predictive effect, the results suggest that single marker prediction will not contribute to personalizing prescription of currently available antidepressants. Increasing sample size may aid in obtaining positive results in future studies, which may provide insight into the mechanism of the therapeutic action of antidepressants, even though the effect size will likely be too small to translate into clinical applications.

The lack of genome-wide significant or even moderately strong associations among a comprehensive list of candidate genes (details in Text S1, section 7) puts previously reported positive results from smaller candidate gene studies [4],[35] into a sobering perspective. The current study also fails to strengthen associations found previously in the genome-wide association study of the GENDEP sample [17], the largest sub-sample in the NEWMEDS consortium, or other genome-wide pharmacogenetic studies [11],[12],[17]. Furthermore, the current investigation fails to find a genome-wide significant association in the largest pharmacogenetic meta-analysis to date, which included 2,897 individuals. Pathway analysis has not shown any enrichment for a known biological pathway. Polygenic prediction has not found any evidence for a distributed convergent signal between the two largest pharmacogenetic samples collected to date. It is therefore possible that common polymorphisms will not help predict the outcome of treatment with commonly used antidepressants in a clinical meaningful way.

### Limitations

The present study benefited from a large sample through the combination of participants from multiple studies. This limits the interpretation of the present results in several ways. One limitation is the use of multiple antidepressants, each differing slightly in its chemical structure, transporter affinity, and receptor binding profile. Based on previous pharmacogenetic data [14], we hypothesized the existence of common genetic predictors of response to antidepressants with broadly defined modes of action, such as serotonin and noradrenaline reuptake inhibition. While we found no predictors of response to SRI or NRI types of antidepressants, our results are compatible with the existence of pharmacogenetic effects that are specific to a particular antidepressant compound. Large groups treated with the same drug may uncover genetic predictors that were not detected in the present study. However, the largest existing sample treated with the same antidepressant has also failed to detect significant genetic predictors in a genome-wide analysis [11], and it is unlikely that even larger samples with homogeneous treatment will be collected in the near future. The studies included in NEWMEDS also differed in other aspects, e.g., in the depression rating scale used and in the way participants were recruited. The adjusted change score used as the phenotype in this study removed effects specific to each individual contributing study. This measure was intended to minimize the risk of spurious findings, but it could have reduced the impact of a genuinely larger treatment effect in a particular study. However, our aim was to detect pragmatic predictors that generalize to multiple settings rather than effects specific to a particular homogeneous group. It is unlikely that pharmacogenetic predictions limited to a particular depression rating scale or to a more homogeneous subgroup of patients would be clinically meaningful or commercially viable. Furthermore, a meta-analysis conducted across the individual studies provided similar results (see Text S1, section 6). A related limitation was the smaller size of the NRI-treated sample, meaning that only relatively strong predictors could be identified in this arm of the study. Additional limitations pertain to the scope of the present study. Our results are limited to the influence of common polymorphisms on the therapeutic effects of several monoaminergic antidepressants under study in individuals of European ancestry. Studies in other populations and studies of antidepressants with a non-monoaminergic mode of action are needed to extend the scope of pharmacogenetic exploration.

### Conclusions and Future Directions

Our study adds to the growing literature of genome-wide pharmacogenetic studies, offering, to our knowledge, the largest body of pharmacogenetic data available to date. The absence of pharmacogenetic associations with clinically meaningful effect suggests that common genetic variation is not ready to inform personalization of treatment for depression. Future studies may need to combine clinical, genetic, epigenetic, transcriptomic, and proteomic information to obtain clinically meaningful prediction of how an individual with major depression will respond to antidepressant treatment.

## Supporting Information

Text S1 **Supplementary methods and results.** Supplementary text, tables, and figures including further information about the individual studies included in the analysis, suggestive (*p*<5×10^−6^) results from whole genome analyses in NEWMEDS, pathway analysis, meta-analysis between NEWMEDS and STAR*D, polygenic score, study-level meta-analysis between samples in NEWMEDS, reporting of results for previously identified candidate genes for antidepressant response, and imputation results from NEWMEDS.
(PDF)

Text S2 **Study protocol.**
(PDF)

## Abbreviations

HRSD-17: 17-item Hamilton Rating Scale for Depression
MDD: major depressive disorder
NRI: noradrenaline reuptake inhibitor
SRI: serotonin reuptake inhibitor
