# Supplementary material for: Genetic Predictors of Response to Serotonergic and Noradrenergic Antidepressants in Major Depressive Disorder: A Genome-Wide Analysis of Individual-Level Data and a Meta-Analysis
Source: PLoS Med. 2012 Oct 16;9(10):e1001326. doi: 10.1371/journal.pmed.1001326 (PMC3472989; doi:10.1371/journal.pmed.1001326)
Supplement: Text S1 — Supplementary methods and results. Supplementary text, tables, and figures including further information about the individual studies included in the analysis, suggestive (p<5×10^−6^) results from whole genome analyses in NEWMEDS, pathway analysis, meta-analysis between NEWMEDS and STAR*D, polygenic score, study-level meta-analysis between samples in NEWMEDS, reporting of results for previously identified candidate genes for antidepressant response, and imputation results from NEWMEDS. (PDF) [file pmed.1001326.s001.pdf]

Genetic Predictors of Response to Serotonergic and Noradrenergic Antidepressants in Major Depressive Disorder: a NEWMEDS consortium report

Supplementary Materials

Table of Contents:

1 NEWMEDS materials and methods 2
1.1 Individual Sample Information 2
1.1.1 GENDEP 2
1.1.2 GenPod 3
1.1.3 GODS 4
1.1.4 Pfizer 4
1.1.5 Glaxo Smith Kline 5
1.2 Quality control 7
1.3 Definition of antidepressant response phenotype 8
1.4 Power analysis 10
2 Suggestive results from the whole genome analyses in NEWMEDS 11
2.1 Response to any antidepressant 11
2.2 Response to Serotonergic Antidepressants 11
2.3 Response to Noradrenergic Antidepressants 12
2.4 Differential response to serotonergic and noradrenergic antidepressants 13
3 Pathway Analysis in NEWMEDS 15
3.1 Methods 15
3.2 Results 16
4 Meta-analysis with STAR*D 17
4.1 Materials and Methods 17
4.1.1 STAR*D 17
4.2 Imputation 19
4.3 Analysis 20
4.4 Results 20
4.4.1 Any Antidepressant 20
4.4.2 Serotonergic Antidepressants 22
5 Polygene scoring 24
5.1 Method 24
5.2 Results 25
5.2.1 Any Antidepressant 25
5.2.2 Serotonergic Antidepressant 25
6 Study level meta-analysis between the samples in NEWMEDS 26
6.1 Individual study results (QQ plots) 26
6.2 Study level meta-analysis method 29
6.3 Results 29
6.3.1 Study level meta-analysis of response to any antidepressant (n=1790) 29
6.3.2 Study level meta-analysis of response to SRIs (n=1222) 34
6.3.3 Study level meta-analysis of response to NRIs (n=568) 38
7 Candidate gene analysis in NEWMEDS 42
7.1 Exploration of candidate genes 42
7.2 Results 42
8 Imputation results from NEWMEDS 58
9 References: 67

1 NEWMEDS materials and methods

1.1 Individual Sample Information

1.1.1 GENDEP

A detailed description of the Genome-Based Therapeutic Drugs for Depression (GENDEP) sample and design is available elsewhere [1]. Briefly, GENDEP was a twelve-week open-label part-randomized multi-centre study with two active pharmacological treatment arms [1]. This includes 57 additionally recruited participants which were not subjects to previous reports. This enlarged GENDEP sample includes 868 treatment-seeking adults (men n=321; women n=547) diagnosed with ICD-10/DSM-IV unipolar major depression of at least moderate severity established in the Schedules for Clinical Assessment in Neuropsychiatry (SCAN) interview [2]. Severity of depression was assessed weekly by three established rating scales [3]. Personal or family history of bipolar disorder or schizophrenia and active substance dependence constituted exclusion criteria. Individuals were of white European origin and ranged from 19 to 72 years of age with a mean age of 42.6 years (S.D=11.7). Eligible participants were treated with either escitalopram or nortriptyline. These two drugs represent different mechanisms of antidepressant action with escitalopram primarily affecting serotonergic neurotransmission and nortriptyline primarily affecting noradrenergic neurotransmission [4,5]. Patients with no contraindications were randomly allocated to flexible-dosage nortriptyline (50–150 mg daily) or escitalopram (10–30 mg daily) for 12 weeks. Patients with contraindications for one drug were offered the other. A total of 628 participants (77%) completed at least 8 weeks of treatment with the originally allocated antidepressant [1]. Individuals were excluded from the analysis if they had no post baseline information. The study was approved by ethics boards in all participating

centres. All participants provided a written consent after the procedures were explained. GENDEP is registered at EudraCT (No.2004-001723-38, http://eudract.emea.europa.eu) and ISRCTN (No. 03693000, http://www.controlled-trials.com).

1.1.2 GenPod

A full description of the methodology and sample of the GENetic and clinical Predictors Of treatment response in Depression (GenPod) study can be found elsewhere [6]. The study was a multi-centre randomized clinical trial of 601 patients with depression (men n=161 women n=347) recruited in primary care who had an ICD-10 diagnosis of major depression of at least moderate severity as assessed by the Clinical Interview Schedule-Revised (CIS-R) [7] and the Beck Depression Inventory (BDI) [8]. Individuals were randomly allocated to either reboxetine (4mg twice daily) or citalopram (20mg). These two drugs represent different mechanisms of antidepressant action with citalopram primarily affecting serotonergic neurotransmission and reboxetine primarily affecting noradrenergic neurotransmission. As ethnicity is a major confounder in genetic studies due to the introduction of LD and haplotypic difference across ethnic backgrounds, only individuals with a white European ancestry were chosen for the whole genome analysis (n=512). Individuals were aged between 18-74 years with a mean age of 38.8 years. Exclusion criteria included if individuals had psychosis, bipolar disorder or major substance or alcohol abuse, or if they had medical contraindications. Individuals were further excluded from the analysis if they had no post baseline information. All participants provided written consent after the study and procedure were explained. Ethical approval was obtained from the South West Ethics Committee (MREC 02/6/076) as well as research governance approval from Bristol,

Manchester and Newcastle Primary Care NHS Trusts. The ISRCTN is 31345163 and EudraCT number 2004-001434-16.

1.1.3 GODS

The Geneva Outpatient Depression Study (GODS) has been described in detail elsewhere [9,10]. Briefly, GODS is a partly randomized study, which examined the efficacy of four antidepressants (paroxetine, clomipramine, venlafaxine and nefazodone) based on a seven-step algorithm in a cohort of 131 subjects (53 men and 78 women) with severe MDD [11] aged 18 to 65 years. Exclusion criteria included pregnancy, schizophrenia or schizoaffective disorder, dependence on alcohol or other substances and treatment with mood-stabilisers or antipsychotics. The present investigation includes data from the first three steps that included treatment with paroxetine (an SRI), initiated at 20mg daily and increased to 30mg and 40mg daily if remission was not achieved. Patients were discharged only if complete remission was obtained as defined by a MADRS score of 8 or less. Of the 131 GODS participants, 82 had available blood DNA samples and reported white European ancestry. The study protocol was approved by the ethics committee of the Geneva University Department of Psychiatry and written informed consent was obtained from all subjects.

1.1.4 Pfizer

A total of 345 patients from eight MDD clinical trials were provided. Study designs were variable and primarily conducted as double-blind, placebo-controlled, 6 to 8 weeks studies with sertraline, fluoxetine or paroxetine as active comparators in addition to the investigational compound. Only subjects from the SRI comparator arms were sent for whole genome genotyping and included in the current study. All study protocols received institutional review board (IRB) approval and informed

consent was obtained from participating subjects prior to sample collection. In all eight trials, a diagnosis and inclusion criterion for MDD was a Hamilton Depression Rating Scale (HAM-D) total score of 22 or higher at screening. Exclusion criteria included DSM-IV diagnosis of psychotic features, bipolar I or II, or major risk for suicide.

1.1.5 Glaxo Smith Kline

The samples from GSK derived from two randomized, double-blind, placebo-controlled comparisons of the antidepressant efficacy and the effects on sexual functioning of Bupropion XL and escitalopram in outpatients with moderate to severe depression [12]. The studies were parallel groups and identically designed, conducted between January 2003 and June 2004 in the United States. Escitalopram or matching placebo capsule was administered at doses of 10mg/day for the first 4 weeks and either 10mg/day, or if clinically indicated, 20mg/day from Week 5 through Week 8. Included subjects had primary diagnosis of Major Depressive Disorder (MDD) with duration at recruitment lasting 12 weeks but no greater than 2 years, and having failed to respond to two adequate trials of antidepressants in the previous 2 years. The primary outcomes for depression was the change from baseline in HAMD-17 total score, whilst the secondary outcomes were percent of subjects in remission and percent of responder (HAMD-17), plus CGI-I and CGI-S. Out of the 210 patients in the escitolapram treatment arms who completed the study, 137 were selected based on availability of consented DNA blood sample and white Caucasian ethnicity; with an average age of 36.4 (from 18 to 64) and 45:55 male to female ratio. All patients provided written informed consent prior to any study activity and the protocol for each of the studies was approved by international review boards.

Table 1: Description of the five component studies.

Study	GENDEP	GenPod	GODS	Pfizer	Glaxo-Smith Kline
N	868	601	131	355	191
% Female	63.0%	57.7%	59.5%	67.3%	57.1%
Age<br>(average in years)	42.6 (SD 11.7)	38.8 (SD 12.4)	36.5 (SD 10.6)	43.3 (S.D. 13.2)	35.9 (SD 11.3)
Severity	Moderate to Severe	Moderate to Severe	Severe	Moderate to Severe	Moderate to Severe
Baseline HRSD-17	21.8 (SD 5.3)			23.6 (S.D. 3.4)	23.7 (SD 3.8)
Baseline MADRS	28.8 (SD 6.8)		33.2 (SD 5.2)		
Baseline BDI	28.1 (SD 9.7)	33.5 (SD 9.7)			
SRI	escitalopram	citalopram	paroxetine	sertraline,<br>fluoxetine,<br>paroxetine	escitalopram
NRI	nortriptyline	reboxetine	-	-	-
Exclusion Criteria	Personal or family history of bipolar disorder or schizophrenia;<br>active substance dependence	Personal history of bipolar disorder or psychosis;<br>Major substance or alcohol abuse;<br>Medical contraindications	Personal history of schizophrenia or schizoaffective disorder;<br>Dependence on alcohol or other substances;<br>Pregnancy;<br>Treatment with mood stabilizers Or antipsychotics	Personal history of bipolar disorder<br>(I or II),<br>psychotic features;<br>high risk of suicide	Personal history of bipolar disorder<br>(I or II),<br>psychotic features;<br>high risk of suicide

1.2 Quality control

All quality control procedures were undertaken using PLINK [13]. Quality control was first implemented on the marker level than on an individual level.

Individuals were excluded for ambiguous sex (genotypic sex different from phenotypic sex) (n=22) and abnormal heterozygosity on autosomes (n=16). Cryptic relatedness was assessed through identity by descent (IBD) using PLINK [13] to a linkage disequilibrium (LD) pruned dataset. IBD was assessed both within and between studies. Individuals were excluded if they were first, second or third degree relatives (n=20). Genotyping completeness was examined and individuals excluded if completeness was less than 97.5%. Most individuals were complete for all genotyping following removal of poor calling SNPs (n=9).

Markers were excluded if they had a minor allele frequency of less than 0.01 as effects of rare markers on response to antidepressants is not in the scope of the current analysis and the sample is not powered to detect associations with markers below 0.01. Markers were assessed for completeness and markers less than 97% complete were excluded. Hardy-Weinberg equilibrium (HWE) was tested, but was not used as a criterion for exclusion of markers. Since the study is a case only analysis, there may be departures from HWE [14]. HWE was examined for all significantly associated SNPs and is reported in the results.

Sample selection from studies was limited to individuals of white European parentage. In order to ensure this was correct and to check self-reported ethnicity with genetic ethnicity as well as to correct to genetic architecture differences within European populations [15], we performed a principal component analysis using EIGENSTRAT [16]. An LD pruned dataset was used to remove confounding by local LD. The LD pruned dataset contained 30298 SNPs in low LD and excluded known

region of long-range LD [16]. A single EIGENSTRAT analysis was conducted with samples from all studies and run iteratively. The first iteration was run with five HapMap populations (CEU, YRI, CHB, JPT and GIH) and 35 individuals were excluded as outliers and removed from further analyses as analysis indicated they had strong African or Indian admixtures. After their exclusion, a second iteration was performed on just sample. No outliers were detected and the first four principal components were nominally significant (Tracy-Widom $p<0.05$), and these were used as covariates in the genetic association analyses.

1.3 Definition of antidepressant response phenotype

Response to antidepressants is a complex phenotype that involves changes in depressive symptoms over a number of weeks and best assessed by repeated administration of depression rating scales such as the Hamilton Rating Scale for Depression (HRSD-17) [17], Montgomery and Asberg depression rating scale (MADRS) [18] or Beck Depression Inventory (BDI) [8]. Since response to antidepressants is a matter of degree rather than a dichotomous yes-or-no, we defined the outcome as a continuous variable, reflecting the proportional reduction of depression severity from baseline to study exit [19-22].

Studies included in NEWMEDS used different scales as primary outcome measures. MADRS was the primary outcome measure in GENDEP and GODS, HRSD-17 was the primary outcome measure in the studies conducted by Pfizer and GSK, and BDI was the primary outcome measure in GenPod. To allow an unbiased analysis of the combined dataset, we converted the outcome measures within each study to a single continuous metric: a standardized change score, adjusted for sex, age and recruitment centre in mutli-centre studies within each contributing study. First, a change score was calculated as percentage reduction in depression severity over 12 weeks of

antidepressant treatment, with missing values at study exit imputed based on earlier measurements, using mixed effect linear models, as previously described [23]. In agreement with our previous study [22], we chose percentage change because it is uncorrelated with initial severity, relatively independent of which scale is used, and closely reflects clinician's impression of improvement [24]. Imputation of missing end-point data based on mixed effect models minimized the biases inherent in procedures such as last-estimation carried forward [23,25,26]. Second, we adjusted the percentage change for sex, age and recruitment centre in multi-centre studies within each study, to avoid results being confounded through these variables. Third, to avoid confounding by data origin, the adjusted change score was z-transformed within each study (i.e. linearly converted to a variable with a mean of zero and a standard deviation of 1). This final step removes any correlation between data origin and outcome prior to the genetic analysis.

1.4 Power analysis

Our aim was to determine if any common genetic variant predicts a clinically significant difference in the outcome of treatment with antidepressants. Clinical significance was previously defined as a difference of at least 3 points in the reduction of depression symptoms severity on HRSD-17 [27,28]. We aimed to achieve 80% power to detect an additive genetic effect that explains 6.33% of variance in outcome, corresponding to a 3 HRSD-17 point difference in a drug comparison study [27]. .Power analysis was conducted using the Genetic Power Calculator [29]. We carried out both individual-level analyses and meta-analyses. Since the power for fixed effects meta-analysis and individual-level data are identical, we only present one set of power calculations [30]. We factored in imperfect tagging (at $R^2 = 0.8$) to estimate power for detecting effects of genotyped or ungenotyped variants. This assumption is in fact conservative: a report examining this issue has found that the coverage of the Illumina 610/660 chip is 87%, and across a range of effect sizes the power for finding association with genotyped variants drops by less than <10% (and often <5%) compared to complete genome-wide genotyping [31].

Three of the four analysis had well over 80% power to detect a clinically significant variant at the genome wide significance level ($p<5*10^{-8}$) with imperfect tagging of $R^2 = 0.8$ (overall, SRI, and genotype-drug interaction). The fourth analysis (NRI) had only 27% power to detect a clinically significant variant at the genome wide significance level. In the meta-analysis with STAR*D, both analyses had adequate power (greater than 80%) to detect a clinically significant variant at the genome wide significance level.

We further investigated if our samples had the power to detect an effect explaining half the clinical significant (3.165%). The meta-analysis of NEWMEDS and STAR*D

had adequate power (greater than 80%) to detect a variant of this size at the genome wide significance level.

2 Suggestive results from the whole genome analyses in NEWMEDS

2.1 Response to any antidepressant

Two SNPs were associated with outcome at the suggestive level of significance (rs10818702 $p=2.19*10^{-6}$; rs11624702 $p=4.08*10^{-6}$). rs10818702 is located on chromosome 9 in an intron of *OR1J2* (olfactory receptor, family 1, subfamily J). OR1J2 is a G-protein-coupled receptor, also expressed in the amygdala and cerebellum.

rs11624702 is located on chromosome 14 in an intron of *MDGA2* (MAM domain containing glycosylphosphatidylinositol anchor 2), a novel member of the adhesion molecules of the immunoglobulin superfamily involved in cell adhesion, migration, and recruitment to inflammatory sites [32]. *MDGA2*, formerly *MAMDC1*, has been associated with neuroticism and neuroticism-related phenotypes in a whole genome and targeted replicated studies [33-35]. Neuroticisim, a personality trait reflecting a tendency towards negative mood states, is strongly related to depression and shares genetic predisposition with depressive phenotypes [36,37].

2.2 Response to Serotonergic Antidepressants

Four SNPs were associated with outcome at the suggestive level ($p<5*10^{-6}$). The strongest associated SNP was rs10783282 ($p=1.16*10^{-6}$), located on chromosome 12 within an intron of *LOC255411*, a validated non-coding RNA (miscRNA), and 13kb upstream of *ADCY6*, adenylate cyclase 6 isoform A, a membrane-associated enzyme which catalyzes the formation of the secondary messenger cyclic adenosine monophosphate (cAMP), and is implicated in sleep/wake cycle regulation [38,39].

ADCY6 expression is modulated by *miR-182* [38], and variation within pre-cursor *miR-182*, is associated with late insomnia in individuals with MDD [39]. Additionally, individuals with depression have been reported to have reduced platelet ADCY activity [40,41] and a gene in the ADCY family was associated with depression in a genome wide study [40].

Two associated SNPs were on chromosome 5 within *ADAMTS6* (rs1493451 $p=2.35*10^{-6}$; rs7708972 $p=2.49*10^{-6}$). These SNPs are in high LD ($r^2=0.84$) making a single association signal. *ADAMTS6* is a disintegrin and metalloproteinase with thrombospondin motifs, and member of a family of proteins implicated in the turnover of the extracellular matrix [42]. *ADAMTS6* transcription is regulated by the pro-inflammatory cytokine TNFα [42], suggesting a link between inflammatory regulation and the action of antidepressants.

The remaining associated SNP (rs10515893 $p=1.37*10^{-6}$) was in an intergenic region on chromosome 5, 1.8Mb away from the nearest gene.

2.3 Response to Noradrenergic Antidepressants

Two SNPs located within *HIBADH* were the top results (rs13237776 $p=1.76*10^{-6}$; rs12534474 $p=1.76*10^{-6}$). These two SNPs are in complete LD ($r^2=1.0$) indicating a single association signal. *HIBADH* encodes for a 3-hydroxyisobutyrate dehydrogenase, a dimeric mitochondrial enzyme that catalyzes the NAD dependent reversible oxidation of 3-hydroxyisobutrate. Another SNP located on chromosome 6 11kb upstream of *ARHGAP18* was associated with improvement during treatment with noradrenergic antidepressants just below the suggestive level of evidence ($p=5.83*10^{-6}$). *ARHGAP18* is part of the human RhoGAP family of GTPase-activating proteins, which are important in neuronal development and plasticity.

2.4 Differential response to serotonergic and noradrenergic antidepressants

Two of these SNPs were on chromosome 15: rs2279447 ($p=8.87*10^{-7}$) and rs1455773 ($p=1.84*10^{-6}$). rs2279447 is located 1.6kb upstream of *ST8SIA2* (ST8 alpha-N-acetyl-neuraminide alpha-2,8-sialyltransferase, encoding a type II membrane protein) and 1.3kb upstream from hypothetical protein *C15orf32*. rs1455773 is located within the first exon of the hypothetical protein *C15orf32* and is a missense mutation (Alanine to Threonine change). Two further associated SNPs are on chromosome 1 within introns of a hypothetical non-coding RNA, *LOC400794* (rs1409414 $p=1.33*10^{-6}$; rs7549782 $p=1.44*10^{-6}$), and 126kb upstream of *MGST3* (microsomal glutathione S-transferase 3), a human-specific intestinal drug metabolizing enzyme. The remaining two associated SNPs (rs280060 $p=1.82*10^{-6}$; rs4424090 $p=1.48*10^{-6}$) were in intergenic regions on chromosome 4 and 6, more than 100kb away from the nearest known genes.

Two markers of differential response to SRI and NRI are located downstream of *ST8SIA2*, which is highly expressed throughout the brain (Allen Brain Atlas, http://www.brain-map.org/) and may modulate neural cell adhesion (NCAM) [43]. *ST8SIA2*-mediated polysialylation of NCAM has been implicated in the etiology of MDD with NCAM knockout mice expressing behavioral symptoms of depression which are reversible with antidepressant treatment [44] and in the action of psychotropic drugs, including the mood-stabilizer valproic acid [45]. Moreover, *ST8SIA2* is located under a linkage peak for recurrent early onset MDD [46] and *ST8SIA2* variants have been associated with risk for schizophrenia and bipolar disorder [47-49].

Table 2: Top results from the four genome-wide analyses. Markers shown in bold italics had the lowest p-value for that analysis. CHR=Chromosome; HWE=Hardy-Weinberg Equilibrium; MAF=Minor Allele Frequency. Regression coefficient is standardized and can be interpreted as a measure of effect size: it is the number of standard deviations in outcome per minor allele. Positive values of regression coefficient mean that carriers of more minor alleles had better treatment outcome. Negative values of regression coefficient mean that carriers of more minor alleles had worse outcomes.

						Linear Regression Analyses									
						Whole Sample (n=1790)		Serotonergic Only (n=1222)		Noradrenergic Only (n=568)		Gene by Drug Interaction(n=949)			
Phenotype	CHR	SNP	Position	Nearest Gene	Allele	Regression Coefficient	P-Value	Regression Coefficient	P-Value	Regression Coefficient	P-Value	Regression Coefficient	P-Value	HWE	MAF
Whole Sample Analysis (n=1790)	9	rs10818702	124285545	*OR1J2*	A	***-0.19***	***2.19E-06***	-0.21	2.42E-05	-0.14	0.04	0.12	0.27	0.94	0.22
	14	rs11624702	46588187	*MDGA2*	A	***0.15***	***4.08E-06***	0.14	3.32E-04	0.16	3.83E-03	0.002	0.98	0.48	0.47
Serotonergic Analysis (n=1222)	5	rs7708972	64724497	*ADAMTS6*	G	-0.14	2.37E-05	***-0.19***	***2.49E-06***	-0.03	0.61	0.16	0.08	0.25	0.49
	5	rs1493451	64762196	*ADAMTS6*	G	-0.13	9.20E-05	***-0.19***	***2.35E-06***	-5.47E-04	0.99	0.2	0.03	0.65	0.46
	5	rs10515893	164692074		A	-0.16	1.21E-05	***-0.21***	***1.37E-06***	-0.05	0.44	0.17	0.1	0.2	0.27
	12	rs10783282	47433214	*ADCY6*	A	-0.16	4.96E-05	***-0.24***	***1.16E-06***	7.73E-05	0.99	0.19	0.08	0.1	0.22
Noradrenergic Analysis (n=568)	7	rs13237776	27676198	*HIBADH*	A	-0.12	2.94E-03	-0.01	0.79	***-0.34***	***1.77E-06***	-0.24	0.03	0.7	0.19
	7	rs12534474	27677080	*HIBADH*	A	-0.12	2.78E-03	-0.01	0.77	***-0.34***	***1.77E-06***	-0.24	0.03	0.7	0.2
Gene by Drug Interaction (n=949)	1	rs1409414	163736265	*RXRG*	A	0.02	0.74	-0.12	0.09	0.3	2.62E-03	***0.72***	***1.33E-06***	0.72	0.09
	1	rs7549782	163740216	*RXRG*	A	0.02	0.77	-0.12	0.08	0.3	2.41E-03	***0.72***	***1.44E-06***	0.72	0.09
	4	rs280060	95177048		C	0.04	0.23	0.14	3.92E-04	0.15	7.23E-04	***-0.42***	***1.82E-06***	0.44	0.49
	6	rs4424090	99253795		A	-0.14	0.12	0.03	0.78	-0.61	5.56E-04	***-1.29***	***1.48E-06***	1	0.03
	15	rs2279447	90814564	*ST8SIA2 C15orf32*	G	-0.02	0.54	0.06	0.15	-0.18	2.63E-03	***-0.46***	***8.86E-07***	0.41	0.32
	15	rs1455773	90816431	*ST8SIA2 C15orf32*	A	-0.01	0.78	0.07	0.09	-0.17	5.64E-03	***-0.45***	***1.84E-06***	0.71	0.32

3 Pathway Analysis in NEWMEDS

3.1 Methods

The gene sets used in our pathway analyses came from five publicly-available sources: 1) Gene Ontology (GO) [50], accessed on November 8th 2011, 2)KEGG (Kyoto Encyclopedia of Genes and Genomes pathways; ftp://ftp.genome.jp/pub/kegg/genes/organisms/hsa/hsa_pathway.list) accessed on June 27th 2011 3)Mouse Genome Informatics (MGI) database [51], accessed on February 22nd, 2010, 4)PANTHER (Protein Analysis THrough Evolutionary Relationships) [52], accessed on August 20th 2010, and 5) the "canonical pathways" collection from the Molecular Signatures Database (MSigDB) [53], accessed on February 2nd 2011. Gene sets were required to contain between 3 and 300 genes to be included in the analysis, giving a total of 14,518 gene sets. A large collection of gene sets was used to maximise the chance of at least one gene set corresponding to the (unknown) disease biology.

Pathway analysis of the GWAS data was carried out using ALIGATOR (Association LIst Go AnnoTatOR), as described in Holmans et al. [54], using the gene sets described above. ALIGATOR converts a list of significant and nominally significant SNPs into a list of significant genes, and tests this list for enrichment within defined categories. ALIGATOR corrects for variable numbers of SNPs per gene and variable gene size. This allows us to obtain p-values for enrichment for each gene set, correct these for testing multiple non-independent gene sets, and to test whether the number of significantly enriched gene sets is higher than expected. Gene sets required at least two signals to be counted as enriched to remove the possibility of a small gene set

being deemed significantly enriched based on one signal. An important modification to the original ALIGATOR method is that significant genes in the same gene set that mapped less than 1Mb apart (and thus could be explained by the same association signal) were counted as a single signal. SNPs that mapped within the boundaries of a gene (genome build 36_3) were assigned to that gene: if SNPs mapped within more than one gene, they were assigned to all such genes. 224,475 SNPs were assigned to 16,976 genes by this method.

Following Stergiakouli et al. [55], the list of significant genes was chosen to encompass the top 5% of all genes covered by SNPs, a total of 848 genes. This corresponded to a p-value criterion of approximately 0.007 (varying slightly between phenotypes) for defining significant SNPs (with between 3,377 and 3,762 SNPs so defined – see Table 3).

3.2 Results

The numbers of pathways enriched at various levels of significance (p=0.05, p=0.01, p=0.001) are given in Table 3, together with a p-value from a test of whether the number of pathways reaching each level of enrichment was significantly greater than would be expected by chance. A greater than expected number of significantly enriched pathways would indicate the presence of underlying disease biology tagged by the pathways tested. However, it can be seen from Table 3 that none of the phenotypes yielded a significant excess of enriched pathways at any of the significance levels of enrichment. Furthermore, no pathway yielded a pathway-specific p-value for enrichment that was sufficiently significant to withstand correction for multiple testing of pathways.

Table 3: Numbers of significantly-enriched pathways from ALIGATOR analyses of GWAS data, together with p-values testing whether the number of enriched pathways is higher than expected by chance.

Phenotype	p-value criterion	Number of top SNPs	p<0.05		p<0.01		p<0.001	
			Number of pathways	p-value	Number of pathways	p-value	Number of pathways	p-value
Gene by Drug Interaction (n=949)	0.0068	3530	392	0.108	79	0.155	11	0.182
Whole Sample Analysis (n=1790)	0.0065	3615	391	0.126	77	0.182	10	0.240
Noradrenergic Analysis (n=568)	0.0073	3377	298	0.475	54	0.540	5	0.630
Serotonergic Analysis (n=1222)	0.0068	3762	397	0.113	95	0.073	13	0.120

4 Meta-analysis with STAR*D

Meta-analysis for other psychiatric disorders, such as biopolar disorder [56] and schizophrenia [57], have successfully discovered genome wide significant associations when preliminary sample analyses were moderately successful. We attempt a meta-analysis between NEWMEDS and STAR*D to see if increasing the power through increased sample size would aid in finding a genome wide significant association.

4.1 Materials and Methods

4.1.1 STAR*D

4.1.1.1 Sample

The Sequenced Treatment Alternatives to Relieve Depression (STAR*D) study has been described in detail elsewhere [58,59]. Briefly, STAR*D included 4041 treatment-seeking adult outpatients (18-75 years) with a diagnosis of non-psychotic unipolar major depressive disorder. Treatment-seeking Individuals were recruited in 18 primary care and 23 psychiatric clinical sites across the United States [58] and had a minimal depression severity of 14 on Hamilton Rating Scale for Depression

(HRSD-17) [17]. For the replication and meta-analysis presented here, only data from the first treatment step, protocol-guided citalopram (an SRI) 20 to 60mg daily was included [59]. STAR*D was approved by institutional ethics review boards in all centres. All participants provided a written consent after the procedures and any associated risks were explained.

4.1.1.2 Genotyping and Quality Control

Genetic material was collected from 1,948 individuals and genotyped on the Human Mapping 500K Array Set (n=969) or Affymetrix Genome-Wide SNP Array 5.0 (Affymetrix, South San Francisco, California, USA) (n=979) as previously described [60].

Quality control was implemented using PLINK [13]. Markers were included if they had a minor allele frequency over 0.01, and at least 95% complete genotyping increasing to 99% if the minor allele frequency was below 0.05 following the criteria set by the Wellcome Trust Case Control Consortium when they used data from the same platform [61]. To avoid batch artefacts, markers that differed significantly ($p<1*10^{-3}$) by genotyping centre/platform were excluded.

Individuals were excluded for ambiguous sex (n=115), abnormal heterozygosity (n=3), cryptic relatedness up to third-degree relatives by identity by descent (n=13), genotyping completeness less than 97% (n=5), non-European ethnicity admixture detected as outliers in an iterative EIGENSTRAT analyses of an LD-pruned dataset (n= 681), and invalid phenotypic information (n=24), resulting in 1,107 genotyped individuals for the analysis.

4.1.1.2.1 EIGENSTRAT

Sample selection from STAR*D for this analysis was limited to individuals of white European parentage. This was done in order to best replicate the individuals included

in the NEWMEDS sample who were all of white European ancestry. In order to ensure this was correct and to check self-reported ethnicity with genetic ethnicity as well as to correct to genetic architecture differences within European populations [15], we performed a principal component analysis using EIGENSTRAT [16]. An LD pruned dataset was used to remove confounding by local LD. The LD pruned dataset contained 83428 SNPs in low LD and excluded known region of long-range LD [16]. As the STAR*D is from an admixed population which includes mixed race individuals, this step was undertaken very stringently. A single EIGENSTRAT analysis was conducted with STAR*D and run iteratively. The first iteration was run with four HapMap populations (CEU, YRI, CHB, and JPT) to remove any individuals of non-caucasoid ancestry. A second iteration was run with three HapMap populations (CEU, MEX, and GIH) to ensure the sample was of white European ancestry only. Any individual was excluded as outliers and removed from further analyses as analysis if it was indicated they had strong African, Indian or Hispanic admixtures (n=548). After their exclusion, a third iteration was performed on just sample. Only individuals within 3 standard deviations of the mean from the first 10 principal components were included in the analysis (n=133). This step was essential to ensure outliers were removed and create as homogenous a sample as possible. A fourth analysis was run which detected no outliers and the first six principal components were nominally significant (Tracy-Widom $p<0.05$), and these were used as covariates in the genetic association analyses.

4.2 Imputation

To get the best coverage of the genome and overlap between the two samples, both NEWMEDS and STAR*D were imputed to include over 1.4 million markers using BEAGLE3.3 [62] and the HapMap phase 3 CEU population was the reference dataset.

Analyses were conducted on dosage data with estimated probability of each genotype, in order to consider the uncertainty of the imputation. The accuracy of imputation is reported for each result. Imputed data was analysed for association to outcome using the *dosage* command in PLINK[13] in any antidepressant taken (NEWMEDS n=1790, STAR*D n=1107) and in SRI treated individuals only (NEWMEDS n=1222, STAR*D n=1107). Imputation analyses also included covariates from EIGENSTRAT to correct for population stratification, as was done in the original analyses respectively for each sample.

4.3 Analysis

Meta-analysis was done using the '*meta'* command in PLINK [13]. Both fixed effects and random effects are reported. Two test of heterogeneity, Cochrane's Q statistic and I^2 heterogeneity index, are reported to demonstrate the heterogeneity between the two studies. Standard errors for the meta-analyses were calculated as the inverse sum of the individual studies variance divided by the square root of the number of informative individual studies.

4.4 Results

4.4.1 Any Antidepressant

2897 individuals were included in the meta-analysis which tested over 1.1 million SNPs between all NEWMEDS individuals treated with any antidepressants (n=1790) and STAR*D (n=1107). There were no genome wide significant results from the fixed or random effects meta-analysis between all of NEWMEDS and STAR*D. Table 4 shows the results from the fixed effects meta-analysis which reached genome wide suggestive threshold (p<5*10-6) and table 5 shows the results for the random effects meta-analysis reaching the genome wide suggestive threshold.

Table 4: Genome wide suggestive ($p<5*10^{-6}$) results from the fixed effects meta-analysis between any antidepressant NEWMEDS and STAR*D. CHR=chromosome. Q=Cochrane's Q statistic for heterogeneity. I=I^2 heterogeneity index. Regression coefficient is standardized and can be interpreted as a measure of effect size: it is the number of standard deviations in outcome per minor allele. Positive values of regression coefficient mean that carriers of more minor alleles had better treatment outcome. Negative values of regression coefficient mean that carriers of more minor alleles had worse outcomes.

CHR	SNP	Position	Gene	Allele	P-value	Regression Coefficient	Q	I
1	rs4650199	73424710	Intergenic	C	5.98E-07	0.131	0.136	55.01
1	rs11210177	73428409	Intergenic	A	7.59E-07	-0.133	0.168	47.46
1	rs12069039	73430673	Intergenic	C	1.19E-06	0.127	0.126	57.38
1	rs7514832	73431095	Intergenic	A	1.11E-06	-0.127	0.127	56.96
1	rs10890018	73434757	Intergenic	A	1.14E-06	-0.127	0.130	56.87
1	rs7522520	73437151	Intergenic	C	1.24E-06	-0.126	0.123	57.98
1	rs4650201	73438432	Intergenic	A	1.03E-06	-0.127	0.123	57.89
1	rs6684841	73479037	Intergenic	A	2.51E-07	0.134	0.079	67.6
1	rs11210187	73487821	Intergenic	C	5.97E-07	0.130	0.085	66.31
1	rs4650206	73493165	Intergenic	C	6.20E-07	0.130	0.090	65.28
1	rs4571923	73509150	Intergenic	A	1.13E-06	0.128	0.081	67.07
1	rs11210193	73516461	Intergenic	A	8.97E-07	0.128	0.072	69.17
1	rs7549372	73528117	Intergenic	A	4.59E-07	-0.131	0.050	74.06
1	rs7523829	73530705	Intergenic	A	1.12E-06	0.127	0.089	65.54
1	rs10789368	73586747	Intergenic	A	2.07E-06	0.123	0.048	74.32
1	rs11210220	73622243	Intergenic	G	3.37E-06	-0.123	0.079	67.62
1	rs11210222	73622275	Intergenic	C	3.73E-06	-0.123	0.070	69.48
1	rs6671130	73624275	Intergenic	G	2.84E-06	-0.124	0.082	66.95
1	rs6671002	73624340	Intergenic	A	3.22E-06	0.123	0.080	67.32
1	rs1923236	73626414	Intergenic	C	3.08E-06	-0.123	0.080	67.28
1	rs12044079	73626735	Intergenic	C	3.51E-06	0.122	0.084	66.42
1	rs1338654	73630312	Intergenic	G	3.23E-06	0.123	0.086	66.13
1	rs1885251	73638608	Intergenic	G	1.08E-07	0.138	0.020	81.5
1	rs12035848	73643489	Intergenic	A	2.96E-06	-0.123	0.082	66.86
1	rs7543202	73645473	Intergenic	A	3.52E-06	0.123	0.104	62.1
1	rs11210235	73650782	Intergenic	C	1.54E-07	-0.136	0.015	82.99
1	rs10465868	73653242	Intergenic	A	8.35E-08	0.139	0.027	79.68
1	rs7521446	73662978	Intergenic	C	6.52E-08	-0.140	0.022	81.04
1	rs11210242	73670397	Intergenic	C	1.12E-07	0.138	0.018	82.21
1	rs4113050	73682643	Intergenic	C	1.73E-07	-0.135	0.015	83.09
1	rs11210251	73685387	Intergenic	C	1.19E-07	0.137	0.017	82.44
1	rs11210255	73704325	Intergenic	A	3.65E-06	-0.120	0.032	78.23
1	rs12754690	73712486	Intergenic	C	5.25E-07	0.129	0.018	82.12
1	rs11210266	73736900	Intergenic	G	1.43E-06	0.124	0.016	82.67
11	rs1426651	96681529	Intergenic	C	3.13E-06	0.131	0.771	0
15	rs6598518	96891353	Intergenic	A	3.79E-06	-0.187	0.914	0
19	rs10426624	40365083	FCGBP	A	4.91E-06	-0.125	0.474	0
19	rs10426076	40365173	FCGBP	C	4.76E-06	-0.126	0.483	0
20	rs6040194	10777979	Intergenic	G	1.95E-06	-0.166	0.899	0

Table 5: Genome wide suggestive ($p<5*10^{-6}$) results from the random effects meta-analysis between any antidepressant NEWMEDS and STAR*D. CHR=chromosome. Q=Cochrane's Q statistic for heterogeneity. I=I^2 heterogeneity index. Regression coefficient is standardized and can be interpreted as a measure of effect size: it is the number of standard deviations in outcome per minor allele. Positive values of regression coefficient mean that carriers of more minor alleles had better treatment outcome. Negative values of regression coefficient mean that carriers of more minor alleles had worse outcomes.

CHR	SNP	Position	Gene	Allele	P-value	Regression Coefficient	Q	I
11	rs1426651	96681529	Intergenic	C	3.13E-06	0.131	0.771	0
15	rs6598518	96891353	Intergenic	A	3.79E-06	-0.186	0.914	0
19	rs10426624	40365083	FCGBP	A	4.91E-06	-0.125	0.474	0
19	rs10426076	40365173	FCGBP	C	4.76E-06	-0.126	0.483	0
20	rs6040194	10777979	Intergenic	G	1.95E-06	-0.166	0.899	0

4.4.2 Serotonergic Antidepressants

There were no genome wide significant results from the fixed or random effects meta-analysis between SRI treated NEWMEDS and STAR*D in the 1.1 million markers tested between the two studies in the 2329 individuals included (NEWMEDS n=1222 and STAR*D n=1107).

Table 6 shows the results from the fixed effects meta-analysis which reached genome wide suggestive threshold (p<5*10-6) and table 7 shows the results for the random effects meta-analysis reaching the genome wide suggestive threshold.

Table 6: Genome wide suggestive ($p<5*10^{-6}$) results from the fixed effects meta-analysis between serotonergic antidepressants in NEWMEDS and STAR*D. CHR=chromosome; Q=Cochrane's Q statistic for heterogeneity. I=I^2 heterogeneity index. Regression coefficient is standardized and can be interpreted as a measure of effect size: it is the number of standard deviations in outcome per minor allele. Positive values of regression coefficient mean that carriers of more minor alleles had better treatment outcome. Negative values of regression coefficient mean that carriers of more minor alleles had worse outcomes.

CHR	SNP	Position	Gene	Allele	P-value	Regression Coefficient	Q	I
1	rs6684841	73479037	Intergenic	A	1.54E-06	0.1405	0.089	65.68
1	rs11210193	73516461	Intergenic	A	1.84E-06	0.1393	0.111	60.73
1	rs7549372	73528117	Intergenic	A	8.33E-07	-0.1435	0.083	66.76
1	rs10789368	73586747	Intergenic	A	2.82E-06	0.1356	0.084	66.45
1	rs1885251	73638608	Intergenic	G	2.33E-07	0.1502	0.034	77.81
1	rs11210235	73650782	Intergenic	C	2.57E-07	-0.1493	0.028	79.17
1	rs10465868	73653242	Intergenic	A	2.43E-07	0.1498	0.040	76.28
1	rs7521446	73662978	Intergenic	C	1.88E-07	-0.1512	0.033	78.04
1	rs11210242	73670397	Intergenic	C	3.93E-07	0.1471	0.024	80.44
1	rs4113050	73682643	Intergenic	C	6.11E-07	-0.1445	0.020	81.61
1	rs11210251	73685387	Intergenic	C	4.10E-07	0.1469	0.023	80.61
1	rs505725	73702993	Intergenic	A	3.28E-06	-0.1341	0.083	66.63
1	rs622421	73703081	Intergenic	A	4.98E-06	0.1321	0.058	72.22
1	rs11210255	73704325	Intergenic	A	2.20E-06	-0.1366	0.076	68.15
1	rs12754690	73712486	Intergenic	C	1.88E-06	0.1366	0.021	81.11
1	rs11210266	73736900	Intergenic	G	4.04E-06	0.1326	0.020	81.39
9	rs7870795	99613941	ZNF782	C	3.15E-06	0.1485	0.713	0
9	rs7859751	99615709	ZNF782	A	2.84E-06	0.1492	0.703	0
11	rs1426651	96681529	ADAMTSL3	C	2.29E-06	0.1469	0.382	0
15	rs1566088	84607033	ADAMTSL3	C	4.83E-06	0.1342	0.180	44.41
15	rs4887218	84608081	ADAMTSL3	A	4.40E-06	-0.1346	0.184	43.42
15	rs7181181	84610029	ADAMTSL3	C	4.17E-06	-0.1351	0.174	45.93
15	rs8041327	84610811	ADAMTSL3	C	4.34E-06	0.1349	0.181	44.15

Table 7: Genome wide suggestive ($p<5*10^{-6}$) results from the random effects meta-analysis between serotonergic antidepressants in NEWMEDS and STAR*D. CHR=chromosome. Q=Cochrane's Q statistic for heterogeneity. I=I^2 heterogeneity index. Regression coefficient is standardized and can be interpreted as a measure of effect size: it is the number of standard deviations in outcome per minor allele. Positive values of regression coefficient mean that carriers of more minor alleles had better treatment outcome. Negative values of regression coefficient mean that carriers of more minor alleles had worse outcomes.

CHR	SNP	Position	Gene	Allele	P-value	Regression Coefficient	Q	I
9	rs7870795	99613941	ZNF782	C	3.15E-06	0.1485	0.713	0
9	rs7859751	99615709	ZNF782	A	2.84E-06	0.1492	0.703	0
11	rs1426651	96681529	Intergenic	C	2.29E-06	0.1469	0.382	0

5 Polygene scoring

Recent work in other psychiatric traits, such as schizophrenia [63,64], has suggested a large number of common variants may be playing a role in the development of the trait. While both our main analysis in NEWMEDS and the meta-analysis with STAR*D had sufficient power to detect clinically significant association, there could be multiple weak across the genome which may offer insight into the other mechanism of antidepressant response.

5.1 Method

The methodology for polygene scoring has been described extensively elsewhere [63]. Here, scores were created based on analysis from the imputed data from NEWMEDS. The risk alleles were weighted by the strength of their association. SNPs were removed if they had a low minor allele frequency (MAF<0.02) or resided in the major histocompatibility complex (MHC) region. The dataset was pruned for linkage disequilibrium ($r^2 < 0.25$) removing SNPs that share more than 80% variance. Two polygene tests were carried out. The first scored the result from any antidepressant analysis in NEWMEDS to predict in STAR*D. The second scored the results from the serotonergic antidepressant analysis in NEWMEDS to predict in STAR*D. The resulting datasets for both included 142,492 SNPs. Thirteen scores were calculated based on progressive p-value thresholds (p < 0.001, 0.01, 0.05, 0.1, 0.2, 0.3, 0.4, 0.5, 0.6, 0.7, 0.8 0.9, and 1.0). The resulting scores were tested as predictors of improvement (percentage adjusted change, see Definition of antidepressant response phenotype Supplementary Materials section 1.4) in STAR*D using linear regression.

5.2 Results

5.2.1 Any Antidepressant

The results from the NEWMEDS analysis including all individuals given any antidepressants (n=1790) was scored and used to predict improvement in STAR*D (n=1107) using a linear regression. Across the thirteen different scoring bin used there was no significant prediction (Table 8).

Table 8: Results from the polygene scoring analysis. Progressive p-value ranges were use to score risk alleles from NEWMEDS any antidepressants to predict in STAR*D. Scoring bin and the Range informs of the p-values included from NEWMEDS. r2 and p-value are the results from the prediction analysis in STAR*D. r2 = proportion of variance explained.

Scoring Bin	Range		STAR*D Prediction Results	
	p-value Min	p-value Max	r2	p-value
S0	0	0.0001	0.0017	0.171
S1	0	0.001	4.80E-06	0.941
S2	0	0.01	0.0003	0.576
S3	0	0.05	0.0005	0.444
S4	0	0.1	9.02E-05	0.750
S5	0	0.2	0.0009	0.322
S6	0	0.3	0.0017	0.172
S7	0	0.4	0.0024	0.101
S8	0	0.5	0.0024	0.099
S9	0	0.6	0.0024	0.099
S10	0	0.7	0.0024	0.099
S11	0	0.8	0.0024	0.099
S12	0	0.9	0.0024	0.099

5.2.2 Serotonergic Antidepressant

Results from the NEWMEDS analysis including individual taking serotonergic antidepressants (n=1222) was scored and used to predict improvement in STAR*D (n=1107) using a linear regression. Across the thirteen different scoring bin used there was no significant prediction (Table 9).

Table 9: Results from the polygene scoring analysis. Progressive p-value ranges were use to score risk alleles from NEWMEDS serotonergic antidepressants to predict in STAR*D. Scoring bin and the Range informs of the p-values included from NEWMEDS. r2 and p-value are the results from the prediction analysis in STAR*D. r2 = proportion of variance explained.

Scoring Bin	Range		STAR*D Prediction Results	
	p-value min	p-value max	r2	p-value
S0	0	0.0001	1.28E-05	0.904
S1	0	0.001	3.47E-05	0.843
S2	0	0.01	0.0014	0.203
S3	0	0.05	0.0008	0.344
S4	0	0.1	2.63E-06	0.957
S5	0	0.2	0.0005	0.437
S6	0	0.3	0.0007	0.383
S7	0	0.4	0.0005	0.442
S8	0	0.5	0.0006	0.397
S9	0	0.6	0.0012	0.251
S10	0	0.7	0.0013	0.220
S11	0	0.8	0.0014	0.209
S12	0	0.9	0.0014	0.217

6 Study level meta-analysis between the samples in NEWMEDS

In the main paper, an individual data analysis was undertaken bringing together data from five studies. We further conducted the analyses in each individuals study (GenDep, GenPod, GODS, Pfizer, Glaxo-Smith Kline) and meta-analysed the results. The results of the meta-analysis are presented here, and compared to the results from the individual data analysis.

6.1 Individual study results (QQ plots)

Samples varied in size with post genotype quality control samples being: GenDep n=798, GenPod n=477, Pfizer n=311, Glaxo-Smith Kline n=132, and GODS n=73. Linear regressions were undertaken in each study separately to test for association in 520,978 SNPs on adjusted percentage change in depression severity under an additive genetic model implemented in PLINK [13]. To replicate the analytical plan, three meta-analyses were done. The first analysis was a linear regression undertaken in

each of the individual studies, and included all individuals. The second linear regression was undertaken only on individuals given SRI antidepressants, which included all of Pfizer (n=311), Glaxo-Smith Kline (n=132) and GODS (n=73), and part of GenDep (n=464) and GenPod (n=242). The third linear regression was undertaken only in individuals taking noradrenergic antidepressant, which only included part of GenDep (n=333) and GenPod (n=235). Linear regressions were run under the same conditions as in the individual data analysis. In brief, the outcome was adjusted percentage change in depression severity with four covariates to control for population stratification as highlighted in the EIGENSTRAT analysis.

Analyses undertaken in individual studies showed a uniform distribution of p-values with no inflation in the test statistic (Figure 1, Figure 2 and Table 10). Only GenDep and GenPod samples are shown for the serotonergic specific analysis, as the entire sample and serotonergic specific samples for Pfizer, Glaxo-Smith Kline and GODS are the same.

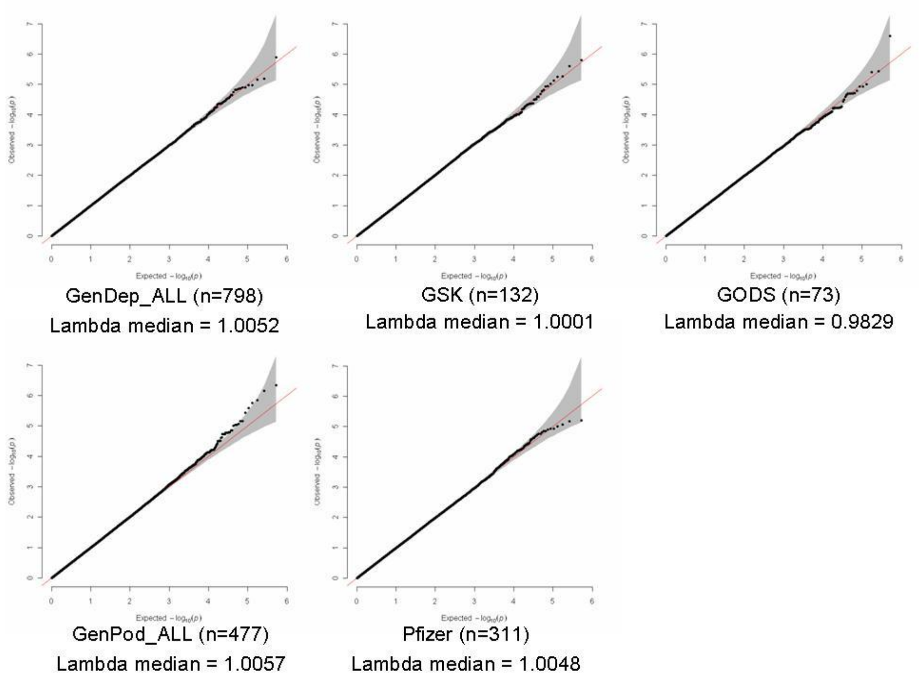

Figure 1: Quantile-quantile plots from the genome-wide linear regression analyses undertaken on the five samples separately. All individuals were included from the five separate samples for this analysis. Each sample shows a uniform distribution of p-values with no major deviation in the median lambda statistic.

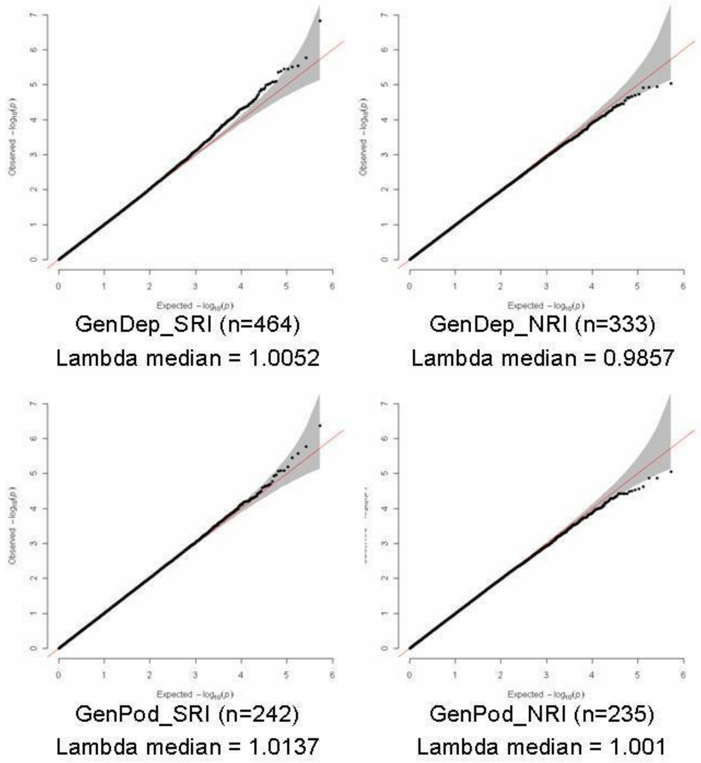

Figure 2: Quantile-quantile plots from the genome-wide linear regression analyses undertaken in the samples with two drugs differing on mechanism of action (serotonergic versus noradrenergic). Each sample shows a uniform distribution of p-values with no major deviation in the median lambda statistic.

Table 10: Median lambda values for the analyses undertaken in the five separate studies.

Study	Analysis	n	Median Lambda
GenDep	Entire Sample	798	1.0052
GenDep	SRI only	464	1.0052
GenDep	NRI only	333	0.9857
GenPod	Entire Sample	477	1.0057
GenPod	SRI only	242	1.0137
GenPod	NRI only	235	1.001
Pfizer	Entire Sample/ SRI only	311	1.0048
Glaxo-Smith Kline	Entire Sample/ SRI only	132	1.0001
GODS	Entire Sample/ SRI only	73	0.9829

6.2 Study level meta-analysis method

Study level meta-analyses were undertaken using the '*meta'* command in PLINK [13]. Results presented are for fixed effects, unless the marker showed high heterogeneity between studies judged from two test of heterogeneity, Cochrane's Q statistic and I^2 heterogeneity index. Standard errors for the meta-analyses were calculated as the inverse sum of the individual studies variance divided by the square root of the number of informative individual studies.

6.3 Results

6.3.1 Study level meta-analysis of response to any antidepressant (n=1790)

Results from the study level meta-analysis were compared to the results from the individual data level analysis reported in the main part of the paper. Correlation between the analysis undertaken in the main section of the manuscript and the study level meta-analysis using fixed effects outcomes was Pearson's correlation=0.981609 (-log10 p-values) and Spearman's = 0. 9650631 (-log10 p-values) (Figure 3).

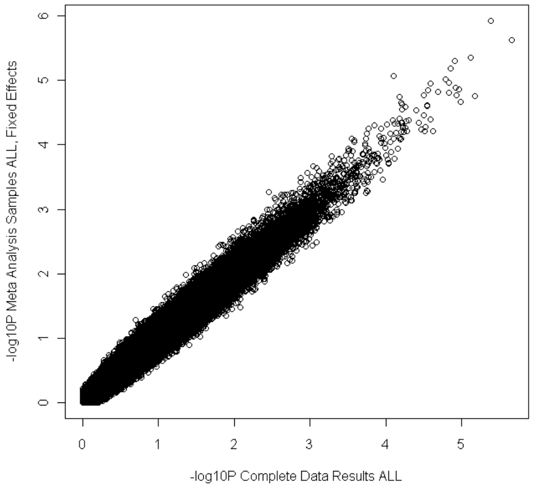

Figure 3: Plot of -log10 P-values. The x-axis is the results from the individual date analysis in the entire sample reported in the main paper. The y-axis is the study level meta-analysis results for fixed effects.

SNPs with p-values below the suggestive evidence threshold of $p<5*10^{-6}$ are shown in Table 11, along with those SNPs regression coefficient, standard error and p-values from individual studies and the individual data analysis from the main part of the paper. Cochran's Q statistic for all four SNPs at the suggestive level in the study level meta-analysis was not significant and had I^2 indices of below 0.2, indicating no major heterogeneity between the studies. Thus the results presented here are for the fixed effects from these SNPs. Of the four SNPs below the suggestive significance level for the study level meta-analysis, two had reached the suggestive significance level in the individual data analysis presented in the main part of the paper (rs10818702 study level meta-analysis fixed effects $p=2.38*10^{-6}$, individual data analysis $p=2.19*10^{-6}$ and rs11624702 study level meta-analysis fixed effects $p=1.18*10^{-6}$, individual data analysis $p=4.08*10^{-6}$). The other two SNPs were strongly associated in the individual data analysis but failed to reach the suggestive evidence cut-off.

Table 11: Study level meta-analysis (referred to as META below) results for SNPs with p-values below the suggestive significance level of $5*10^{-6}$ for fixed effects meta-analysis for the adjusted percentage change in depression severity in the entire sample of antidepressant treated individuals under an additive genetic model. Regression coefficient is standardized and can be interpreted as a measure of effect size: it is the number of standard deviations in outcome per minor allele. Positive values of regression coefficient mean that carriers of more minor alleles had better treatment outcome. Negative values of regression coefficient mean that carriers of more minor alleles had worse outcomes.

CHR	Position	SNP	A1	Study	N	p-value	Regression	SE
2	65084243	rs3770705	G	**META**	**5 Studies**	**4.37E-06**	**0.168**	
				GenDep	793	2.83E-05	0.224	0.053
				GenPod	473	8.54E-02	0.124	0.072
				GODS	73	8.51E-01	0.073	0.387
				Pfizer	309	4.29E-01	0.068	0.086
				GSK	131	9.54E-02	0.226	0.135
				Individual data analysis	*1779*	*7.49E-06*	*0.162*	*0.038*
9	38629318	rs12340088	C	**META**	**5 Studies**	**4.97E-06**	**0.199**	
				GenDep	794	1.11E-02	0.165	0.065
				GenPod	477	1.43E-03	0.261	0.081
				GODS	73	9.95E-01	0.002	0.414
				Pfizer	311	8.92E-02	0.172	0.101
				GSK	132	1.16E-01	0.274	0.173
				Individual data analysis	*1787*	*1.24E-05*	*0.185*	*0.042*
9	124285545	rs10818702	A	**META**	**5 studies**	**2.38E-06**	**-0.193**	
				GenDep	797	4.38E-03	-0.168	0.059
				GenPod	476	2.59E-03	-0.242	0.080
				GODS	73	3.91E-01	-0.355	0.411
				Pfizer	311	4.72E-03	-0.282	0.099
				GSK	132	7.78E-01	0.0423	0.150
				Individual data analysis	*1789*	*2.19E-06*	*-0.190*	*0.040*
14	46588187	rs11624702	A	**META**	**5 studies**	**1.18E-06**	**0.162**	
				GenDep	797	5.58E-04	0.166	0.048
				GenPod	477	1.48E-02	0.158	0.064
				GODS	73	9.98E-01	-0.001	0.391
				Pfizer	311	1.87E-01	0.108	0.082
				GSK	132	2.19E-02	0.274	0.118
				Individual data analysis	*1790*	*4.08E-06*	*0.151*	*0.033*

6.3.1.1 Forest plot of top results from the study level meta-analysis

Figures 4 through 7 are forest plots for the SNPs which reached the genome wide suggestive level of $p<5*10^{-6}$ in the study level meta-analysis undertaken for response to any antidepressant (n=1790).

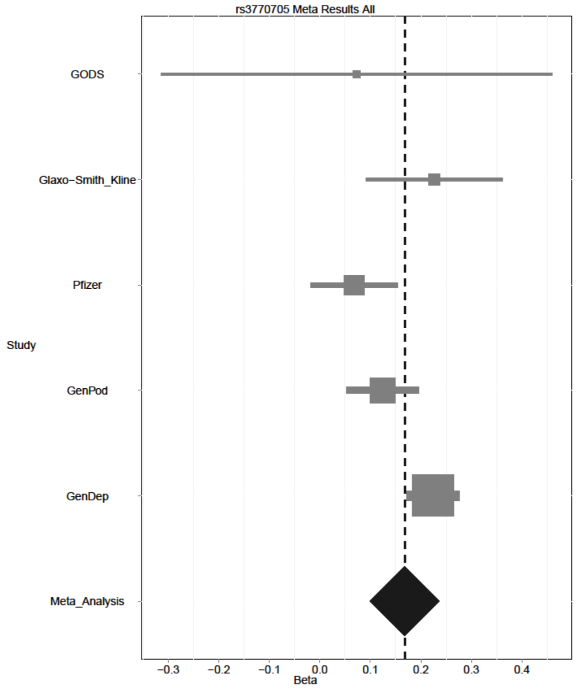

Figure 4: Forest plot for rs3770705. Results from the five individual studies are shown with lines representing 95% confidence intervals and boxes indicating individual study effect size. The black line is the beta value from the study level meta-analysis referred to as “Meta_Analysis” above.

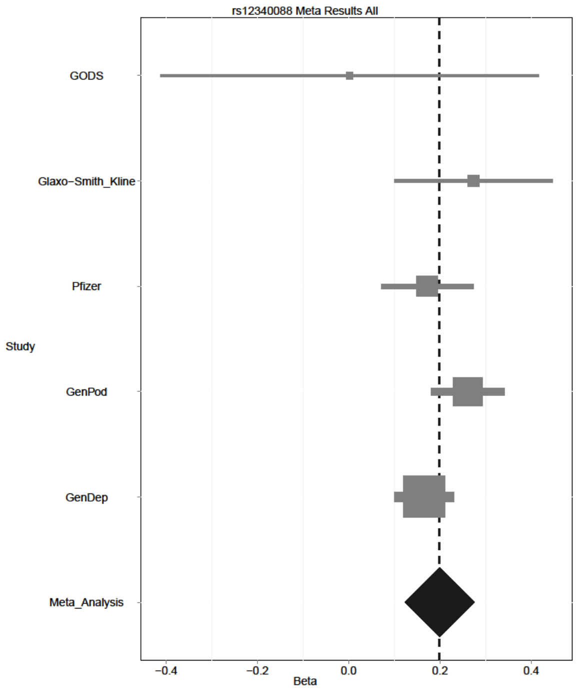

Figure 5: Forest plot for rs12340088. Results from the five individual studies are shown with lines representing 95% confidence intervals and boxes indicating individual study effect size. The black line is the beta value from the study level meta-analysis referred to as “Meta_Analysis” above.

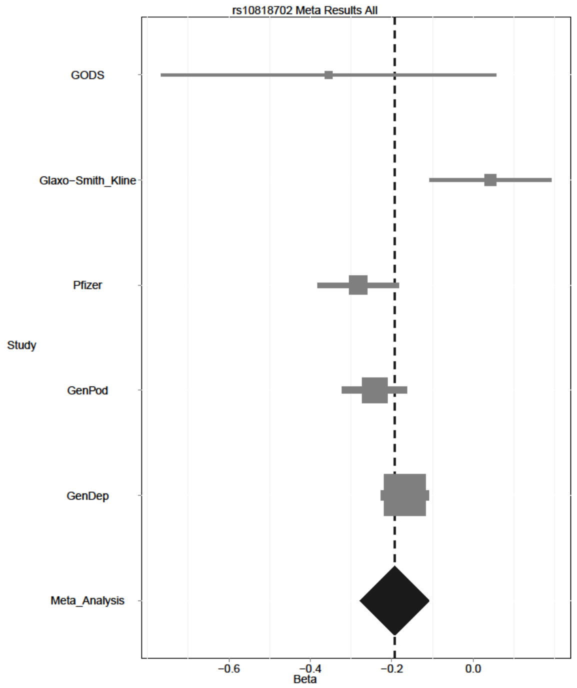

Figure 6: Forest plot for rs10818702. Results from the five individual studies are shown with lines representing 95% confidence intervals and boxes indicating individual study effect size. The black line is the beta value from the study level meta-analysis referred to as “Meta_Analysis” above.

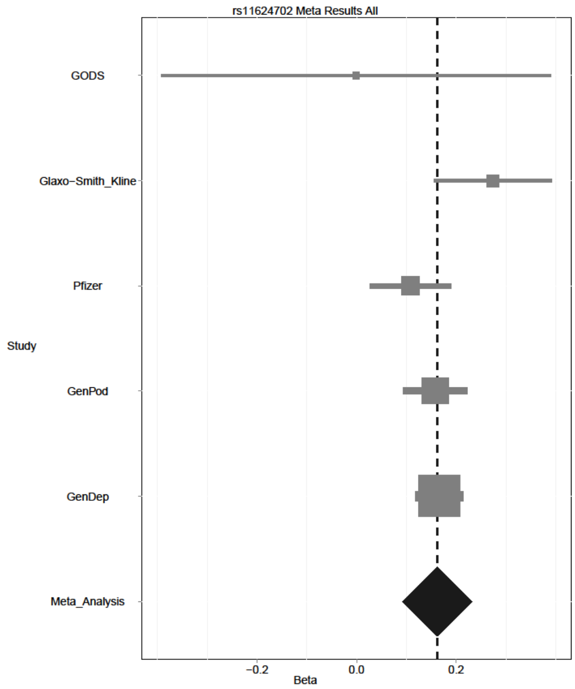

Figure 7: Forest plot for rs11624702. Results from the five individual studies are shown with lines representing 95% confidence intervals and boxes indicating individual study effect size. The black line is the beta value from the study level meta-analysis referred to as "Meta_Analysis" above.

6.3.2 Study level meta-analysis of response to SRIs (n=1222)

Study level meta-analysis from the five individual studies for individuals given serotonergic antidepressants was undertaken using PLINK [13]. This analysis included all individuals from the Pfizer, Glaxo-Smith Kline and GODS studies as well as some individuals from GenDep (n=464) and GenPod (n=242) studies. Results from the study level meta-analysis were compared to the results from the individual data analysis reported for individuals given serotonergic antidepressants in the main part of the paper. Correlation between the individual data analysis and the study level meta-analysis in the individuals taking serotonergic antidepressants using fixed effects outcomes was Pearson's correlation = 0.9692422 (-log10 p-values) and Spearman's = 0.9445827 (-log10 p-values) (Figure 8).

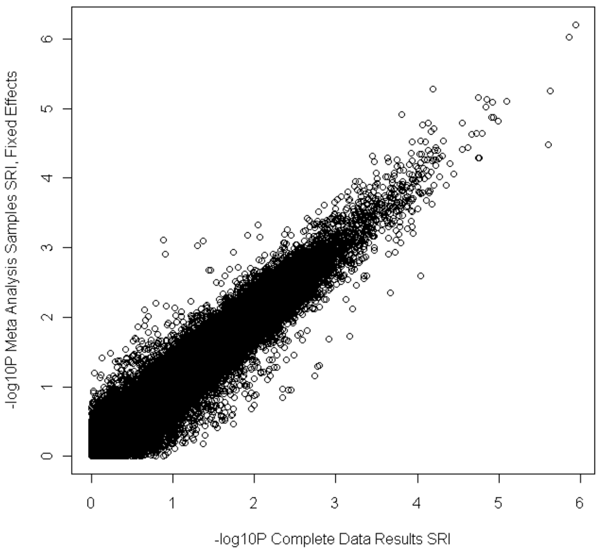

Figure 8: Plot of -log10 P-values. The x-axis is the results from the individual data analysis for individuals on serotonergic antidepressants reported in the main paper. The y-axis is the study level meta-analysis results for fixed effects for individuals from the five studies taking serotonergic antidepressants.

SNPs with p-values below the suggestive evidence threshold of $p<5*10^{-6}$ are shown in Table 12, along with those SNPs regression coefficient, standard error and p-values from individual studies and the individual data analysis from the main part of the paper. Cochran's Q statistic for both SNPs at the suggestive level in the study level meta-analysis was not significant and had I^2 indices of 0, indicating no heterogeneity between the studies. Thus the results presented here are for the fixed effects from these SNPs. Both SNPs were also associated in the individual data analysis presented in the main part of the paper below the suggestive evidence threshold ($p<5*10^{-6}$).

Table 12: Study level meta-analysis (referred to as META below) results for the adjusted percentage change in depression severity in SRI-treated individuals under an additive genetic model. SNPs reported had p-values below the suggestive significance level of $5*10^{-6}$ for fixed effects meta-analysis. Regression coefficient is standardized and can be interpreted as a measure of effect size: it is the number of standard deviations in outcome per minor allele. Positive values of regression coefficient mean that carriers of more minor alleles had better treatment outcome. Negative values of regression coefficient mean that carriers of more minor alleles had worse outcomes.

CHR	BP	SNP	A1	Study	N	p-value	Regression Coefficient	SE
5	164692074	rs10515893	A	**META**	**5 studies**	**9.30E-07**	**-0.221**	
				GenDep	464	3.13E-02	-0.155	0.072
				GenPod	242	5.53E-02	-0.199	0.103
				GODS	73	4.01E-01	-0.356	0.421
				Pfizer	311	4.02E-04	-0.303	0.085
				GSK	132	4.25E-02	-0.262	0.128
				Individual data analysis	*1222*	*1.37E-06*	*-0.223*	*0.048*
12	47433214	rs10783282	A	**META**	**5 studies**	**6.17E-07**	**-0.251**	
				GenDep	464	1.99E-04	-0.294	0.079
				GenPod	242	4.60E-01	-0.089	0.121
				GODS	73	2.98E-01	-0.501	0.478
				Pfizer	311	6.34E-04	-0.317	0.092
				GSK	132	4.04E-01	-0.132	0.158
				Individual data analysis	*1222*	*1.16E-06*	*-0.247*	*0.054*

6.3.2.1 Forest plot of top results from the study level meta-analysis

Figures 9 and 10 are forest plots for the SNPs which reached the genome wide suggestive level of $p<5*10^{-6}$in the study level meta-analysis undertaken for response to serotonergic antidepressant (n=1222).

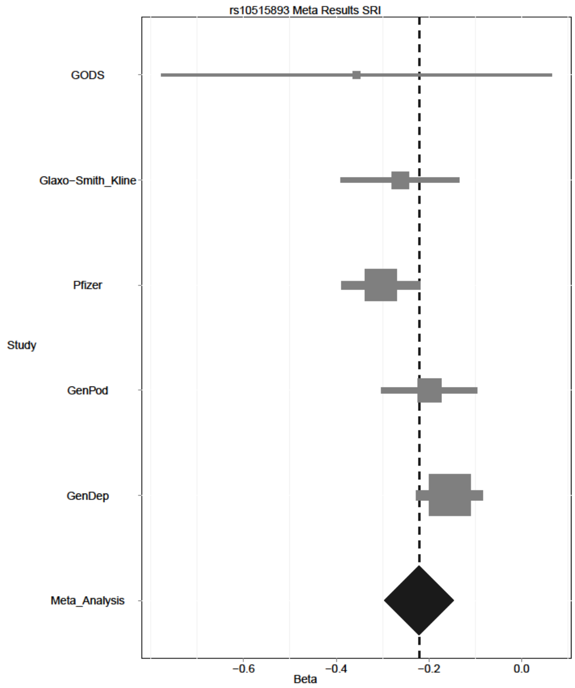

Figure 9: Forest plot for rs10515893. Results from the five individual studies are shown with lines representing 95% confidence intervals and boxes indicating individual study effect size. The black line is the beta value from the study level meta-analysis referred to as "Meta_Analysis" above.

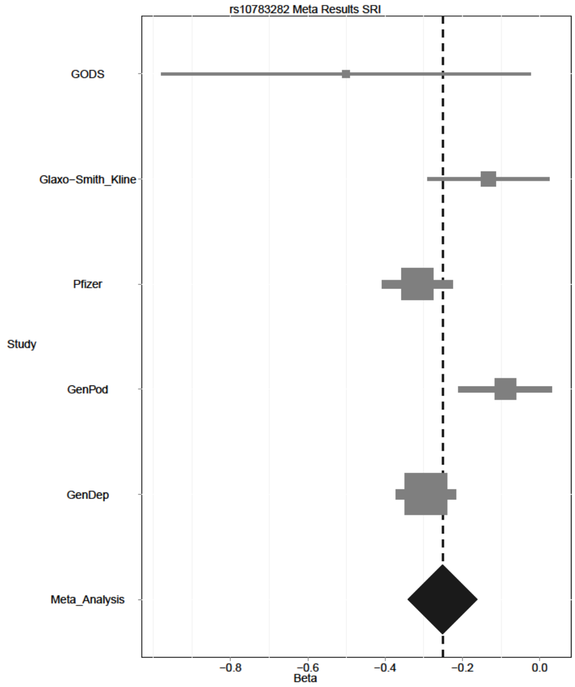

Figure 10: Forest plot for rs10783282. Results from the five individual studies are shown with lines representing 95% confidence intervals and boxes indicating individual study effect size. The black line is the beta value from the study level meta-analysis referred to as "Meta_Analysis" above.

6.3.3 Study level meta-analysis of response to NRIs (n=568)

Study level meta-analysis from the two studies with individuals given noradrenergic antidepressants was undertaken using PLINK [13]. This analysis included only some individuals from GenDep (n=333) and GenPod (n=235) studies. Results from the study level meta-analysis were compared to the results from the individual data analysis reported for individuals given noradrenergic antidepressants in the main part of the paper. Correlation between the individual data analysis and study level meta-analysis in the entire sample using fixed effects outcomes was Pearson's correlation = 0.9953033 (-log10 p-values) and Spearman's = 0.9909853 (-log10 p-values) (Figure 11).

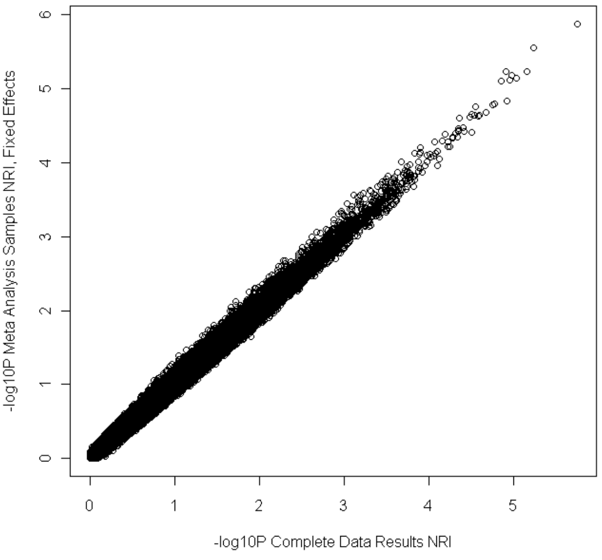

Figure 11: Plot of -log10 P-values. The x-axis is the results from the individual data analysis for individuals on noradrenergic antidepressants reported in the main paper. The y-axis is the study level meta-analysis results for fixed effects for individuals from the two studies given noradrenergic antidepressants.

SNPs with p-values below the suggestive evidence threshold of $p<5*10^{-6}$ are shown in Table 13, along with those SNPs regression coefficient, standard error and p-values from individual studies and the individual data analysis from the main part of the

paper. Cochran's Q statistic for all three SNPs at the suggestive level in the study level meta-analysis was not significant and had I^2 indices of 0, indicating no heterogeneity between the studies. Thus the results presented here are for the fixed effects from these SNPs. Two SNPs from the study level meta-analysis were the two SNPs associated in the individual data analysis below the suggestive evidence threshold ($p<5*10^{-6}$) presented in the main part of the paper. The third SNP at the suggestive level in the study level meta-analysis was associated in the individual data analysis just under the significance threshold ($5.83*10^{-6}$, rs17810534).

Table 13: Study level meta-analysis (referred to as META below) results for the adjusted percentage change in depression severity in SRI-treated individuals under an additive genetic model. SNPs reported had p-values below the suggestive significance level of $5*10^{-6}$ for fixed effects meta-analysis. Regression coefficient is standardized and can be interpreted as a measure of effect size: it is the number of standard deviations in outcome per minor allele. Positive values of regression coefficient mean that carriers of more minor alleles had better treatment outcome. Negative values of regression coefficient mean that carriers of more minor alleles had worse outcomes.

CHR	BP	SNP	A1	Study	N	p-value	Regression Coefficient	SE
6	129928992	rs17810534	G	**META**	**2 studies**	**2.80E-06**	**-0.368**	
				GenDep	332	1.19E-05	-0.412	0.093
				GenPod	234	8.61E-02	-0.255	0.148
				Individual data analysis	*566*	*5.83E-06*	*-0.360*	*0.079*
7	27676198	rs13237776	A	**META**	**2 studies**	**1.32E-06**	**-0.338**	
				GenDep	333	1.48E-04	-0.324	0.084
				GenPod	235	3.42E-03	-0.370	0.125
				Individual data analysis	*568*	*1.77E-06*	*-0.336*	*0.070*
7	27677080	rs12534474	A	**META**	**2 studies**	**1.32E-06**	**-0.338**	
				GenDep	333	1.48E-04	-0.324	0.084
				GenPod	235	3.42E-03	-0.370	0.125
				Individual data analysis	*568*	*1.77E-06*	*-0.336*	*0.070*

6.3.3.1 Forest plot of top results from the study level meta-analysis

Figures 12 through 15 are forest plots for the SNPs which reached the genome wide suggestive level of $p<5*10^{-6}$ in the study level meta-analysis undertaken for response to noradrenergic antidepressant (n=568).

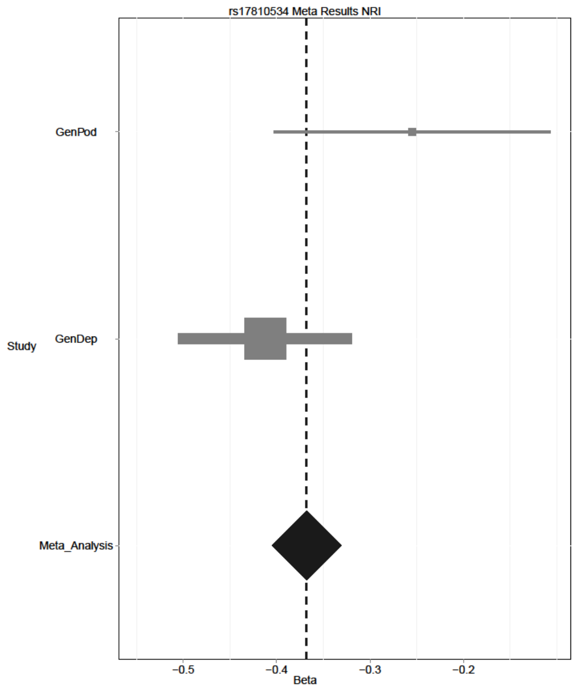

Figure 12: Forest plot for rs17810534. Results from the five individual studies are shown with lines representing 95% confidence intervals and boxes indicating individual study effect size. The black line is the beta value from the study level meta-analysis referred to as “Meta_Analysis” above.

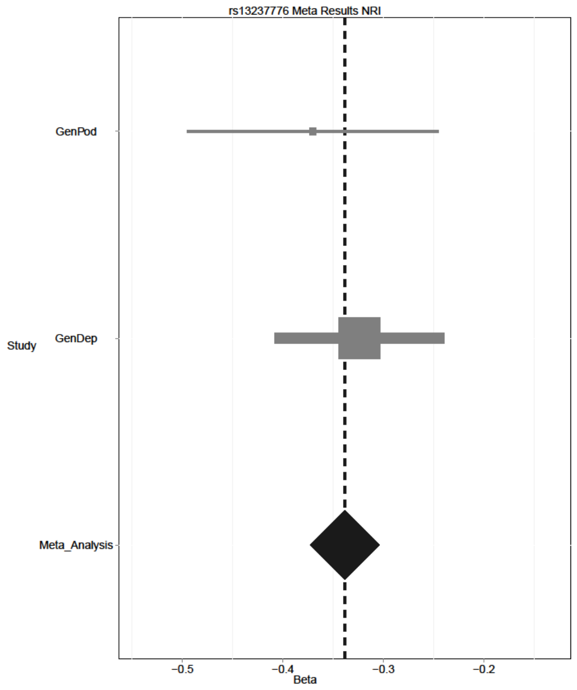

Figure 13: Forest plot for rs13237776. Results from the five individual studies are shown with lines representing 95% confidence intervals and boxes indicating individual study effect size. The black line is the beta value from the study level meta-analysis referred to as "Meta_Analysis" above.

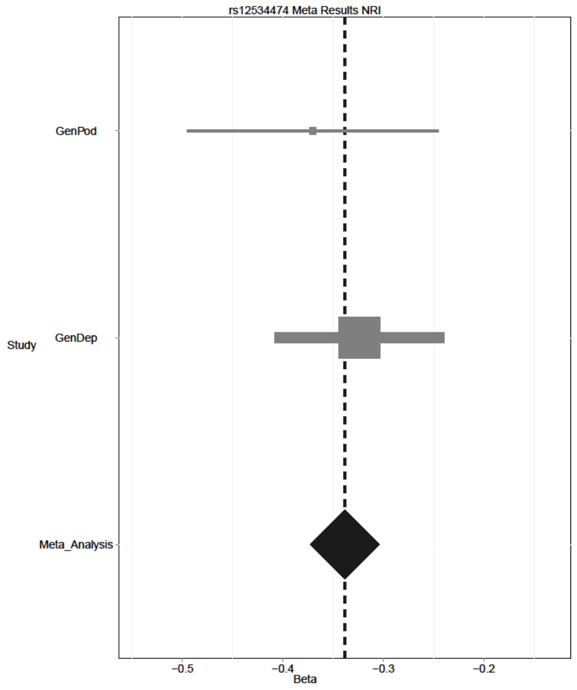

Figure 14: Forest plot for rs12534474. Results from the five individual studies are shown with lines representing 95% confidence intervals and boxes indicating individual study effect size. The black line is the beta value from the study level meta-analysis referred to as "Meta_Analysis" above.

7 Candidate gene analysis in NEWMEDS

7.1 Exploration of candidate genes

We extracted 3,802 markers located within the coding region and 20kb window 5' and 3' of the coding region of 118 candidate genes, selected based on recent literature reviews on antidepressant pharmacogenetics [22,60,65-67], and implicated in antidepressant action, or aetiology of mental illness. Top associations from the four analyses are reported.

7.2 Results

None of the 3,802 markers in 118 candidate genes were significantly associated with outcome after correction for multiple testing (Bonferroni-corrected p value=$1.3*10^{-5}$). The strongest associations with response to any antidepressant in the whole sample were within *GRIK3* (rs11801494 p=$1.97*10^{-4}$), *GRIK4* (rs4445646 p=$8.45*10^{-4}$) and. *PDE9A* (rs2245730 p=$8.77*10^{-4}$). In the drug-specific analyses, *GRIK1* (rs363512 p=$3.3*10^{-4}$) and *UST* (rs2500535 p=$6.41*10^{-4}$) were the most interesting results for NRI response, and *HTR4* (rs1833704 p=$4.97*10^{-4}$), *GRIK4* (rs4445646 p=$7.46*10^{-4}$) and ZNF804A (rs2369595 p=$9.32*10^{-4}$) were associated with SRI response. For differential response to SRI and NRI antidepressants, the top results were in *NTRK2* (rs7875184 p=0.0008), *CLOCK* (rs1522113 p=0.0011), *PRKCH* (rs1033908 p=0.0015) and *NEGR1* (rs6683448 p=0.0018).

A full list of top SNP for each gene tested for association in the candidate gene analysis can be found in Table 14 for response to any antidepressant (n=1790), Table 15 for response to serotonergic antidepressants (n=1222), Table 16 for response to noradrenergic antidepressants (n=568), and Table 17 for gene by drug interaction results.

Table 14: Candidate gene results from analysis of SNP markers on the adjusted percentage change in depression severity in the whole sample of 1790 antidepressant-treated individuals. Only the top associated SNP is shown each gene. Regression coefficient is standardized and can be interpreted as a measure of effect size: it is the number of standard deviations in outcome per minor allele. Positive values of regression coefficient mean that carriers of more minor alleles had better treatment outcome. Negative values of regression coefficient mean that carriers of more minor alleles had worse outcomes.

		Entire Sample (n=1790)			
Gene symbol	Number of SNPs tested	Top SNP	Regression Coefficient	SE	p-Value
ABCB1	59	rs6946119	0.0490	0.0381	0.1948
ACE	11	rs4459609	0.0920	0.0336	0.0064
ADRA1A	67	rs2291776	-0.1880	0.0652	0.0039
ADRA1B	21	rs10053468	-0.1310	0.0656	0.0454
ADRA2A	8	rs7908645	0.0370	-0.0309	0.3986
ADRA2B	5	rs749457	-0.0090	0.0340	0.7968
ADRA2C	5	rs12506413	-0.0970	0.0424	0.0234
ADRB1	16	rs4917675	0.1090	0.0386	0.0049
AKT1	4	rs4983387	0.0810	0.0499	0.1043
ANK3	118	rs12355908	-0.0790	0.0374	0.0353
ANKK1	15	rs877138	-0.0840	0.0351	0.0168
AVPR1A	10	rs7308008	0.0880	0.0480	0.0678
AVPR1B	5	rs28425623	0.0330	0.0511	0.5146
AVPR2	5	rs4898457	-0.0420	0.0418	0.3185
BDNF	14	rs7934165	0.0330	0.0772	0.0176
CACNA1C	191	rs2239037	-0.1010	0.0328	0.0020
CCL28	9	rs779850	0.0760	0.0426	0.0775
CD3E	6	rs2277289	-0.1040	0.0354	0.0034
CLOCK	10	rs11932595	0.0640	0.0335	0.0576
CNR1	22	rs806368	0.1130	0.0404	0.0053
COMT	28	rs165599	0.0970	0.0359	0.0069
CREB1	7	rs2194430	-0.0790	0.1002	0.4308
CRHR1	22	rs12185268	0.0730	0.0393	0.0633
CRHR2	19	rs24003	-0.0550	0.0336	0.1043
CXCR4	8	rs12691874	0.0540	0.0331	0.1054
DRD1	23	rs11954565	-0.0550	0.0338	0.1040
DRD2	29	rs2734849	-0.0750	0.0336	0.0251
DRD3	23	rs6787134	0.0690	0.0471	0.1422
DRD4	6	rs7932167	-0.0470	0.0390	0.2307

DRD5	3	rs1533615	0.1020	0.0790	0.1969
DTNBP1	31	rs9396593	-0.0840	0.0385	0.0288
EDNRB	5	rs9544635	-0.0710	0.0562	0.2034
FGFR1	13	rs13317	0.0610	0.0385	0.1153
FGFR2	33	rs4752566	0.0610	0.0329	0.0662
FGFR3	1	rs743682	-0.0760	0.0574	0.1881
FGFR4	4	rs451643	0.0500	0.0355	0.1594
FKBP5	22	rs10456432	-0.0580	0.0414	0.1602
FLT1	42	rs10507385	-0.0640	0.0366	0.0830
FTO	97	rs17219084	0.0790	0.0337	0.0203
GNAS	16	rs234623	0.0910	0.0333	0.0062
GNB3	16	rs5439	-0.1260	0.0561	0.0249
GNPDA2	2	rs12640665	0.1300	0.0675	0.0546
GRIA1	94	rs11746246	0.1690	0.0496	0.1692
GRIA2	9	rs10025251	0.0630	-0.0776	0.0630
GRIA3	60	rs5911623	0.0200	-0.0880	0.0202
GRIA4	57	rs17391295	0.0050	0.1705	0.0050
GRIK1	116	rs462606	0.0070	-0.1155	0.0068
GRIK2	159	rs6926170	0.0110	0.0837	0.0112
GRIK3	31	rs11801494	0.0001	0.2095	0.0002
GRIK4	107	rs4445646	0.0008	0.1157	0.0008
GRIK5	5	rs2217342	0.1140	-0.0925	0.1139
GRIN1	3	rs4880094	0.2060	-0.0497	0.2061
GRIN2A	147	rs9928984	0.0760	-0.1474	0.0758
GRIN2B	173	rs10459061	0.0070	0.1980	0.0069
GRIN2C	5	rs3803783	0.3130	0.0371	0.3126
GRIN2D	9	rs275844	0.3100	0.0506	0.3102
GRIN3A	61	rs1415644	0.0190	-0.0932	0.0188
GSK3A	1	rs11878620	0.3720	0.0572	0.3724
GSK3B	24	rs11919783	0.0566	-0.1035	0.0566
HTR1A	2	rs1364043	0.2600	-0.0441	0.2603
HTR1B	13	rs9352483	0.0540	0.0901	0.0541
HTR2A	50	rs4942578	0.0020	0.1376	0.0016
HTR2B	4	rs13394402	0.0100	0.1116	0.0101
HTR2C	15	rs498207	0.1920	0.0500	0.1918

HTR3A	22	rs10891611	0.1070	0.0732	0.1071
HTR3B	13	rs3891484	0.1520	-0.0672	0.1519
HTR4	41	rs2068190	0.0320	0.0714	0.0315
HTR5A	14	rs2581844	0.0660	-0.1418	0.0655
HTR6	16	rs7522389	0.0060	-0.1100	0.0055
HTR7	22	rs7920627	0.1260	-0.1244	0.1256
IL1B	11	rs4849124	0.1020	-0.0586	0.1020
IL6	16	rs7801617	0.0560	0.1045	0.0555
KCNK2	45	rs2841615	0.1140	0.0531	0.1142
KCTD15	17	rs285676	0.0410	-0.1001	0.0408
LEP	12	rs10487506	0.0340	0.0711	0.0343
LEPR	44	rs10158579	0.0100	-0.1274	0.0104
MAOA	8	rs6609257	0.2480	0.0426	0.2478
MAOB	7	rs6609257	0.2480	0.0426	0.2478
MC4R	12	rs12958350	0.1660	0.0467	0.1660
MTCH2	3	rs1474056	0.6150	0.0236	0.6151
NEGR1	128	rs1426179	0.0090	-0.0999	0.0090
NOS1	48	rs12099598	0.0070	0.1085	0.0073
NR3C1	20	rs4912911	0.0760	-0.0623	0.0757
NTRK2	82	rs7026417	0.0040	0.1646	0.0041
OLIG1	8	rs928736	0.1400	-0.0528	0.1403
OLIG2	14	rs2834072	0.0530	-0.0634	0.0533
OLIG3	14	rs9385796	0.0330	0.1507	0.0329
OPRM1	79	rs10223804	0.0380	0.0955	0.0383
P2RX1	20	rs6502751	0.1190	0.0873	0.1192
P2RX2	7	rs11146967	0.2210	-0.0411	0.2211
P2RX3	11	rs3741089	0.4030	0.0277	0.4032
P2RX4	14	rs10774589	0.0870	-0.0744	0.0868
P2RX5	9	rs3817666	0.2200	0.0986	0.2202
P2RX6	21	rs12627919	0.0090	0.1514	0.0090
P2RX7	25	rs12815078	0.0950	0.0957	0.0954
PCLO	78	rs16887353	0.0150	0.1458	0.0148
PDE11A	104	rs2695109	0.0660	-0.0753	0.0664
PDE1A	79	rs11690832	0.0190	-0.0770	0.0195
PDE9A	56	rs2245730	0.0009	-0.1118	0.0009
PER1	6	rs2253820	0.1640	0.0629	0.1638

PER2	11	rs7579382	0.0690	0.0611	0.0688
PER3	18	rs12566535	0.0300	0.1094	0.0302
PRKCH	80	rs6573391	0.0060	0.1690	0.0062
PSMB4	4	rs1887545	0.4590	0.0324	0.4589
PSMD9	6	rs895959	0.0600	0.0907	0.0596
S100B	2	rs2839362	0.1080	0.0967	0.1083
SH2B1	2	rs4788102	0.0650	0.0620	0.0646
SLC6A1	43	rs11719708	0.0160	0.0799	0.0160
SLC6A2	33	rs187715	0.0050	-0.2086	0.0050
SLC6A3	24	rs11133767	0.0230	-0.0808	0.0232
SLC6A4	14	rs2066713	0.0040	0.0988	0.0035
STAT3	10	rs17405722	0.1880	0.0936	0.1875
TBX21	5	rs7502875	0.0280	0.0880	0.0284
TMEM18	21	rs6728479	0.1110	-0.0929	0.1117
TPH1	8	rs11024449	0.0680	0.0699	0.0677
TPH2	34	rs5019656	0.0760	0.1013	0.0761
UST	134	rs2500535	0.0020	-0.2284	0.0023
ZNF804A	31	rs2369595	0.0140	-0.1023	0.0140

Table 15: Candidate gene results from the analysis between SNP markers and adjusted percentage change in depression severity in 1222 SRI-treated individuals. Only the top associated SNP is shown from each gene. Regression coefficient is standardized and can be interpreted as a measure of effect size: it is the number of standard deviations in outcome per minor allele. Positive values of regression coefficient mean that carriers of more minor alleles had better treatment outcome. Negative values of regression coefficient mean that carriers of more minor alleles had worse outcomes.

		Serotonergic Antidepressant Analysis (n=1222)			
Gene Symbol	Number of SNPs tested	Top SNP	Regression Coefficient	SE	p-Value
ABCB1	59	rs2235074	0.0815	-0.1731	0.0993
ACE	11	rs4459609	0.0013	0.1315	0.0407
ADRA1A	67	rs11779546	0.0042	-0.1446	0.0504
ADRA1B	21	rs6874816	0.0251	-0.1257	0.0560
ADRA2A	8	rs491589	0.1910	0.0729	0.0557
ADRA2B	5	rs749457	0.2389	-0.0492	0.0418
ADRA2C	5	rs4916612	0.0078	-0.1832	0.0687
ADRB1	16	rs740746	0.0124	-0.1122	0.0448
AKT1	4	rs4983387	0.1599	0.0843	0.0599
ANK3	118	rs12355908	0.0232	-0.1061	0.0467
ANKK1	15	rs2734838	0.0524	-0.0817	0.0421
AVPR1A	10	rs17098991	0.0786	0.1206	0.0685
AVPR1B	5	rs28588803	0.7664	0.0198	0.0667
AVPR2	5	rs4898457	0.3582	-0.0471	0.0513
BDNF	14	rs7934165	0.0127	0.0974	0.0390
CACNA1C	191	rs12813847	0.0110	-0.1791	0.0704
CCL28	9	rs779850	0.3308	0.0510	0.0524
CD3E	6	rs2277289	0.0170	-0.1039	0.0435
CLOCK	10	rs9312661	0.0629	-0.0777	0.0417
CNR1	22	rs9353525	0.0251	0.1393	0.0621
COMT	28	rs737866	0.0281	0.0989	0.0450
CREB1	7	rs2709373	0.3883	-0.0447	0.0518
CRHR1	22	rs12373139	0.0777	0.0849	0.0481
CRHR2	19	rs24003	0.1778	-0.0556	0.0412
CXCR4	8	rs12691874	0.3197	0.0401	0.0403
DRD1	23	rs10039221	0.0541	0.0575	0.3467
DRD2	29	rs2734838	0.0524	-0.0817	0.0421
DRD3	23	rs6787134	0.0942	0.0966	0.0577
DRD4	6	rs11603404	0.5193	0.0376	0.0583
DRD5	3	rs1533615	0.1976	0.1231	0.0955
DTNBP1	31	rs13198533	0.0248	-0.1042	0.0464
EDNRB	5	rs9544635	0.0988	-0.1136	0.0688
FGFR1	13	rs328300	0.1735	0.0547	0.0401
FGFR2	33	rs2981451	0.0321	-0.0872	0.0406

FGFR3	1	rs743682	0.3224	-0.0698	0.0705
FGFR4	4	rs451643	0.1443	0.0635	0.0435
FKBP5	22	rs10456432	0.0095	-0.1287	0.0495
FLT1	42	rs9579176	0.0772	0.0863	0.0488
FTO	97	rs11642776	0.0073	-0.2294	0.0853
GNAS	16	rs234623	0.0030	0.1201	0.0404
GNB3	16	rs5446	0.0345	0.0905	0.0427
GNPDA2	2	rs12640665	0.0652	0.1554	0.0842
GRIA1	94	rs11746246	0.1046	0.0716	0.0441
GRIA2	9	rs4691394	0.1014	0.0918	0.0560
GRIA3	60	rs5911623	0.0022	-0.1411	0.0459
GRIA4	57	rs609665	0.0012	-0.1338	0.0412
GRIK1	116	rs2205177	0.0623	-0.1511	0.0810
GRIK2	159	rs6926170	0.0165	0.0968	0.0403
GRIK3	31	rs11801494	0.0017	0.2209	0.0703
GRIK4	107	rs4445646	0.0007	0.1425	0.0422
GRIK5	5	rs4803523	0.2810	-0.0557	0.0517
GRIN1	3	rs4880094	0.0305	-0.1044	0.0482
GRIN2A	147	rs9928984	0.0131	-0.2577	0.1037
GRIN2B	173	rs10845809	0.0667	-0.1168	0.0636
GRIN2C	5	rs3803783	0.1467	0.0647	0.0446
GRIN2D	9	rs275844	0.3410	0.0573	0.0602
GRIN3A	61	rs1415644	0.0124	-0.1191	0.0476
GSK3A	1	rs11878620	0.1134	0.1281	0.0808
GSK3B	24	rs11919783	0.0049	-0.1838	0.0652
HTR1A	2	rs1364043	0.2660	-0.0532	0.0478
HTR1B	13	rs9352483	0.0230	0.1288	0.0566
HTR2A	50	rs4942578	0.0241	0.1203	0.0533
HTR2B	4	rs13394402	0.0962	0.0866	0.0520
HTR2C	15	rs10875535	0.1850	0.1261	0.0951
HTR3A	22	rs10891611	0.0793	0.0964	0.0549
HTR3B	13	rs3891484	0.0418	-0.1150	0.0564
HTR4	41	rs1833704	0.0005	0.1927	0.0552
HTR5A	14	rs2581844	0.0702	-0.1804	0.0996
HTR6	16	rs2314331	0.0436	0.0983	0.0487
HTR7	22	rs11186300	0.3964	-0.0344	0.0405
IL1B	11	rs4849124	0.3125	-0.0441	0.0437
IL6	16	rs10242595	0.0074	0.1134	0.0423
KCNK2	45	rs7528988	0.1110	0.0702	0.0440
KCTD15	17	rs285680	0.0638	0.1308	0.0705
LEP	12	rs791595	0.3213	0.0521	0.0525
LEPR	44	rs10158579	0.0058	-0.1675	0.0606
MAOA	8	rs6609257	0.1689	0.0625	0.0454
MAOB	7	rs6609257	0.1689	0.0625	0.0454

MC4R	12	rs12958350	0.2805	0.0438	0.0406
MTCH2	3	rs1474056	0.5678	0.0324	0.0568
NEGR1	128	rs7520086	0.0276	-0.1369	0.0621
NOS1	48	rs12099598	0.0026	0.1456	0.0483
NR3C1	20	rs10482672	0.1672	-0.0797	0.0577
NTRK2	82	rs11140783	0.0043	-0.1997	0.0698
OLIG1	8	rs2834078	0.0665	-0.1857	0.1011
OLIG2	14	rs2834072	0.1696	-0.0547	0.0398
OLIG3	14	rs9385796	0.0958	0.1445	0.0867
OPRM1	79	rs10223804	0.0386	0.1154	0.0557
P2RX1	20	rs8076383	0.2703	-0.0444	0.0402
P2RX2	7	rs5744990	0.5258	-0.0343	0.0540
P2RX3	11	rs10896605	0.3936	0.0453	0.0530
P2RX4	14	rs3794207	0.1620	-0.0597	0.0427
P2RX5	9	rs3817666	0.0866	0.1717	0.1001
P2RX6	21	rs1548412	0.0401	0.1550	0.0754
P2RX7	25	rs568531	0.1818	0.1010	0.0756
PCLO	78	rs6467917	0.0234	-0.0939	0.0414
PDE11A	104	rs2695109	0.0234	-0.1137	0.0501
PDE1A	79	rs11690832	0.0121	-0.1032	0.0411
PDE9A	56	rs2269143	0.0055	-0.1615	0.0581
PER1	6	rs2253820	0.0958	0.0909	0.0545
PER2	11	rs7579382	0.1774	0.0554	0.0410
PER3	18	rs12566535	0.1659	0.0843	0.0608
PRKCH	80	rs6573391	0.0047	0.2180	0.0770
PSMB4	4	rs1887545	0.1988	0.0678	0.0528
PSMD9	6	rs7137218	0.2302	-0.0474	0.0395
S100B	2	rs2839362	0.2456	0.0862	0.0742
SH2B1	2	rs4788102	0.0070	0.1108	0.0410
SLC6A1	43	rs1710891	0.0480	-0.0823	0.0416
SLC6A2	33	rs187715	0.0263	-0.2036	0.0915
SLC6A3	24	rs6869645	0.0215	-0.1824	0.0792
SLC6A4	14	rs2066713	0.0284	0.0909	0.0414
STAT3	10	rs17405722	0.2673	0.0940	0.0847
TBX21	5	rs7502875	0.0655	0.0932	0.0506
TMEM18	21	rs6728479	0.0319	-0.1505	0.0701
TPH1	8	rs11024449	0.0571	0.0868	0.0456
TPH2	34	rs5019656	0.0376	0.1427	0.0686
UST	134	rs9377172	0.0130	0.1855	0.0746
ZNF804A	31	rs2369595	0.0009	-0.1676	0.0505

Table 16: Candidate gene results from the analysis between SNP makers and adjusted percentage change in depression severity in 568 NRI-treated individuals. Only the top associated SNP is shown from each gene. Regression coefficient is standardized and can be interpreted as a measure of effect size: it is the number of standard deviations in outcome per minor allele. Positive values of regression coefficient mean that carriers of more minor alleles had better treatment outcome. Negative values of regression coefficient mean that carriers of more minor alleles had worse outcomes..

		Noradrenergic Antidepressant Analysis (n=568)			
Gene symbol	Number of SNPs tested	Top SNP	Regression Coefficient	SE	p-Value
ABCB1	59	rs2214102	0.0461	-0.2104	0.0461
ACE	11	rs4353	0.0875	-0.0996	0.0875
ADRA1A	67	rs1442341	0.0045	0.2523	0.0045
ADRA1B	21	rs10515807	0.0750	0.1329	0.0750
ADRA2A	8	rs491589	0.2307	-0.0949	0.2307
ADRA2B	5	rs749457	0.1691	0.0803	0.1691
ADRA2C	5	rs7692883	0.0170	-0.2208	0.0170
ADRB1	16	rs17875474	0.1066	-0.2182	0.1066
AKT1	4	rs4983559	0.2340	-0.0726	0.2340
ANK3	118	rs10509119	0.0401	-0.1834	0.0401
ANKK1	15	rs1055075	0.0700	-0.1116	0.0700
AVPR1A	10	rs7960075	0.0624	0.3246	0.0624
AVPR1B	5	rs28425623	0.2560	0.0995	0.2560
AVPR2	5	rs2269368	0.1720	0.1269	0.1720
BDNF	14	rs10835211	0.1630	-0.0954	0.1630
CACNA1C	191	rs2238044	0.0035	0.1678	0.0035
CCL28	9	rs922439	0.0547	-0.1261	0.0547
CD3E	6	rs4938506	0.0449	-0.1316	0.0449
CLOCK	10	rs11932595	0.0616	0.1093	0.0616
CNR1	22	rs806368	0.0257	0.1590	0.0257
COMT	28	rs165815	0.0155	0.2030	0.0155
CREB1	7	rs2551645	0.2839	0.0653	0.2839
CRHR1	22	rs242942	0.1942	0.1160	0.1942
CRHR2	19	rs255142	0.0996	-0.0968	0.0996
CXCR4	8	rs11688530	0.1403	0.1618	0.1403
DRD1	23	rs265971	0.0271	0.1447	0.0271
DRD2	29	rs1800497	0.2231	-0.0910	0.2231
DRD3	23	rs963468	0.1294	-0.0929	0.1294

DRD4	6	rs11603404	0.0867	-0.1427	0.0867
DRD5	3	rs1519097	0.0916	-0.1045	0.0916
DTNBP1	31	rs9396592	0.0457	0.1199	0.0457
EDNRB	5	rs7994913	0.6643	0.0259	0.6643
FGFR1	13	rs13317	0.0674	0.1248	0.0674
FGFR2	33	rs10510097	0.0591	0.1790	0.0591
FGFR3	1	rs743682	0.3376	-0.0952	0.3376
FGFR4	4	rs244731	0.0966	0.1178	0.0966
FKBP5	22	rs10456432	0.1195	0.1174	0.1195
FLT1	42	rs10507385	0.0116	-0.1574	0.0116
FTO	97	rs4386132	0.0154	-0.3249	0.0154
GNAS	16	rs13042263	0.0373	-0.1257	0.0373
GNB3	16	rs5439	0.0048	-0.2706	0.0048
GNPDA2	2	rs12640665	0.5140	0.0737	0.5140
GRIA1	94	rs1353090	0.0239	0.2584	0.0239
GRIA2	9	rs17035903	0.0137	-0.1792	0.0137
GRIA3	60	rs3761557	0.0196	0.1660	0.0196
GRIA4	57	rs17391295	0.0107	0.2870	0.0107
GRIK1	116	rs363512	0.0003	0.3827	0.0003
GRIK2	159	rs2749056	0.0092	0.2824	0.0092
GRIK3	31	rs3767065	0.0078	0.2461	0.0078
GRIK4	107	rs3781817	0.0067	-0.1565	0.0067
GRIK5	5	rs2217342	0.0800	-0.1774	0.0800
GRIN1	3	rs10870198	0.0792	0.1020	0.0792
GRIN2A	147	rs7188329	0.0028	0.1761	0.0028
GRIN2B	173	rs220590	0.0054	0.1712	0.0054
GRIN2C	5	rs1568447	0.5009	-0.0403	0.5009
GRIN2D	9	rs1799286	0.0537	0.2799	0.0537
GRIN3A	61	rs945870	0.1678	0.1413	0.1678
GSK3A	1	rs11878620	0.6192	-0.0524	0.6192
GSK3B	24	rs11925868	0.2200	0.1019	0.2200
HTR1A	2	rs1364043	0.7489	-0.0218	0.7489
HTR1B	13	rs9352481	0.1686	0.0823	0.1686
HTR2A	50	rs4942578	0.0277	0.1673	0.0277

HTR2B	4	rs13394402	0.0430	0.1588	0.0430
HTR2C	15	rs1414324	0.1164	0.1457	0.1164
HTR3A	22	rs17543669	0.0478	-0.2585	0.0478
HTR3B	13	rs11606194	0.0712	-0.1876	0.0712
HTR4	41	rs2278392	0.0909	0.1546	0.0909
HTR5A	14	rs2581844	0.4416	-0.0938	0.4416
HTR6	16	rs7522389	0.0143	-0.1645	0.0143
HTR7	22	rs10785973	0.0369	0.1285	0.0369
IL1B	11	rs12469600	0.0952	-0.1086	0.0952
IL6	16	rs10242595	0.0913	-0.1069	0.0913
KCNK2	45	rs11802559	0.0900	-0.1251	0.0900
KCTD15	17	rs3810361	0.2135	-0.0741	0.2135
LEP	12	rs10487506	0.0020	0.1802	0.0020
LEPR	44	rs11208659	0.0799	0.1761	0.0799
MAOA	8	rs909525	0.1732	-0.0918	0.1732
MAOB	7	rs3027409	0.2234	-0.2366	0.2234
MC4R	12	rs11872992	0.3479	-0.0779	0.3479
MTCH2	3	rs10838738	0.7756	-0.0178	0.7756
NEGR1	128	rs2221513	0.0010	-0.2588	0.0010
NOS1	48	rs1879417	0.1244	-0.0896	0.1244
NR3C1	20	rs4607376	0.0017	0.1821	0.0017
NTRK2	82	rs2808707	0.0281	0.1331	0.0281
OLIG1	8	rs4817527	0.1559	0.0819	0.1559
OLIG2	14	rs2834076	0.0317	0.1231	0.0317
OLIG3	14	rs10428802	0.0651	0.1281	0.0651
OPRM1	79	rs512053	0.0823	-0.1732	0.0823
P2RX1	20	rs6502751	0.0458	0.2101	0.0458
P2RX2	7	rs11146967	0.0926	-0.0986	0.0926
P2RX3	11	rs490358	0.1339	0.1004	0.1339
P2RX4	14	rs10774589	0.1166	-0.1108	0.1166
P2RX5	9	rs220488	0.1399	-0.1141	0.1399
P2RX6	21	rs12627919	0.0597	0.2000	0.0597

P2RX7	25	rs12815078	0.1200	0.1518	0.1200
PCLO	78	rs7807790	0.0149	0.1698	0.0149
PDE11A	104	rs763757	0.0187	0.3632	0.0187
PDE1A	79	rs833153	0.0093	-0.4345	0.0093
PDE9A	56	rs2245730	0.0495	-0.1147	0.0495
PER1	6	rs2304911	0.2013	-0.1733	0.2013
PER2	11	rs7579382	0.1655	0.0812	0.1655
PER3	18	rs10462018	0.0198	-0.2023	0.0198
PRKCH	80	rs10438143	0.0122	-0.3498	0.0122
PSMB4	4	rs1887545	0.4514	-0.0591	0.4514
PSMD9	6	rs895959	0.0083	0.2321	0.0083
S100B	2	rs2839362	0.2712	0.1133	0.2712
SH2B1	2	rs4788102	0.5052	-0.0387	0.5052
SLC6A1	43	rs1710887	0.0415	0.1238	0.0415
SLC6A2	33	rs36024	0.0594	-0.1095	0.0594
SLC6A3	24	rs464049	0.1563	-0.0832	0.1563
SLC6A4	14	rs1050565	0.0112	0.1551	0.0112
STAT3	10	rs8074524	0.3461	-0.0717	0.3461
TBX21	5	rs7502875	0.2383	0.0781	0.2383
TMEM18	21	rs4854350	0.2148	-0.0783	0.2148
TPH1	8	rs17794760	0.2802	-0.0830	0.2802
TPH2	34	rs12231356	0.2972	-0.1286	0.2972
UST	134	rs2500535	0.0006	-0.4320	0.0006
ZNF804A	31	rs13384546	0.1150	0.1284	0.1150

Table 17: Candidate gene results from the analysis between SNP markers and antidepressant type (SRI vs. NRI) for their effects on the adjusted percentage change in depression severity among the 949 individuals randomly allocated to SRI or NRI antidepressant. Only the top associated SNP is shown from each gene. Genes located on the X-chromosome were not tested. Regression coefficient is standardized and can be interpreted as a measure of effect size: it is the number of standard deviations in outcome per minor allele. Positive values of regression coefficient mean that carriers of more minor alleles had better treatment outcome. Negative values of regression coefficient mean that carriers of more minor alleles had worse outcomes.

Gene symbol	Number of SNPs tested	Gene by Drug Interaction Analysis (n=949)			
		Top SNP	Regression Coefficient	SE	p-Value
ABCB1	59	rs2214102	0.0203	-0.3466	0.0203
ACE	11	rs4311	0.0052	-0.2533	0.0052
ADRA1A	67	rs1472346	0.0247	0.2348	0.0247
ADRA1B	21	rs7718362	0.0083	-0.8444	0.0083
ADRA2A	8	rs491589	0.3466	-0.1152	0.3466
ADRA2B	5	rs749457	0.3433	0.0876	0.3433
ADRA2C	5	rs4916612	0.0359	0.3121	0.0359
ADRB1	16	rs17875474	0.1056	-0.3653	0.1056
AKT1	4	rs2494738	0.4362	0.1327	0.4362
ANK3	118	rs1981251	0.0736	-0.1643	0.0736
ANKK1	15	rs1800497	0.2921	-0.1195	0.2921
AVPR1A	10	rs17098991	0.1485	-0.2234	0.1485
AVPR1B	5	rs28588803	0.4575	0.1125	0.4575
AVPR2					
BDNF	14	rs7934165	0.0964	-0.1471	0.0964
CACNA1C	191	rs17801211	0.0225	0.2146	0.0225
CCL28	9	rs779850	0.1229	0.1784	0.1229
CD3E	6	rs12576947	0.4350	0.0737	0.4350
CLOCK	10	rs1522113	0.0011	0.6128	0.0011
CNR1	22	rs1406977	0.0891	-0.1887	0.0891
COMT	28	rs737866	0.0494	-0.1925	0.0494
CREB1	7	rs2709373	0.0570	0.2157	0.0570
CRHR1	22	rs242942	0.0714	0.2581	0.0714
CRHR2	19	rs255142	0.1517	-0.1309	0.1517
CXCR4	8	rs4954391	0.2769	0.1107	0.2769
DRD1	23	rs265978	0.0476	0.1803	0.0476
DRD2	29	rs11214606	0.2609	0.2505	0.2609
DRD3	23	rs7633291	0.0958	0.1943	0.0958

DRD4	6	rs11603404	0.0085	-0.3356	0.0085
DRD5	3	rs1519097	0.0301	-0.2039	0.0301
DTNBP1	31	rs2072824	0.1152	-0.1729	0.1152
EDNRB	5	rs9544635	0.1857	0.2078	0.1857
FGFR1	13	rs7825208	0.0125	-0.3641	0.0125
FGFR2	33	rs3750817	0.0410	-0.1862	0.0410
FGFR3	1	rs743682	0.1061	-0.2496	0.1061
FGFR4	4	rs451643	0.2176	-0.1202	0.2176
FKBP5	22	rs10456432	0.0274	0.2454	0.0274
FLT1	42	rs9513089	0.0581	0.1768	0.0581
FTO	97	rs11642776	0.0194	0.4594	0.0194
GNAS	16	rs13042263	0.0034	-0.2660	0.0034
GNB3	16	rs10744720	0.0075	0.2806	0.0075
GNPDA2	2	rs12499960	0.3327	-0.1040	0.3327
GRIA1	94	rs11741791	0.0051	0.4859	0.0051
GRIA2	9	rs4691394	0.1962	-0.1697	0.1962
GRIA3					
GRIA4	57	rs2249031	0.0255	-0.4852	0.0255
GRIK1	116	rs363564	0.0310	0.2607	0.0310
GRIK2	159	rs2852507	0.0282	-0.2512	0.0282
GRIK3	31	rs537958	0.0827	-0.1610	0.0827
GRIK4	107	rs6589832	0.0060	-0.2411	0.0060
GRIK5	5	rs454150	0.0451	-0.3017	0.0451
GRIN1	3	rs4880094	0.0049	0.2938	0.0049
GRIN2A	147	rs1550959	0.0462	-0.2814	0.0462
GRIN2B	173	rs2300238	0.0042	-0.2595	0.0042
GRIN2C	5	rs690418	0.2538	-0.1260	0.2538
GRIN2D	9	rs1799286	0.0263	0.5100	0.0263
GRIN3A	61	rs945870	0.0570	0.2994	0.0570
GSK3A	1	rs11878620	0.0827	-0.3126	0.0827
GSK3B	24	rs11919783	0.0145	0.3553	0.0145
HTR1A	2	rs1364043	0.2203	0.1319	0.2203
HTR1B	13	rs1343491	0.0155	-0.3655	0.0155
HTR2A	50	rs9534507	0.0725	0.3925	0.0725
HTR2B	4	rs4973377	0.0399	0.2630	0.0399
HTR2C					

HTR3A	22	rs17543669	0.0289	-0.4484	0.0289
HTR3B	13	rs3891484	0.0205	0.2987	0.0205
HTR4	41	rs888961	0.0045	0.3042	0.0045
HTR5A	14	rs1657280	0.4277	-0.0752	0.4277
HTR6	16	rs9064	0.0347	0.2290	0.0347
HTR7	22	rs1107688	0.0050	-0.3532	0.0050
IL1B	11	rs12469600	0.0129	-0.2589	0.0129
IL6	16	rs2069837	0.0045	-0.5079	0.0045
KCNK2	45	rs1157493	0.1404	0.1430	0.1404
KCTD15	17	rs29942	0.2676	-0.1039	0.2676
LEP	12	rs2021808	0.0737	-0.3387	0.0737
LEPR	44	rs6588147	0.0853	-0.1605	0.0853
MAOA					
MAOB					
MC4R	12	rs17066865	0.2071	-0.4297	0.2071
MTCH2	3	rs10838738	0.3042	-0.0980	0.3042
NEGR1	128	rs6683448	0.0018	-0.2917	0.0018
NOS1	48	rs11068458	0.0348	-0.4231	0.0348
NR3C1	20	rs4607376	0.0094	0.2276	0.0094
NTRK2	82	rs7875184	0.0008	0.4476	0.0008
OLIG1	8	rs2834079	0.0356	-0.2775	0.0356
OLIG2	14	rs6517137	0.0916	-0.2955	0.0916
OLIG3	14	rs1360606	0.1296	0.1764	0.1296
OPRM1	79	rs13196610	0.0130	-0.4193	0.0130
P2RX1	20	rs12451483	0.0701	-0.1760	0.0701
P2RX2	7	rs5744990	0.2065	0.1480	0.2065
P2RX3	11	rs10896605	0.1260	-0.1798	0.1260
P2RX4	14	rs2686386	0.0583	-0.2177	0.0583
P2RX5	9	rs2318104	0.1643	-0.1562	0.1643
P2RX6	21	rs2239961	0.2291	0.1392	0.2291
P2RX7	25	rs2686386	0.0583	-0.2177	0.0583
PCLO	78	rs7807790	0.0269	0.2242	0.0269
PDE11A	104	rs7605757	0.0062	0.3690	0.0062
PDE1A	79	rs833158	0.0127	-0.3178	0.0127
PDE9A	56	rs7279886	0.0354	-0.2011	0.0354
PER1	6	rs2304911	0.1427	-0.3047	0.1427
PER2	11	rs4663868	0.2964	0.1642	0.2964

PER3	18	rs1044245	0.0788	-0.2318	0.0788
PRKCH	80	rs1033908	0.0015	-0.3448	0.0015
PSMB4	4	rs1887545	0.0445	-0.2413	0.0445
PSMD9	6	rs4069666	0.0058	-0.2703	0.0058
S100B	2	rs2839357	0.7700	-0.0436	0.7700
SH2B1	2	rs4788102	0.0972	-0.1494	0.0972
SLC6A1	43	rs1710887	0.0107	0.2404	0.0107
SLC6A2	33	rs3785143	0.0327	-0.3324	0.0327
SLC6A3	24	rs27072	0.0623	-0.2067	0.0623
SLC6A4	14	rs7214248	0.2258	0.1111	0.2258
STAT3	10	rs4796649	0.3194	0.1592	0.3194
TBX21	5	rs16946264	0.0443	-0.3005	0.0443
TMEM18	21	rs1879524	0.0040	0.3484	0.0040
TPH1	8	rs172424	0.3090	-0.0928	0.3090
TPH2	34	rs1843809	0.0556	-0.2456	0.0556
UST	134	rs9386240	0.0059	-0.3054	0.0059
ZNF804A	31	rs12477430	0.1564	0.1391	0.1564

8 Imputation results from NEWMEDS

Imputation was undertaken in the sample to acquire information about non-genotyped SNPs which may have more statistical evidence for association to outcome than genotyped markers. BEAGLE 3.3 [62] was used for imputing and the HapMap phase 3 CEU population was the reference dataset. This resulted in a dataset of 1.4 million markers. Analyses were conducted on dosage data with estimated probability of each genotype, in order to consider the uncertainty of the imputation. The accuracy of imputation is reported for each result. Imputed data was analysed for association to outcome using the *dosage* command in PLINK [13] in any antidepressant taken (n=1790), in SRI individuals only (n=1222) and in NRI individuals only (n=568). Imputation analyses also included four covariates from EIGENSTRAT to correct for population stratification, as was done in the original analyses.

Tables 18 through 20 show the results from the analysis of the imputed data on response to any antidepressant (Table 18), response to a serotonergic antidepressant (Table 19) and response to a noradrenergic antidepressant (Table 20). Figures 15 to 25 are regional association plots for SNPs with p-values below the genome wide suggestive level of $5*10^{-6}$ from the imputation analysis.

Table 18: Imputed results for the analysis of response to any antidepressant in the entire sample (n=1790) under an additive genetic model. Results are shown for SNPs below the genome-wide suggestive level of evidence of $p<5*10^{-6}$. P-values for SNPs which were genotyped directly are also shown. Regression coefficient is standardized and can be interpreted as a measure of effect size: it is the number of standard deviations in outcome per minor allele. Positive values of regression coefficient mean that carriers of more minor alleles had better treatment outcome. Negative values of regression coefficient mean that carriers of more minor alleles had worse outcomes. Imputation Accuracy's close to 1 indicate the imputation was robust for that SNP. Results from SNPs with an Imputation Accuracy below 0.8 should be taken with caution.

CHR	SNP	Position	GENE	Allele	Imputation Accuracy	Regression Coefficient	SE	p-value	Genotyped	Genotype p-value
6	rs10499161	129954492	*ARHGAP18*	A	0.99	0.23	0.05	4.01E-06	N	
9	rs10818702	124285545	*OR1J2*	C	1.00	0.19	0.04	2.39E-06	Y	2.19E-06
11	rs10832840	1354473	*BRSK2/ PEN11B*	C	1.02	0.20	0.04	4.68E-06	N	
11	rs4881745	1354892	*BRSK2/ PEN11B*	C	0.96	0.21	0.04	3.70E-06	N	
14	rs11624702	46588187	*MDGA2*	C	1.02	-0.15	0.03	4.08E-06	Y	4.08E-06

Table 19: Imputed results for the analysis of response to SRI antidepressant (n=1222) under an additive genetic model. Results are shown for SNPs below the genome-wide suggestive level of evidence of p<5*10-6. P-values for SNPs which were genotyped directly are also shown. Regression coefficient is standardized and can be interpreted as a measure of effect size: it is the number of standard deviations in outcome per minor allele. Positive values of regression coefficient mean that carriers of more minor alleles had better treatment outcome. Negative values of regression coefficient mean that carriers of more minor alleles had worse outcomes. Imputation Accuracy's close to 1 indicate the imputation was robust for that SNP. Results from SNPs with an Imputation Accuracy below 0.8 should be taken with caution.

CHR	SNP	Position	GENE	Allele	Imputation Accuracy	Regression Coefficient	SE	p-value	Genotyped	Genotype p-value
5	rs9291836	64716023	*ADAMTS6*	C	0.98	0.19	0.04	2.00E-06	N	
5	rs7708972	64724497	*ADAMTS6*	A	1.04	0.19	0.04	1.90E-06	Y	2.49E-06
5	rs10042191	64733622	*ADAMTS6*	C	0.98	0.20	0.04	1.03E-06	N	
5	rs10069691	64742796	*ADAMTS6*	A	1.01	0.19	0.04	1.43E-06	N	
5	rs9291837	64743875	*ADAMTS6*	C	1.01	0.19	0.04	2.12E-06	N	
5	rs10072030	64761280	*ADAMTS6*	A	1.01	-0.19	0.04	2.33E-06	N	
5	rs1493451	64762196	*ADAMTS6*	C	1.01	-0.19	0.04	2.35E-06	Y	2.35E-06
5	rs6449784	64766607	*ADAMTS6*	C	1.00	0.19	0.04	2.05E-06	N	
5	rs2047064	64769683	*ADAMTS6*	A	1.01	-0.19	0.04	2.04E-06	N	
5	rs7728131	64770458	*ADAMTS6*	A	0.97	0.19	0.04	2.16E-06	N	
5	rs6876189	64771296	*ADAMTS6*	A	0.97	0.19	0.04	1.80E-06	N	
5	rs7705423	64776521	*ADAMTS6*	A	0.96	0.20	0.04	1.74E-06	N	
5	rs2131587	64781681	*ADAMTS6*	C	0.95	-0.20	0.04	1.53E-06	N	
5	rs7725105	64785479	*ADAMTS6*	A	0.96	0.20	0.04	1.71E-06	N	
5	rs10515893	164692074		A	1.04	-0.21	0.04	1.37E-06	Y	1.37E-06
12	rs10783282	47433214	*ADCY6*	A	0.95	-0.24	0.05	1.16E-06	Y	1.16E-06

Table 20: Imputed results for the analysis of response to NRI antidepressant (n=568) under an additive genetic model. Results are shown for SNPs below the genome-wide suggestive level of evidence of p<5*10-6. P-values for SNPs which were genotyped directly are also shown. Regression coefficient is standardized and can be interpreted as a measure of effect size: it is the number of standard deviations in outcome per minor allele. Positive values of regression coefficient mean that carriers of more minor alleles had better treatment outcome. Negative values of regression coefficient mean that carriers of more minor alleles had worse outcomes. Imputation Accuracy's close to 1 indicate the imputation was robust for that SNP. Results from SNPs with an Imputation Accuracy below 0.8 should be taken with caution.

CHR	SNP	Position	GENE	Allele	Imputation Accuracy	Regression Coefficient	SE	p-value	Genotyped	Genotype p-value
1	rs2988738	227427128		C	0.76	-1.30	0.28	4.48E-06	N	
5	rs2888109	66312784	*MAST4*	G	0.15	-0.91	0.20	4.67E-06	N	
5	rs16875947	78136556	*ARSB*	A	0.99	-0.38	0.08	9.80E-07	N	
5	rs7711802	78138239	*ARSB*	A	1.00	0.37	0.08	1.05E-06	N	
5	rs16876279	78139361	*ARSB*	C	1.00	-0.37	0.08	1.05E-06	N	
7	rs13237776	27676198	*HIBADH*	C	1.02	0.34	0.07	1.77E-06	Y	1.77E-06
7	rs12534474	27677080	*HIBADH*	A	1.02	-0.34	0.07	1.77E-06	Y	1.77E-06

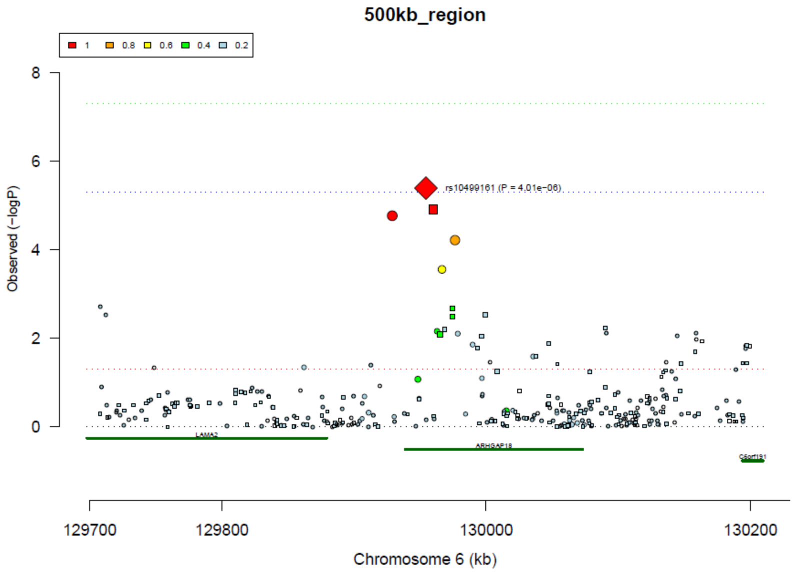

Figure 15: Chromosome 6 associated region with response to any antidepressant treatment (n=1790). Negative decadic logarithm of the uncorrected p-values is plotted against chromosomal location of genotyped (circles) and imputed (squares) SNP markers. The graph covers 500kbp upstream and downstream from the strongest associated imputed marker (rs10499161). Colouring reflects the linkage disequilibrium (LD) between rs10499161 and other SNP markers, with red meaning strong LD and blue weak LD. The red dotted line is p=0.05, the blue dotted line is $p=5*10^{-6}$ and the green dotted line is $p=5*10^{-8}$.

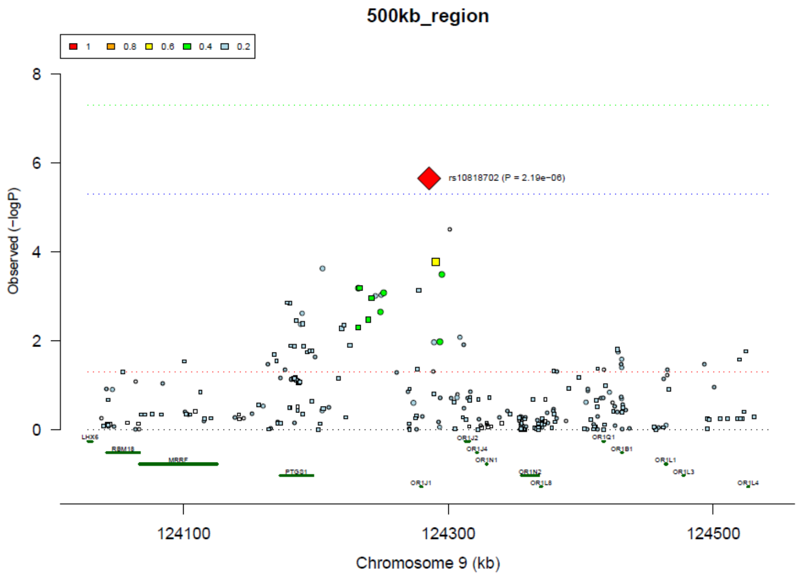

Figure 16: Chromosome 9 associated region with response to any antidepressant treatment (n=1790). Negative decadic logarithm of the uncorrected p-values is plotted against chromosomal location of genotyped (circles) and imputed (squares) SNP markers. The graph covers 500kbp upstream and downstream from the strongest associated genotyped marker (rs10818702). Colouring reflects the linkage disequilibrium (LD) between rs10818702 and other SNP markers, with red meaning strong LD and blue weak LD. The red dotted line is p=0.05, the blue dotted line is $p=5*10^{-6}$ and the green dotted line is $p=5*10^{-8}$.

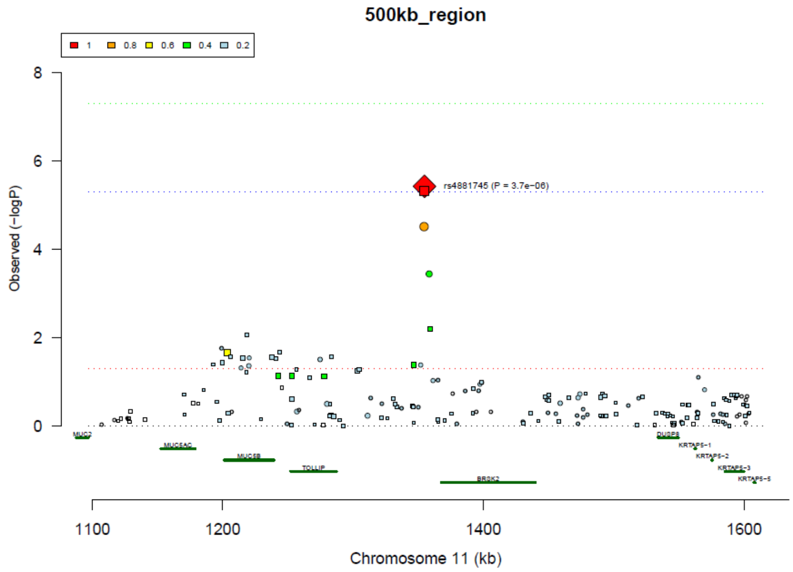

Figure 17: Chromosome 11 associated region with response to any antidepressant treatment (n=1790). Negative decadic logarithm of the uncorrected p-values is plotted against chromosomal location of genotyped (circles) and imputed (squares) SNP markers. The graph covers 500kbp upstream and downstream from the strongest associated imputed marker (rs4881745). Colouring reflects the linkage disequilibrium (LD) between rs4881745 and other SNP markers, with red meaning strong LD and blue weak LD. The red dotted line is p=0.05, the blue dotted line is $p=5*10^{-6}$ and the green dotted line is $p=5*10^{-8}$.

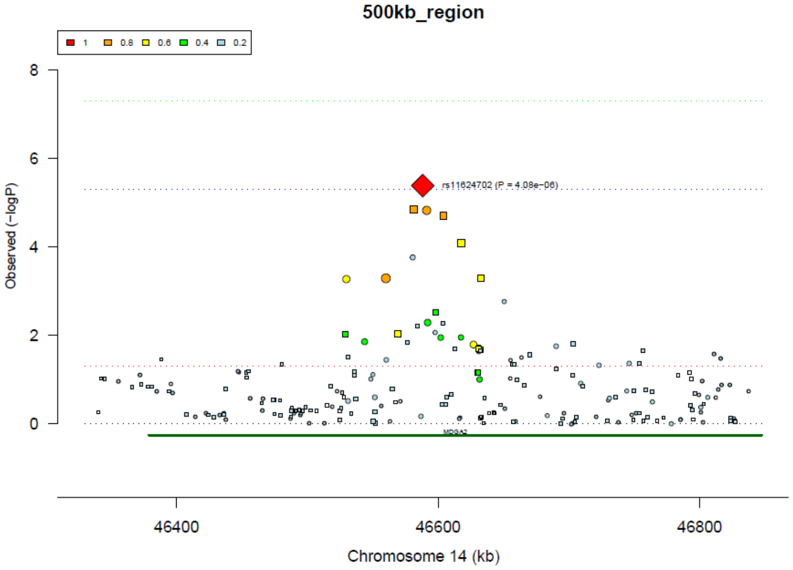

Figure 18: Chromosome 14 associated region with response to any antidepressant treatment (n=1790). Negative decadic logarithm of the uncorrected p-values is plotted against chromosomal location of genotyped (circles) and imputed (squares) SNP markers. The graph covers 500kbp upstream and downstream from the strongest associated genotyped marker (rs11624702). Colouring reflects the linkage disequilibrium (LD) between rs11624702 and other SNP markers, with red meaning strong LD and blue weak LD. The red dotted line is p=0.05, the blue dotted line is p=5*10^{-6} and the green dotted line is p=5*10^{-8}.

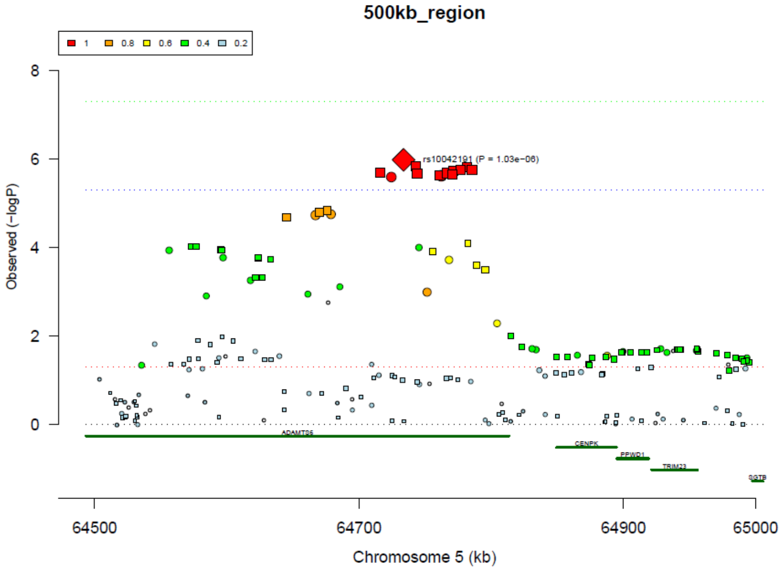

Figure 19: Chromosome 5 associated region with response to serotonergic antidepressant treatment (n=1222). Negative decadic logarithm of the uncorrected p-values is plotted against chromosomal location of genotyped (circles) and imputed (squares) SNP markers. The graph covers 500kbp upstream and downstream from the strongest associated imputed marker (rs10042191). Colouring reflects the linkage disequilibrium (LD) between rs10042191 and other SNP markers, with red meaning strong LD and blue weak LD. The red dotted line is p=0.05, the blue dotted line is p=5*10^{-6} and the green dotted line is p=5*10^{-8}.

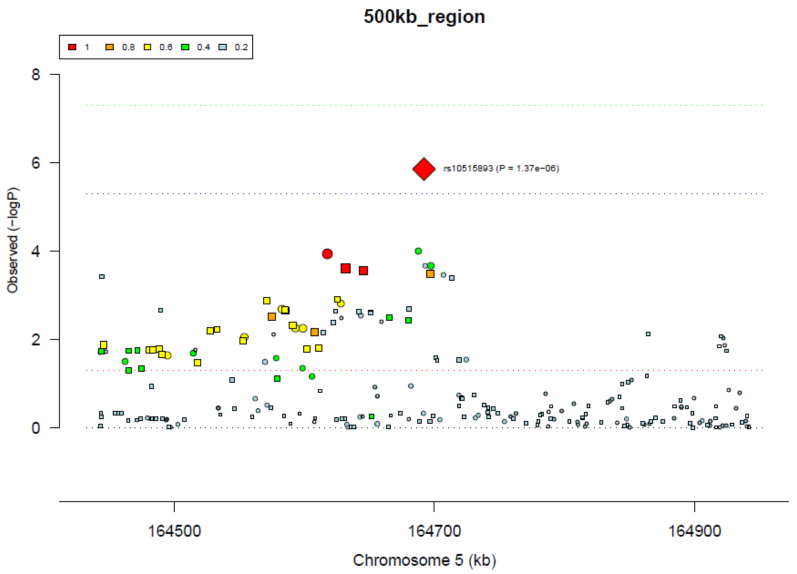

Figure 20: Chromosome 5 associated region with response to serotonergic antidepressant treatment (n=1222). Negative decadic logarithm of the uncorrected p-values is plotted against chromosomal location of genotyped (circles) and imputed (squares) SNP markers. The graph covers 500kbp upstream and downstream from the strongest associated genotyped marker (rs10515893). Colouring reflects the linkage disequilibrium (LD) between rs10515893 and other SNP markers, with red meaning strong LD and blue weak LD. The red dotted line is p=0.05, the blue dotted line is p=5*10^{-6} and the green dotted line is p=5*10^{-8}.

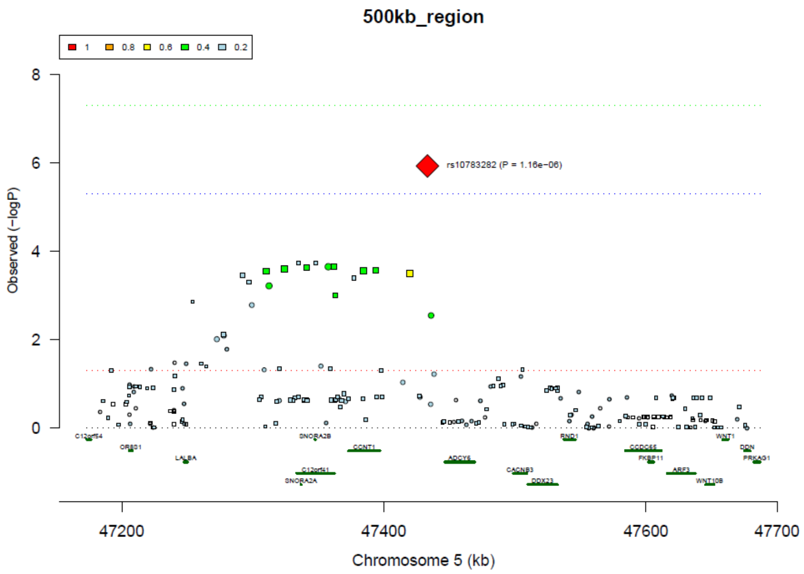

Figure 21: Chromosome 12 associated region with response to serotonergic antidepressant treatment (n=1222). Negative decadic logarithm of the uncorrected p-values is plotted against chromosomal location of genotyped (circles) and imputed (squares) SNP markers. The graph covers 500kbp upstream and downstream from the strongest associated genotyped marker (rs10515893). Colouring reflects the linkage disequilibrium (LD) between rs10515893 and other SNP markers, with red meaning strong LD and blue weak LD. The red dotted line is p=0.05, the blue dotted line is p=5*10^{-6} and the green dotted line is p=5*10^{-8}.

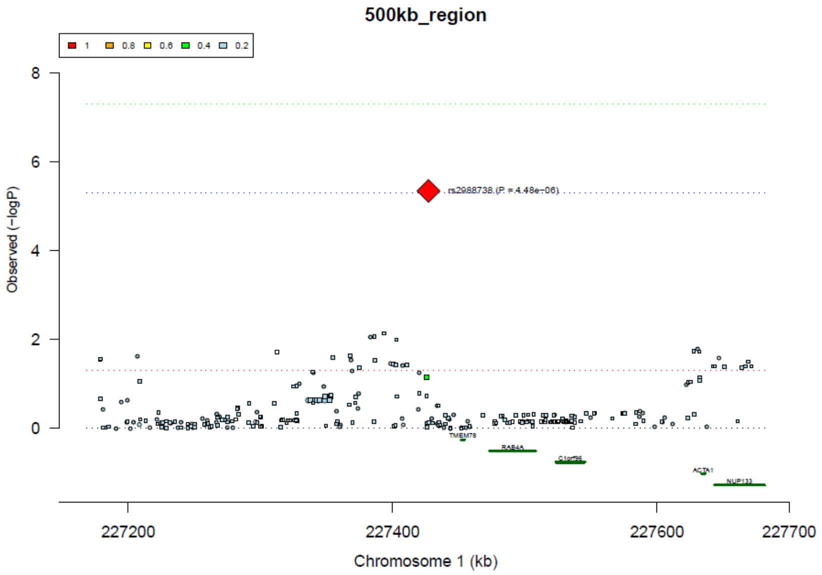

Figure 22: Chromosome 1 associated region with response to noradrenergic antidepressant treatment (n=568). Negative decadic logarithm of the uncorrected p-values is plotted against chromosomal location of genotyped (circles) and imputed (squares) SNP markers. The graph covers 500kbp upstream and downstream from the strongest associated imputed marker (rs2988738). Colouring reflects the linkage disequilibrium (LD) between rs2988738 and other SNP markers, with red meaning strong LD and blue weak LD. The red dotted line is p=0.05, the blue dotted line is $p=5*10^{-6}$ and the green dotted line is $p=5*10^{-8}$.

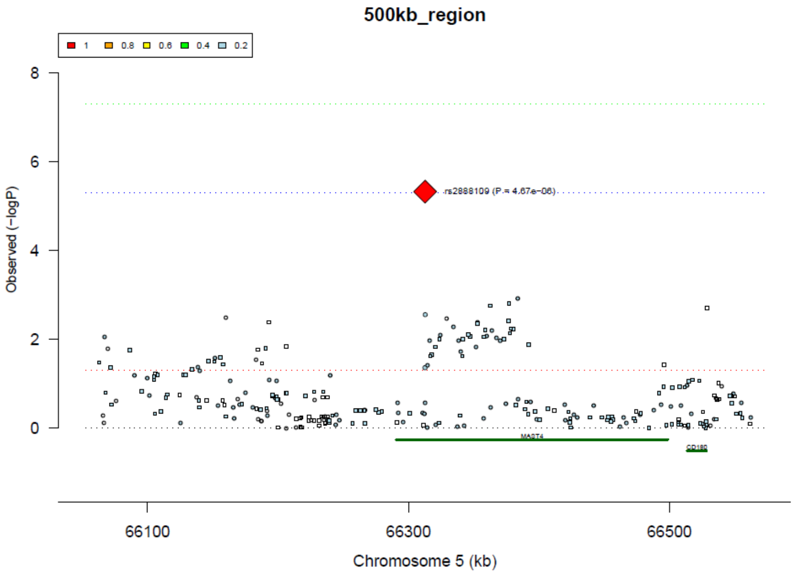

Figure 23: Chromosome 5 associated region with response to noradrenergic antidepressant treatment (n=568). Negative decadic logarithm of the uncorrected p-values is plotted against chromosomal location of genotyped (circles) and imputed (squares) SNP markers. The graph covers 500kbp upstream and downstream from the strongest associated imputed marker (rs2888109). Colouring reflects the linkage disequilibrium (LD) between rs2888109 and other SNP markers, with red meaning strong LD and blue weak LD. The red dotted line is p=0.05, the blue dotted line is $p=5*10^{-6}$ and the green dotted line is $p=5*10^{-8}$.

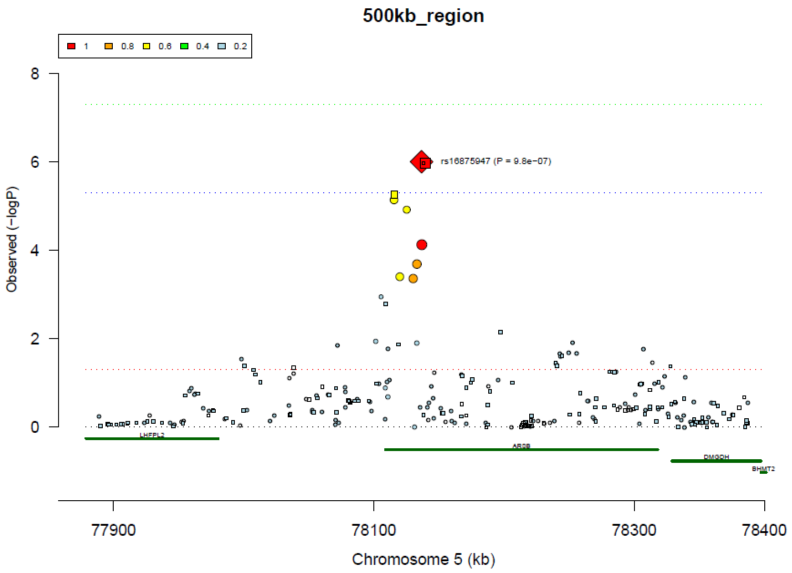

Figure 24: Chromosome 5 associated region with response to noradrenergic antidepressant treatment (n=568). Negative decadic logarithm of the uncorrected p-values is plotted against chromosomal location of genotyped (circles) and imputed (squares) SNP markers. The graph covers 500kbp upstream and downstream from the strongest associated imputed marker (rs16876279). Colouring reflects the linkage disequilibrium (LD) between rs16876279 and other SNP markers, with red meaning strong LD and blue weak LD. The red dotted line is p=0.05, the blue dotted line is $p=5*10^{-6}$ and the green dotted line is $p=5*10^{-8}$.

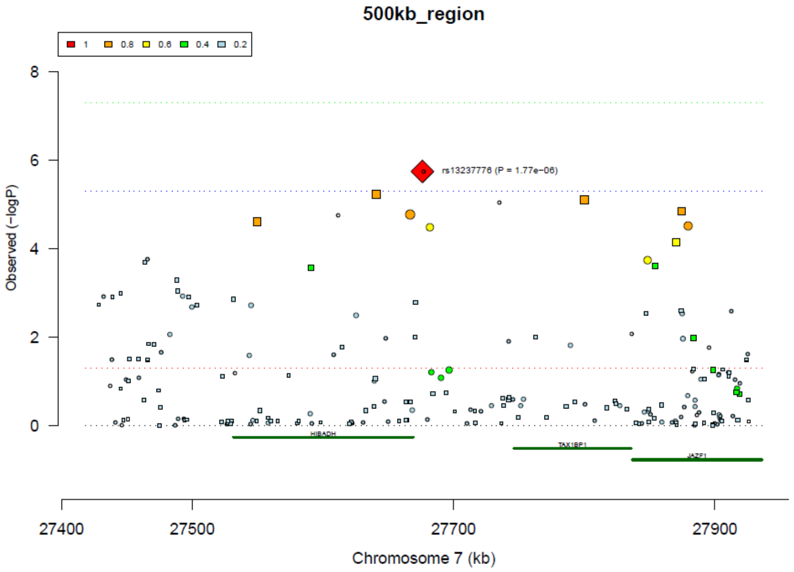

Figure 25: Chromosome 7 associated region with response to noradrenergic antidepressant treatment (n=568). Negative decadic logarithm of the uncorrected p-values is plotted against chromosomal location of genotyped (circles) and imputed (squares) SNP markers. The graph covers 500kbp upstream and downstream from the strongest associated genotyped marker (rs13237776). Colouring reflects the linkage disequilibrium (LD) between rs13237776 and other SNP markers, with red meaning strong LD and blue weak LD. The red dotted line is p=0.05, the blue dotted line is $p=5*10^{-6}$ and the green dotted line is $p=5*10^{-8}$.

9 References:

1. Uher R, Huezo-Diaz P, Perroud N, Smith R, Rietschel M, et al. (2009) Genetic predictors of response to antidepressants in the GENDEP project. Pharmacogenomics J 9: 225-233.
2. Wing JK, Sartorius N, Ustin TB (1998) Diagnosis and Clinical Measurement in Psychiatry: A Reference Manual for SCAN. . Geneva: World Health Organization.
3. Uher R, Farmer A, Maier W, Rietschel M, Hauser J, et al. (2008) Measuring depression: comparison and integration of three scales in the GENDEP study. Psychol Med 38: 289-300.
4. Sanchez C, Bergqvist PB, Brennum LT, Gupta S, Hogg S, et al. (2003) Escitalopram, the S-(+)-enantiomer of citalopram, is a selective serotonin reuptake inhibitor with potent effects in animal models predictive of antidepressant and anxiolytic activities. Psychopharmacology (Berl) 167: 353-362.
5. Sanchez C, Hyttel J (1999) Comparison of the effects of antidepressants and their metabolites on reuptake of biogenic amines and on receptor binding. Cell Mol Neurobiol 19: 467-489.
6. Thomas L, Mulligan J, Mason V, Tallon D, Wiles N, et al. (2008) GENetic and clinical predictors of treatment response in depression: the GenPod randomised trial protocol. Trials 9: 29.
7. Lewis G, Pelosi AJ, Araya R, Dunn G (1992) Measuring psychiatric disorder in the community: a standardized assessment for use by lay interviewers. Psychol Med 22: 465-486.
8. Beck AT, Ward CH, Mendelson M, Mock J, Erbaugh J (1961) An inventory for measuring depression. Arch Gen Psychiatry 4: 561-571.
9. Bondolfi G, Aubry JM, Golaz J, Gex-Fabry M, Gervasoni N, et al. (2006) A stepwise drug treatment algorithm to obtain complete remission in depression: a Geneva study. Swiss Med Wkly 136: 78-85.
10. Perroud N, Bondolfi G, Uher R, Gex-Fabry M, Aubry JM, et al. (2011) Clinical and genetic correlates of suicidal ideation during antidepressant treatment in a depressed outpatient sample. Pharmacogenomics 12: 365-377.
11. Sheehan G (1998) Comment: how well does a concept travel? Aust N Z J Psychiatry 32: 398-399.
12. Clayton AH, Croft HA, Horrigan JP, Wightman DS, Krishen A, et al. (2006) Bupropion extended release compared with escitalopram: effects on sexual functioning and antidepressant efficacy in 2 randomized, double-blind, placebo-controlled studies. J Clin Psychiatry 67: 736-746.
13. Purcell S, Neale B, Todd-Brown K, Thomas L, Ferreira MA, et al. (2007) PLINK: a tool set for whole-genome association and population-based linkage analyses. Am J Hum Genet 81: 559-575.
14. Wittke-Thompson JK, Pluzhnikov A, Cox NJ (2005) Rational inferences about departures from Hardy-Weinberg equilibrium. Am J Hum Genet 76: 967-986.
15. Seldin MF, Shigeta R, Villoslada P, Selmi C, Tuomilehto J, et al. (2006) European population substructure: clustering of northern and southern populations. PLoS Genet 2: e143.

16. Price AL, Patterson NJ, Plenge RM, Weinblatt ME, Shadick NA, et al. (2006) Principal components analysis corrects for stratification in genome-wide association studies. Nat Genet 38: 904-909.
17. Hamilton M (1960) A rating scale for depression. J Neurol Neurosurg Psychiatry 23: 56-62.
18. Montgomery SA, Asberg M (1979) A new depression scale designed to be sensitive to change. Br J Psychiatry 134: 382-389.
19. Royston P, Altman DG, Sauerbrei W (2006) Dichotomizing continuous predictors in multiple regression: a bad idea. Stat Med 25: 127-141.
20. Streiner DL (2002) Breaking up is hard to do: the heartbreak of dichotomizing continuous data. Can J Psychiatry 47: 262-266.
21. Uher R, Muthen B, Souery D, Mors O, Jaracz J, et al. (2010) Trajectories of change in depression severity during treatment with antidepressants. Psychol Med 40: 1367-1377.
22. Uher R, Perroud N, Ng MY, Hauser J, Henigsberg N, et al. (2010) Genome-wide pharmacogenetics of antidepressant response in the GENDEP project. Am J Psychiatry 167: 555-564.
23. Uher R, Maier W, Hauser J, Marusic A, Schmael C, et al. (2009) Differential efficacy of escitalopram and nortriptyline on dimensional measures of depression. Br J Psychiatry 194: 252-259.
24. Leucht S, Kane JM, Etschel E, Kissling W, Hamann J, et al. (2006) Linking the PANSS, BPRS, and CGI: clinical implications. Neuropsychopharmacology 31: 2318-2325.
25. Lane P (2008) Handling drop-out in longitudinal clinical trials: a comparison of the LOCF and MMRM approaches. Pharm Stat 7: 93-106.
26. Mallinckrodt CH, Clark WS, David SR (2001) Accounting for dropout bias using mixed-effects models. J Biopharm Stat 11: 9-21.
27. Uher R, Tansey KE, Malki K, Perlis RH (2012) Biomarkers predicting treatment outcome in depression: what is clinically significant? Pharmacogenomics 13: 233-240.
28. Excellence NIfC (2004) Depression: Management of Depression in Primary and Secondary Care. London.
29. Purcell S, Cherny SS, Sham PC (2003) Genetic Power Calculator: design of linkage and association genetic mapping studies of complex traits. Bioinformatics 19: 149-150.
30. Lin DY, Zeng D (2010) Meta-analysis of genome-wide association studies: no efficiency gain in using individual participant data. Genet Epidemiol 34: 60-66.
31. Spencer CC, Su Z, Donnelly P, Marchini J (2009) Designing genome-wide association studies: sample size, power, imputation, and the choice of genotyping chip. PLoS Genet 5: e1000477.
32. Hellquist A, Zucchelli M, Lindgren CM, Saarialho-Kere U, Jarvinen TM, et al. (2009) Identification of MAMDC1 as a candidate susceptibility gene for systemic lupus erythematosus (SLE). PLoS One 4: e8037.
33. Heck A, Pfister H, Czamara D, Muller-Myhsok B, Putz B, et al. Evidence for associations between MDGA2 polymorphisms and harm avoidance: replication and extension of a genome-wide association finding. Psychiatr Genet 21: 257-260.

34. Hettema JM, van den Oord EJ, An SS, Kendler KS, Chen X (2009) Follow-up association study of novel neuroticism gene MAMDC1. Psychiatr Genet 19: 213-214.
35. van den Oord EJ, Kuo PH, Hartmann AM, Webb BT, Moller HJ, et al. (2008) Genomewide association analysis followed by a replication study implicates a novel candidate gene for neuroticism. Arch Gen Psychiatry 65: 1062-1071.
36. Kendler KS, Gatz M, Gardner CO, Pedersen NL (2006) Personality and major depression: a Swedish longitudinal, population-based twin study. Arch Gen Psychiatry 63: 1113-1120.
37. Kendler KS, Myers J (2010) The genetic and environmental relationship between major depression and the five-factor model of personality. Psychol Med 40: 801-806.
38. Xu S, Witmer PD, Lumayag S, Kovacs B, Valle D (2007) MicroRNA (miRNA) transcriptome of mouse retina and identification of a sensory organ-specific miRNA cluster. J Biol Chem 282: 25053-25066.
39. Saus E, Soria V, Escaramis G, Vivarelli F, Crespo JM, et al. (2010) Genetic variants and abnormal processing of pre-miR-182, a circadian clock modulator, in major depression patients with late insomnia. Hum Mol Genet 19: 4017-4025.
40. Wray NR, Pergadia ML, Blackwood DH, Penninx BW, Gordon SD, et al. (2010) Genome-wide association study of major depressive disorder: new results, meta-analysis, and lessons learned. Mol Psychiatry.
41. Hines LM, Tabakoff B (2005) Platelet adenylyl cyclase activity: a biological marker for major depression and recent drug use. Biol Psychiatry 58: 955-962.
42. Bevitt DJ, Li Z, Lindrop JL, Barker MD, Clarke MP, et al. (2005) Analysis of full length ADAMTS6 transcript reveals alternative splicing and a role for the 5' untranslated region in translational control. Gene 359: 99-110.
43. Angata K, Suzuki M, McAuliffe J, Ding Y, Hindsgaul O, et al. (2000) Differential biosynthesis of polysialic acid on neural cell adhesion molecule (NCAM) and oligosaccharide acceptors by three distinct alpha 2,8-sialyltransferases, ST8Sia IV (PST), ST8Sia II (STX), and ST8Sia III. J Biol Chem 275: 18594-18601.
44. Aonurm-Helm A, Jurgenson M, Zharkovsky T, Sonn K, Berezin V, et al. (2008) Depression-like behaviour in neural cell adhesion molecule (NCAM)-deficient mice and its reversal by an NCAM-derived peptide, FGL. Eur J Neurosci 28: 1618-1628.
45. Beecken WD, Engl T, Ogbomo H, Relja B, Cinatl J, et al. (2005) Valproic acid modulates NCAM polysialylation and polysialyltransferase mRNA expression in human tumor cells. Int Immunopharmacol 5: 757-769.
46. Holmans P, Weissman MM, Zubenko GS, Scheftner WA, Crowe RR, et al. (2007) Genetics of recurrent early-onset major depression (GenRED): final genome scan report. Am J Psychiatry 164: 248-258.
47. Lee MT, Chen CH, Lee CS, Chen CC, Chong MY, et al. (2010) Genome-wide association study of bipolar I disorder in the Han Chinese population. Mol Psychiatry.
48. Arai M, Yamada K, Toyota T, Obata N, Haga S, et al. (2006) Association between polymorphisms in the promoter region of the sialyltransferase 8B (SIAT8B) gene and schizophrenia. Biol Psychiatry 59: 652-659.
49. Tao R, Li C, Zheng Y, Qin W, Zhang J, et al. (2007) Positive association between SIAT8B and schizophrenia in the Chinese Han population. Schizophr Res 90: 108-114.

50. Ashburner M, Ball CA, Blake JA, Botstein D, Butler H, et al. (2000) Gene ontology: tool for the unification of biology. The Gene Ontology Consortium. Nat Genet 25: 25-29.
51. Bult CJ, Eppig JT, Kadin JA, Richardson JE, Blake JA (2008) The Mouse Genome Database (MGD): mouse biology and model systems. Nucleic Acids Res 36: D724-728.
52. Mi H, Dong Q, Muruganujan A, Gaudet P, Lewis S, et al. PANTHER version 7: improved phylogenetic trees, orthologs and collaboration with the Gene Ontology Consortium. Nucleic Acids Res 38: D204-210.
53. Subramanian A, Tamayo P, Mootha VK, Mukherjee S, Ebert BL, et al. (2005) Gene set enrichment analysis: a knowledge-based approach for interpreting genome-wide expression profiles. Proc Natl Acad Sci U S A 102: 15545-15550.
54. Holmans P, Green EK, Pahwa JS, Ferreira MA, Purcell SM, et al. (2009) Gene ontology analysis of GWA study data sets provides insights into the biology of bipolar disorder. Am J Hum Genet 85: 13-24.
55. Stergiakouli E, Hamshere M, Holmans P, Langley K, Zaharieva I, et al. Investigating the Contribution of Common Genetic Variants to the Risk and Pathogenesis of ADHD. Am J Psychiatry.
56. Sklar P, Ripke S, Scott LJ, Andreassen OA, Cichon S, et al. (2011) Large-scale genome-wide association analysis of bipolar disorder identifies a new susceptibility locus near ODZ4. Nat Genet 43: 977-983.
57. Ripke S, Sanders AR, Kendler KS, Levinson DF, Sklar P, et al. (2011) Genome-wide association study identifies five new schizophrenia loci. Nat Genet 43: 969-976.
58. Rush AJ, Fava M, Wisniewski SR, Lavori PW, Trivedi MH, et al. (2004) Sequenced treatment alternatives to relieve depression (STAR*D): rationale and design. Control Clin Trials 25: 119-142.
59. Trivedi MH, Rush AJ, Wisniewski SR, Nierenberg AA, Warden D, et al. (2006) Evaluation of outcomes with citalopram for depression using measurement-based care in STAR*D: implications for clinical practice. Am J Psychiatry 163: 28-40.
60. Garriock HA, Kraft JB, Shyn SI, Peters EJ, Yokoyama JS, et al. (2010) A genomewide association study of citalopram response in major depressive disorder. Biol Psychiatry 67: 133-138.
61. Wellcome Trust Case Control Consortium (2007) Genome-wide association study of 14,000 cases of seven common diseases and 3,000 shared controls. Nature 447: 661-678.
62. Browning SR, Browning BL (2007) Rapid and accurate haplotype phasing and missing-data inference for whole-genome association studies by use of localized haplotype clustering. Am J Hum Genet 81: 1084-1097.
63. Purcell SM, Wray NR, Stone JL, Visscher PM, O'Donovan MC, et al. (2009) Common polygenic variation contributes to risk of schizophrenia and bipolar disorder. Nature 460: 748-752.
64. Ruderfer DM, Kirov G, Chambert K, Moran JL, Owen MJ, et al. (2011) A family-based study of common polygenic variation and risk of schizophrenia. Mol Psychiatry 16: 887-888.
65. Kato M, Serretti A (2010) Review and meta-analysis of antidepressant pharmacogenetic findings in major depressive disorder. Mol Psychiatry 15: 473-500.

66. Horstmann S, Binder EB (2009) Pharmacogenomics of antidepressant drugs. Pharmacol Ther 124: 57-73.
67. Ising M, Lucae S, Binder EB, Bettecken T, Uhr M, et al. (2009) A genomewide association study points to multiple loci that predict antidepressant drug treatment outcome in depression. Arch Gen Psychiatry 66: 966-975.
