# Supplementary material for: Genetic Predictors of Response to Serotonergic and Noradrenergic Antidepressants in Major Depressive Disorder: A Genome-Wide Analysis of Individual-Level Data and a Meta-Analysis
Source: PLoS Med. 2012 Oct 16;9(10):e1001326. doi: 10.1371/journal.pmed.1001326 (PMC3472989; doi:10.1371/journal.pmed.1001326)
Supplement: Text S2 — Study protocol. (PDF) [file pmed.1001326.s002.pdf]

Genome-wide analysis of NEWMEDS (including GENDEP and GenPod studies) 14th April 2010

The following is a plan for pharmacogenetic genome-wide analyses of data on antidepressant treatment in the NEWMEDS project, work package 8. The main aim of the work package in the first 18 months is to establish genetic markers that consistently predict response to serotonergic or noradrenergic antidepressants across different samples, and therefore may be used for defining new drug targets and inform personalised prescribing. To achieve this we bring together samples with a good outcome data for treatment with either serotonergic antidepressants (citalopram, escitalopram, fluoxetine, sertraline, paroxetine) or noradrenergic antidepressants (nortriptyline, reboxetine). As we are unlikely to get sufficiently large samples of individuals of other ethnicities, we will focus on white Europeans.

The NEWMEDS work package 8 combines samples from two academic studies (GENDEP,[1, 2] led by King's College London and GenPod[3] led by the Universities of Bristol and Cardiff) and samples made available by pharmaceutical companies members of EFPIA (GSK, Pfizer, Astra-Zeneca, Lundbeck).

Common subject inclusion criteria:

(1) DSM-IV/ICD10 diagnosis of unipolar depression of at least moderate severity (alternatively HRSD-17 of 14 or more can be used to define at least moderate depression).

(2) White European ethnicity. (Ethnicity is a major confound in genetic studies due to problems introduced by population stratification. At present sufficient numbers of subjects of non European ethnic groups are not available to be included).

(3) Age 18-80 (with onset of first depression before age 65).

(5) Treatment with either serotonergic antidepressant (citalopram, escitalopram, fluoxetine, sertraline, paroxetine) or noradrenergic antidepressant (nortriptyline, reboxetine).

(5) At least one post-baseline visit with available measure of depression severity.

(6) Available DNA sample

Using the above inclusion criteria, 1719 subjects with available DNA samples have been committed to the work package.

810 GENDEP samples (escitalopram, nortriptyline)* - shipped

512 GENPOD samples (citalopram, reboxetine) - shipped

140 GSK samples (SSRI)

257 PFIZER samples (SSRI)

*** The GENDEP samples include 706 subjects included in a published genome-wide pharmacogenetic analysis[2] and additional 104 subjects that were not included in previous analyses and are being genotyped under NEWMEDs**

Additional samples are being selected from the data bases of LUNDBECK (SSRI, possibly nortriptyline). Astra-Zeneca will contribute samples to a replication project.

Since the GENDEP and GenPod samples have already been shipped, the first analysis can be performed in these two studies only. Second and third rounds of analyses will then include the EFPIA samples as they are becoming available.

Outcomes to be analyzed

The response to antidepressant medication is a complex phenotype that includes changes in various symptoms occurring over a number of weeks. It is typically measured by repeated administration of symptom rating scales such as Hamilton Rating Scale for Depression (HRSD-17), Montgomery and Asberg depression rating scale (MADRS) or Beck Depression Inventory (BDI). At each time-point, a variable proportion of measurement is missing due to loss to follow-up, non-attendance or failure to complete a specific measure. The preferred method to analyse repeated measurements with missing data are approaches such as mixed effect regression models, which allow use of multiple measurements and do not require the imputation of missing values. However, such methods are impractical for genome-wide pharmacogenetic analyses since they are computationally intensive. To enable a computationally feasible genome-wide pharmacogenetic analysis, it is practical to derive a single summary measure that best represents change in depressive symptoms over time. We have considered the following summary measures: absolute reduction in depression severity score over the study duration (e.g. 12 weeks), percentage reduction in depression severity over the study duration, individual intercept estimated in a mixed effect linear regression model (individuals' departure from the average estimate given included covariates).

We propose that we use the same measure as in the published GWAS analysis of the 706 subject already genotyped from the GENDEP study.[2] Here we selected percentage improvement in depression score from baseline to week 12 as a summary outcome measure that reflects change in depressive symptoms over the study period. Percentage change was selected in preference to absolute change as it shows high correlation with end-score (Pearson's r=0.84), low correlation with baseline severity (r=-0.06), and has been shown to better reflect clinician's impression of improvement.[4] In addition, expression of change in terms of percentage of the baseline score is relatively independent of which measure is used and is easily understandable. Also, since improvement in depression severity with treatment is a matter of degree, continuous measures such as percentage improvement are more efficient than cut-off-based dichotomous measures of response or remission.[5, 6]

It has been shown that summary outcome measures can be biased in the presence of missing data if the commonly used last-observation-carried forward procedure is applied.[7, 8] Therefore, we replaced missing week 12 measurements by the best unbiased estimate from a mixed effect linear regression model with fixed effects of linear and quadratic effects of time, baseline severity and random effects of individual and centre of recruitment.[1] This procedure has been shown to be relatively free of bias under a wider range of assumptions.[7, 8]

Since outcome was significantly associated with age and varied across centers of recruitment (at least in the GENDEP study), we propose to adjust the percentage improvement for age and centre. This procedure minimized the risk that minor genetic differences due to population stratification could be spuriously associated with outcome due to between-centre variations in recruitment, assessment and treatment. Adjustment for age is appropriate as genotype does not change with age and any age-related variation in outcome is likely to be due to factors other than genes.

It is possible to score the various outcome measures (HRSD-17, MADRS, BDI) on a single metric using the item response theory[9] and GENDEP as a linking dataset as it has all three measures on more than 9000 occasions (we have the method running, and can do it if item-level data are available). This may be the most accurate method of linking the studies. However, if percentage change is used (all three scales start at a true zero) and outcome is adjusted on age and centre of recruitment, this may not be necessary.

Primary outcome:

1) Quantitative trait: percentage change in primary measure of depression severity (HRSD-17, MADRS or BDI – depending on study) after 12 weeks, with missing data at week 12 replaced with best unbiased estimates from the best fitting mixed effect regression model (excluding any genetic effect) and adjusted on centre of recruitment (a series of binary dummy variables) and age within each study.

Secondary outcomes (to be considered for additional analyses

2) Discrete trait: remission at end-point (HRSD-17 < 8, MADRS <10, BDI < 10)

3) Adverse reactions (a score on adverse effects rating scale or a specific adverse reaction)[10]

4) Discrete trait: treatment-related increase in suicidality.[11, 12]

Genotyping platforms and imputation of genotypes

All datasets are genotyped on Illumina 610quad or Illumina 660 chips, which have identical tag SNP coverage. Therefore, imputation is not necessary for primary analyses. Integration and comparability with samples genotype on other platforms may require genome-wide imputation. Imputation with one of the commonly-used programs (BEAGLE, IMPUTE v1 or v2, MACH) will allow further exploration of interesting regions.

Quality control

The quality control will include SNP genotyping completeness, excluding individuals with high missingness, abnormal heterozygosity, ethnicity admixture, or cryptic relatedness to other individual within the same study (up to 3rd degree relative).

Quality control procedures will be applied in PLINK,[13] first at the level of marker and then at a level of individual. Markers will be excluded if they have a minor allele frequency (MAF) of less than 0.01 as effects of rare markers would be difficult to test and interpret with the present sample size. Markers will be filtered for completeness of genotyping of 99% so that all analyses are performed in a comparable set of individuals (this is more important for a continuous trait analyses than in case-control study, as individuals with extreme trait values contribute disproportionately to an association). Hardy-Weinberg Equilibrim (HWE) will be tested using the exact test in PLINK, but will not be used as a filter as departures from HWE may be expected in a case-only sample.[14] Probability of departures from HWE will be given with all reported markers and any pharmacogenetic association with markers that show substantial HWE departures will be confirmed by individual re-genotyping.

At individual level, genotypes will be first tested for sex mismatch with phenotypic data. Samples with ambiguous genotype sex and outliers on autosomal heterozygozity will be identified for exclusion as these may indicate sample contamination. Related individuals will be ascertained through estimation of identity by descent (IBD) obtained by application of the PLINK --genome procedure[13] to an LD-pruned dataset (same as for analysis of population stratification, see below) and one of each pair of first- or second-degree relatives (the one with less complete data) will be excluded. Finally, genotyping completeness will be assessed for

each individual and outliers with genotyping completeness less than 95% will be excluded from further analyses.

Population stratification

Although recruitment is restricted to individuals of European descent, self-reported ethnicity does not guarantee absence of genetic admixture and significant stratification within European populations has been reported.[15] Therefore, we propose to use principal component analysis applied in EIGENSTRAT[16] to detect population structure and control for it in the analyses. To avoid confounding by local linkage disequilibrium (LD), the principal component analysis will be performed on an LD-pruned dataset excluding known regions of long-range LD.[17] The EIGENSTRAT analysis will be performed iteratively, with removal of obvious outliers that would bias the principal component analysis. The first iteration of EIGENSTRAT will be run with HapMap2 and HapMap3 populations to detect individuals of non-European ancestry. Second iteration will be run with only study data. After exclusion of outliers, detected significant principal components will be explored for association with centre of recruitment and significant principal components that will be used as covariates in the main analyses. A single EIGENSTRAT analysis will be conducted with samples from all studies. If significant heterogeneity remains with multiple separate clusters corresponding to different studies being separated on more than one principal component, the overall statistical approach will need to be reconsidered and studies may be analyzed separately and the results combined using a meta-analytic method (see below).

Primary genome-wide analyses

The primary analysis will be carried out as joint analyses of individual data from multiple studies (megaanalysis; conditional on EIGENSTRAT results). To obtain estimates that are generalizable across studies and are not biased by between-study design differences, the outcome measure in each study will be adjusted on centre of recruitment and age (residuals centered at zero) and study will be used as covariate (a series of dummy binary covariates).

The association between genotypes and adjusted percentage improvement in depression score will be performed using linear regression with an additive genetic model including significant principal components suggestive of population stratification and study identifier as covariates to control for population stratification, applied in PLINK.[13]

Four tests will be performed:

(1) Association between genotype and outcome across the whole sample to identify genetic variants associated with improvement irrespective of the type of antidepressant (and including placebo effect);

(2) Association between genotype and outcome within subjects treated with SSRI antidepressants (citalopram, escitalopram, …) to identify genetic variants associated with outcome of treatment with a serotonin reuptake inhibitor;

(3) Association between genotype and outcome within subjects treated with noradrenaline-reuptake inhibiting antidepressants (nortriptyline, reboxetine) to identify genetic variants associated with outcome of treatment with this noradrenaline-reuptake inhibitor;

(4) Interaction between genotype and antidepressant mode of action (serotonergic/noradrenergic) to identify genetic variants that predict differential outcome of treatment with SSRI versus SNRI.

For each analysis, the quality of control for population stratification effects will be checked by visual inspection of quantile-quantile (QQ) plots and calculation of the genomic control inflation factor λ (lambda).[18]

Genome-wide significance was set at the generally accepted level of $p < 5*10^{-8}$.[19, 20]

Suggestive significance (reporting) threshold will be set at $p < 5*10^{-6}$.

In addition, to estimate the posterior probability of true positive findings in the context of multiple non-independent tests, false discovery rate q-values will be calculated using the step-up procedure described by Benjamini & Hochberg.[21] The q-values calculated in this way have been shown to retain desirable properties for multiple related tests in genome-wide association studies[22, 23] and can be interpreted as posterior probabilities of no association at a given locus.[24] A q-value of less than 0.1 has been proposed as a criterion for significance in genome-wide studies.[25] Therefore, we include q-value alongside p-values for results of interest and we report all associations with a q-value of 0.1 in any of the four primary analyses.

Example of PLINK code (Quantitative trait: MADRS percentage reduction at 12 weeks)

plink --bfile gendep_data_after_QC --pheno hdrs_perc_change_wk12_ adjusted --covar hcovar_un --covar-name pc1,pc2,pc3 --hide-covar --out gendep_md12perc_res --linear –adjust --ci 0.95

Meta-Analyses (optional)

If the principal components analysis using EIGENSTRAT detects gross stratification by study, which cannot be corrected for through use of principal components within a linear regression model, the primary analyses will be performed separately for each study and results will then be combined in a meta-analysis.

Primary

(1) Overall meta-analysis irrespective of what treatment was used across the samples.

(2) Drug-specific meta-analysis (SSRI/SNRI).

If more than two studies are included, random effects would be most appropriate, rather than fixed effects, as the primary statistic of merit. Meta-analysis can be completed using PLINK.

Genome-wide significance threshold: $p \leq 5 * 10^{-8}$

Suggestive significance (reporting) threshold: $p \leq 5 * 10^{-6}$

Secondary analyses

(1) Analysis of candidate genes, with appropriate correction for multiple testing. This will include a consensus list of candidate genes the results of which are of interest even if these do not reach genome-wide reporting criteria.

(2) Polygenic score analysis – this will give an indication of the common information for genetic variability betwee studies. E.g., between GENDEP and GenPod:

Forty genetic profile scores will be created based on the four analyses in the GENPOD sample, including between 31 and 549,919 SNP markers based on 10 progressively increasing thresholds of p-values in the

GENPOD analyses ($p < 0.0001$, 0.001, 0.01, 0.05, 0.1, 0.2 0.3, 0.4, 0.5, 1). The genetic scores will be created as a linear combination of tested allele number of each GENDEP participant multiplied by the regression coefficient [beta of ln(OR)] obtained from the GenPod analysis. These 40 genetic profile scores will then be tested as predictors of outcome in GENDEP, using logistic and linear regression for categorical and quantitative outcomes respectively.

(3) Megavariate prediction using machine learning. Machine learning methods will be applied to optimise their prediction algorithm based on one study and applied it to another study (e.g. GENDEP – GenPod) to establish the generalisability of this prediction. This method is in development and is likely to use Gaussian processes (for probabilistic predictions of continuous outcomes) and either unsupervised or supervised learning (using prior information about the genome).

Data sharing

The principles inherent in the IMI initiative are that data generated in the NEWMEDS project are used as much as possible and that results are published without delays. Following these principles, we propose the following data sharing rules:

(1) Phenotypic data (depression severity measurements, sex, age, centre of recruitment, ethnicity, adverse reactions) will be made available by the dataset owners to the team at King's College London (Peter McGuffin, Cathryn Lewis, Rudolf Uher), who will be responsible for the primary analyses. These data will be only used for the purpose specified within the NEWMEDS project agreements.

(2) Genotyping data (genome-wide genotyping) will be made available to the dataset owners, who are free to use these data for additional projects and studies involving that datasets.

(3) Genotype and phenotype exchange across studies is possible for the purpose of additional projects proposed by any collaborator conditional on the approval of dataset owners.

Publication and Authorship

All results should be published without unnecessary delays. Individuals involved in the generation of both phenotypic and genetic data will be involved in the publication process and offered the opportunity for authorship (either individual or as part of a consortium).

Publications using the genetic data generated through the NEWMEDS project should include the following co-authors: Michel Guipponi, Elizabeth Neidhart, Rudolf Uher, (+ PostDoc on the NEWMEDS study, currently being recruited), Sarah Cohen-Woods, Cathryn M. Lewis, and Peter McGuffin.

Publications using phenotypic and/or genetic data from the GENDEP project should also include the following co-authors (individually or as the GENDEP consortium): Katherine J Aitchison, Anne Farmer, Ian Craig, Marcella Rietschel, Ole Mors, Astrid Zobel, Joanna Hauser, Neven Henigberg, Dejan Kozel, Anna Placentino, Daniel Souery.

Publications using phenotypic and/or genetic data from the GenPod project should include the following co-authors: Glyn Lewis, Michael O'Donovan, Tim Peters, David Evans, ….(GLYN, PLEASE COMPLETE / ADAPT THE LIST AS YOU JUDGE SUITABLE).

Publications using phenotypic and/or genetic data from the PFIZER projects should include the following co-authors: Xialan Hu,

Publications using phenotypic and/or genetic data from the GSK projects should include the following co-authors: Enrico Domenici,

Publications using phenotypic and/or genetic data from the Astra-Zeneca projects should include the following co-authors: Jayne C Fox

Publications using phenotypic and/or genetic data from the Lundbeck projects should include the following co-authors: Francois Menard

Rudolf Uher

14th April 2010

REFERENCES

(1) Uher R, Maier W, Hauser J et al. Differential efficacy of escitalopram and nortriptyline on dimensional measures of depression. *Br J Psychiatry* 2009;194(3):252-9.

(2) Uher R, Perroud N, Ng MY et al. Genome-Wide Pharmacogenetics of Antidepressant Response in the GENDEP Project. *Am J Psychiatry* 2010.

(3) Thomas L, Mulligan J, Mason V et al. GENetic and clinical Predictors Of treatment response in Depression: the GenPod randomised trial protocol. *Trials* 2008;9(1):29.

(4) Leucht S, Kane JM, Etschel E, Kissling W, Hamann J, Engel RR. Linking the PANSS, BPRS, and CGI: clinical implications. *Neuropsychopharmacology* 2006;31(10):2318-25.

(5) Royston P, Altman DG, Sauerbrei W. Dichotomizing continuous predictors in multiple regression: a bad idea. *Stat Med* 2006;25(1):127-41.

(6) Streiner DL. Breaking up is hard to do: the heartbreak of dichotomizing continuous data. *Can J Psychiatry* 2002;47(3):262-6.

(7) Mallinckrodt CH, Clark WS, David SR. Accounting for dropout bias using mixed-effects models. *J Biopharm Stat* 2001;11(1-2):9-21.

(8) Lane P. Handling drop-out in longitudinal clinical trials: a comparison of the LOCF and MMRM approaches. *Pharmaceutical Statistics* 2008;7(2):93-106.

(9) Uher R, Farmer A, Maier W et al. Measuring depression: comparison and integration of three scales in the GENDEP study. *Psychological Medicine* 2008;38(2):289-300.

(10) Uher R, Farmer A, Henigsberg N et al. Adverse reactions to antidepressants. *Br J Psychiatry* 2009;195(3):202-10.

(11) Perroud N, Aitchison KJ, Huezo-Diaz P et al. Genetic predictors of suicidality during antidepressant treatment in GENDEP. *Submitted* 2008.

(12) Perroud N, Uher R, Marusic A et al. Suicidal ideation during treatment of depression with escitalopram and nortriptyline in genome-based therapeutic drugs for depression (GENDEP): a clinical trial. *BMC Med* 2009;7:60.

(13) Purcell S, Neale B, Todd-Brown K et al. PLINK: a tool set for whole-genome association and population-based linkage analyses. *Am J Hum Genet* 2007;81(3):559-75.

(14) Wittke-Thompson JK, Pluzhnikov A, Cox NJ. Rational inferences about departures from Hardy-Weinberg equilibrium. *Am J Hum Genet* 2005;76(6):967-86.

(15) Seldin MF, Shigeta R, Villoslada P et al. European population substructure: clustering of northern and southern populations. *PLoS Genet* 2006;2(9):e143.

(16) Price AL, Patterson NJ, Plenge RM, Weinblatt ME, Shadick NA, Reich D. Principal components analysis corrects for stratification in genome-wide association studies. *Nat Genet* 2006;38(8):904-9.

(17) Price AL, Weale ME, Patterson N et al. Long-range LD can confound genome scans in admixed populations. *Am J Hum Genet* 2008;83(1):132-5.

(18) Bacanu SA, Devlin B, Roeder K. Association studies for quantitative traits in structured populations. *Genet Epidemiol* 2002;22(1):78-93.

(19) Dudbridge F, Gusnanto A. Estimation of significance thresholds for genomewide association scans. *Genet Epidemiol* 2008;32(3):227-34.

(20) Pe'er I, Yelensky R, Altshuler D, Daly MJ. Estimation of the multiple testing burden for genomewide association studies of nearly all common variants. *Genet Epidemiol* 2008;32(4):381-5.

(21) Benjamini Y, Hochberg Y. Controlling the false discovery rate: a practical and powerful approach to multiple testing. *J R Stat Soc Ser B* 1995;57(1):289-300.

(22) Sabatti C, Service S, Freimer N. False discovery rate in linkage and association genome screens for complex disorders. *Genetics* 2003;164(2):829-33.

(23) Storey JD, Tibshirani R. Statistical significance for genomewide studies. *Proc Natl Acad Sci U S A* 2003;100(16):9440-5.

(24) Kall L, Storey JD, MacCoss MJ, Noble WS. Posterior error probabilities and false discovery rates: two sides of the same coin. *J Proteome Res* 2008;7(1):40-4.

(25) Sullivan PF, de Geus EJ, Willemsen G et al. Genome-wide association for major depressive disorder: a possible role for the presynaptic protein piccolo. *Mol Psychiatry* 2008.
